# Dialkylaminoalkylation of β-ketosulfones via ring-opening of 3-sulfonylpyrrolidines

**DOI:** 10.3762/bjoc.22.26

**Published:** 2026-03-03

**Authors:** Evgeny M Buev, Alexander V Pavlushin, Vladimir S Moshkin, Vyacheslav Y Sosnovskikh

**Affiliations:** 1 Institute of Natural Sciences and Mathematics, Ural Federal University, 620000 Ekaterinburg, Russian Federation

**Keywords:** aminoalkylation, aminosulfones, ketosulfones, pyrrolidines

## Abstract

Herein, a three-step method for the simultaneous dialkylaminoethylation and heteromethylation of active methylene β-ketosulfones, promoted by a leaving benzoyl group, is proposed. The primary domino reaction of sarcosine, paraformaldehyde, and β-ketosulfones affords 3-sulfonyl-3-benzoylpyrrolidines in good to high yields. Further treatment with alkyl halides and heating of the quaternary ammonium salt in the presence of a O-, S- or N-nucleophile with cesium carbonate leads to a retro-Claisen reaction, ring-opening and Michael addition. The resulting novel 4-Nu-3-sulfonylbutan-1-amines are isolated in moderate to excellent overall yields. The reduction of the obtained products with LiAlH_4_ leads to a substitution of the attached nucleophilic moiety with hydrogen in good yields.

## Introduction

**Figure 1:**
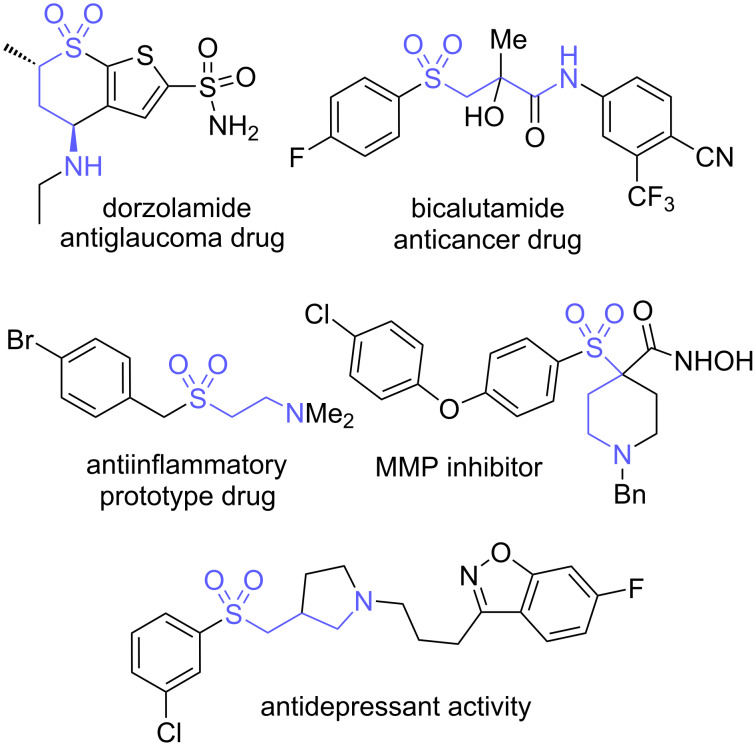
Valuable amino sulfonic acids and aminosulfones.

Sulfones and sulfonic acids are valuable structural motifs in organic chemistry, notable for their broad spectrum of pharmacological activities [1–3] and synthetic potential [4–5]. Also noteworthy are γ-aminosulfones, which form the backbone of important pharmaceuticals including the antiglaucoma drug dorzolamide [6], acamprosate for alcohol craving suppression [7], and bicalutamide, an antiandrogen cancer treatment [8–9] (Figure 1). Moreover, aminosulfones remain a privileged scaffold in the ongoing development of new methods for the synthesis of pharmaceuticals [10–12], with multiple research compounds showing promising bioactivities such as MMP inhibition [13], antiinflammatory effects [14], сoagulation enzyme factor (FXa) inhibition [15] and antidepressant properties [16].

Considering the approaches to the synthesis of γ-aminosulfones, we focused our attention on the implementation of an aminoalkylation as a powerful and versatile tool for the synthesis of aliphatic amines [17–19]. Among the various types of aminoalkylations, aminomethylation reactions have been the most extensively studied and applied significantly to aromatic [20] and CH-acidic compounds [21], alkanes [22–23], olefines [24–25] and electron-withdrawing alkenes [26–27].

This rapidly growing research field is offering diverse methodologies that enable the introduction of either protected nitrogen atoms or aliphatic amine groups. Such diversity has greatly expanded the synthetic arsenal available for constructing aminomethylated compounds.

**Figure 2:**
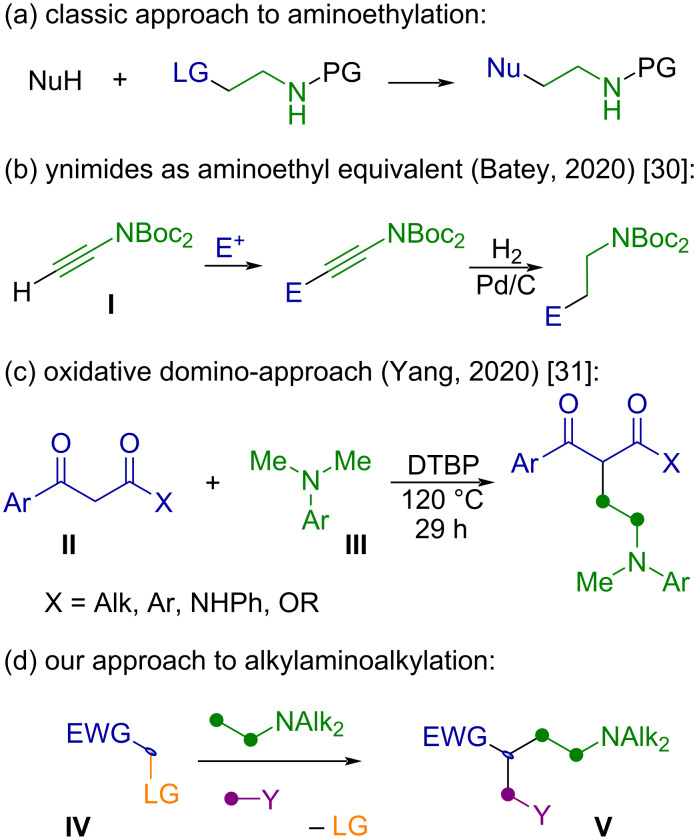
Selected approaches to aminoethylation.

In contrast, methods allowing an aminoethylation process is commonly achieved by a classic nucleophilic substitution of a leaving group at the β-position relative to the N-protected nitrogen (Figure 2a). While this approach addresses most needs of targeted synthesis, developing alternative methods from inexpensive starting materials via domino-processes, allowing the fast increase of the molecular complexity, remains significant. Unlike the classical aminoethylation of nucleophilic compounds [28–29], Batey et al. recently developed a two-step approach consisted in addition of terminal ynimides **I** to various electrophiles with subsequent reduction of the C≡C bond (Figure 2b) [30]. At the same time, Yang et al. presented a domino radical reaction of CH_2_-active compounds **II** and *N*,*N*-dimethylanilines **III** promoted by di-*tert*-butyl peroxide (DTBP), allowing the formation of the ethylene link via a domino-process of the methylenation and subsequent aminomethyl radical addition (Figure 2c) [31].

In the continuation of our work on application of pyrrolidines as sacrificial framework for the alkylaminomethylation of enones [32–33] we turned our attention to active methylene compounds **IV** as precursors of pyrrolidines. Preliminary experiments demonstrated that the quaternary ammonium salt of 3,3-dibenzoylpyrrolidine, derived from dibenzoylmethane, undergoes ring-opening in the presence of a nucleophile and sodium methoxide via a retro-Claisen reaction [33]. This process yields 4-(dimethylamino)-2-heteromethyl-1-phenylbutan-1-one scaffold **V** (EWG = COPh; Y = OR, SR, NR; LG = H or COPh). We envisioned that such an approach is useful for the simultaneous introduction of aminoethyl and heteromethyl moieties at other active methylene compounds (Figure 2d). In this work, we focused on such dual aminoalkylative difunctionalization of β-ketosulfones [4,34] through a three-step method for the synthesis of *N*,*N*-dialkyl-3-(sulfonyl)butan-1-amines.

## Results and Discussion

**Scheme 1:**
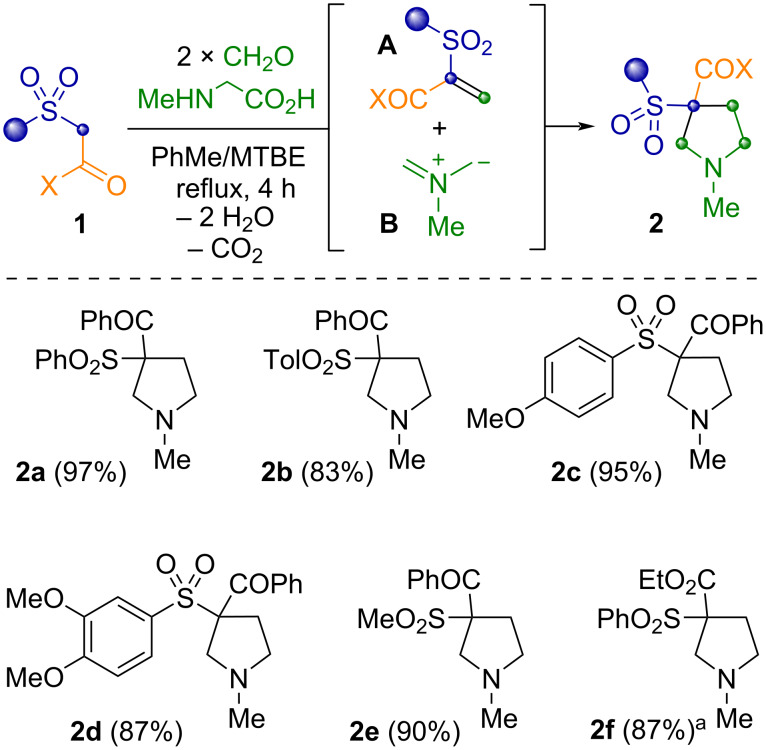
Pyrrolidination of β-ketosulfones. Reaction conditions: β-ketosulfones (4.0 mmol), sarcosine (1.2 equiv), (CH_2_O)*_n_* (2.5 equiv), PhMe/MTBE (17/3 mL), reflux 4 h. ^a^2.5 equiv of sarcosine was used.

We began our survey with the examination of the pyrrolidination reaction [35–36] of β-ketosulfones (Scheme 1). This domino reaction of an active methylene compound, *N*-methylglycine and paraformaldehyde consists in the simultaneous generation of two reactive intermediates: terminal alkene **A** and *N*-methylazomethine ylide **B**, and final [3 + 2] cycloaddition. It was found that the latter approach depends on CH-acidity of the active methylene compounds and performs well with the acidic substrates in the range of p*K*_a_ 12–17 (in DMSO) [37]. Considering that the p*K*_a_ of 1-phenyl-2-(phenylsulfonyl)ethan-1-one **1** is close to that interval (p*K*_a_ 11.4, DMSO) we estimated its smooth incorporation in the process. Nevertheless, previously developed conditions for the pyrrolidination reaction provided insufficient results. Refluxing a mixture of ketosulfone **1**, a two-fold excesses of *N*-methylglycine and paraformaldehyde in benzene with a Dean–Stark trap for 4 h yields incomplete conversion of the substrate (Scheme 1, product **2a**, 60%). However, we did not observe the resinification of the reaction media and hence we decided to increase the reaction temperature. Thus, refluxing the reagents in the PhMe/MTBE mixture (v/v 17/3, approximately 100 °C) for 4 h led to complete conversion of the ketosulfone and formation of 3-phenylsulfonyl-3-benzoyl-*N*-methylpyrrolidine (**2a**) in almost quantitative yield.

Despite that [3 + 2] cycloaddition of azomethine ylides at various vinyl sulfones were well studied [38–41], a method for the preparation of 3-sulfonylpyrrolidines from β-ketosulfones was previously unknown. We synthesized a number of pyrrolidines **2a**–**f** in 83–97% yield, which were purified by an acid-base extraction (Scheme 1). Altering the substituents at the sulfonyl group made no significant impact on the yield of the reaction. An application of β-ethoxycarbonylsulfone lowered the yield of the pyrrolidine **2f** (53%), apparently due to the lower acidity and activity of its methylene group, however, increasing the excess of azomethine ylide precursors (2.5:2.5 equiv sarcosine/CH_2_O) led to 87% yield.

The obtained pyrrolidines represent a class of labile compounds with a strained C2–C3 bond. The presence of the benzoyl and sulfonyl groups able to eliminate [5,42] and generate an anion at the 3rd position should promote Hofmann elimination. To enhance the leaving ability of the nitrogen atom we prepared ammonium salts **3** of the obtained pyrrolidines via treatment with alkyl halides. Since the preparation of quaternary ammonium salts of pyrrolidines is straightforward, we employed a one-pot method for its synthesis by adding the alkyl halide directly to the cooled reaction mixture of the formed pyrrolidine without its isolation and purification, that provided higher yields of the quaternary compounds **3** rather than step by step approach.

Further, we focused our attention on the search for the reaction conditions for the ring-cleavage of the obtained quaternary compound **3a**. An application of sodium methylate, suitable for the 3,3-dibenzoylpyrrolidine salt [33], provided only 45% conversion of the substrate (Table 1, entry 1). Treating a methanolic solution of quaternary compound **3a** with milder base such as cesium carbonate at 25 °C for 96 h provided higher yield (Table 1, entry 2, 70%). However, heating this mixture at 65 °C for 24 h in a sealed vial gave pure desired 4-methoxy-*N*,*N*-dimethyl-3-(phenylsulfonyl)butan-1-amine (**4a)** in 90% yield and 83% overall yield starting from ketosulfone **1a** (Table 1, entry 4).

**Table 1:** Synthetic sequence and optimization of the reaction conditions.^a^

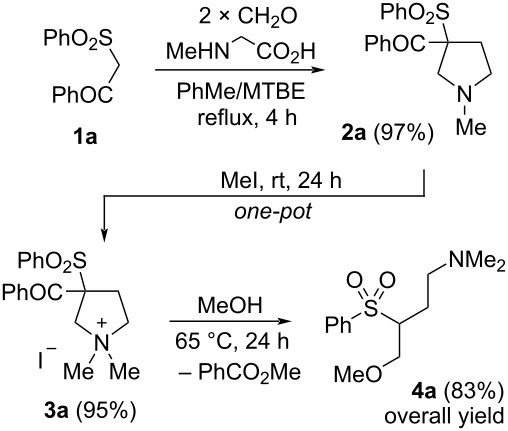		

	Ring-cleavage step conditions	Result

1	MeONa (1.2 equiv), MeOH, 25 °C, 24 h	**4a** (45%)
2	Cs_2_CO_3_ (2.1 equiv), MeOH, 25 °C, 96 h	**4a** (70%)
3	Cs_2_CO_3_ (2.1 equiv), MeOH, 65 °C, 15 h	**4a** (74%)
**4**	**Cs** **_2_** **CO** **_3_** ** (2.1 equiv), MeOH, 65 °C, 24 h**	**4a (90%)** **^b^**
5	CO_2_Et instead of COPh as a leaving group KOH (4.2 equiv), MeOH, 65 °C, 46 h	**4a** (89%)^b,c^

^a^The reaction sequence can be stopped at any stage to isolate the intermediate products **2** and **3**. The yield of **3a** is overall yield calculated on starting β-ketosulfone **1a**. ^b^Isolated yield. ^c^Overall yield is 77%.

In attempt to minimize the size of the leaving group we applied a ethoxycarbоnyl moiety instead of the benzoyl group. Thus, heating the dimethylammonium salt of pyrrolidine **2f** with an excess of KOH in MeOH for 46 h provided the same product **4a** in 89% yield (Table 1, entry 5). While it is possible to use an ester group for the cleavage of 3-sulfonylpyrrolidines, it required a longer reaction time and provided a slightly lower overall product yield (77%) compared to the use of the benzoyl group.

With an optimized synthetic route in hand – comprising a multicomponent reaction of ketosulfone, sarcosine, and paraformaldehyde in PhMe/MTBE, followed by treatment of the reaction mixture with an alkyl halide, and subsequent heating of the precipitated quaternary ammonium salt in the presence of Cs₂CO₃ and a nucleophile – we proceeded to apply this methodology to other β-ketosulfones.

**Scheme 2:**
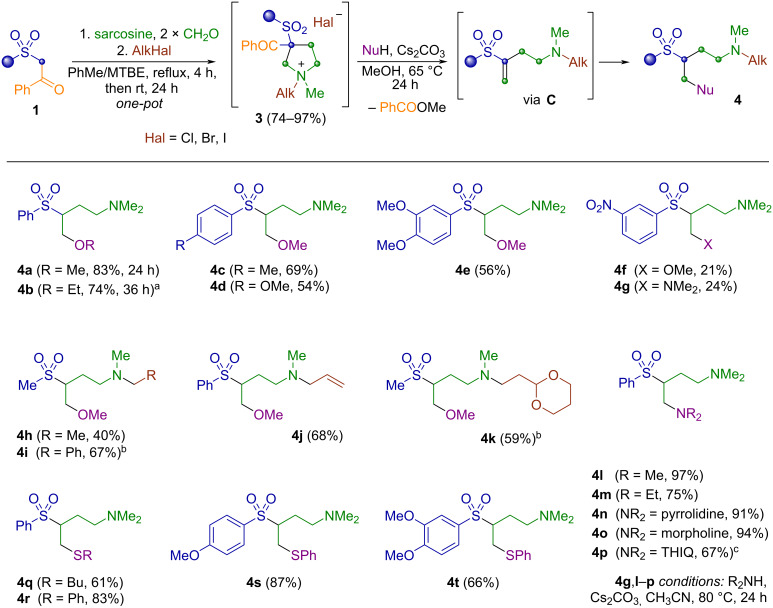
Scope of the reaction. Reaction conditions: sulfone **1** (1.0 mmol), sarcosine (1.2 equiv), (CH_2_O)*_n_* (2.5 equiv), PhMe/MTBE (17/3 v/v), Dean–Stark trap, reflux, 4 h, cooled to rt and then AlkHal (1.5 equiv), rt, 24 h (see Supporting Information File 1 for details). Quaternary compound **3**, Cs_2_CO_3_ (2.1 equiv), MeOH, 65 °C, 24 h for **4a**–**f**,**h**–**k,q**–**t** or CH_3_CN, 80 °C, 24 h for **4g,l**–**p**. ^a^Ethanol 95% was used instead of methanol, heating at 80 °C for 36 h. ^b^The quaternization with AlkHal (1.5 equiv) was performed at 70 °C for 7 days. ^c^THIQ = 1,2,3,4-tetrahydroisoquinolin-2-yl.

Next, we investigated the influence of both the substituent at the sulfonyl group and the alkyl chain introduced at the nitrogen atom. It turned out that the electron-donating groups at the aryl ring or a methyl group at the sulfonyl moiety lowered the yield and the corresponding 4-methoxy-*N*,*N*-dialkyl-3-(sulfonyl)butan-1-amines **4c**–**e**,**h** were isolated in 40–69% yields (Scheme 2). Introduction of the *m*-nitro substituent led to partial resinification during the ring-cleavage step and significant decrease in the reaction outcome (products **4f**,**g**). We have demonstrated that ethyl bromide, benzyl chloride, allyl bromide, and even the bulky 2-(2-bromoethyl)-1,3-dioxane, which contains a masked aldehyde moiety, are suitable alkylating agents for the process, providing products **4h**–**k** in good overall yields. The possibility to vary alkyl halide in the second stage of the synthesis opens opportunities for further modifications and the use of the obtained compounds as building blocks.

We also examined other nucleophiles suitable for the described domino-sequence. Thus, application of ethanol as solvent resulted in a mixture of the desired product **4b** with the corresponding intermediate **C** (NMR ratio 88:12), however, longer heating for 36 h provided ethoxy product **4b** in 74% yield. Heating quaternary ammonium salt **3a** with an excess of secondary amines in acetonitrile enabled concurrent aminomethylation and aminoethylation of the starting β-ketosulfone in good to excellent overall yields. Notably, even application of aqueous solution of dimethylamine did not affect the process and bis-dimethylamino product **4l** was readily isolated in 97% yield. The obtained 2-(phenylsulfonyl)butane-1,4-diamines **4l**–**p** represent the biogenic amine putrescine scaffold, incorporating dialkylamino or azaheterocyclic motifs such as pyrrolidine, piperidine, morpholine, or 1,2,3,4-tetrahydroisoquinoline (THIQ). A special curiosity was associated with the use of mercaptans as nucleophiles for the synthesis of aminobutanes **4** containing both sulfide and sulfone moieties. To our delight, an excess of butylmercaptan or thiophenol readily afforded products **4q**–**t** in 61–87% yields.

**Scheme 3:**
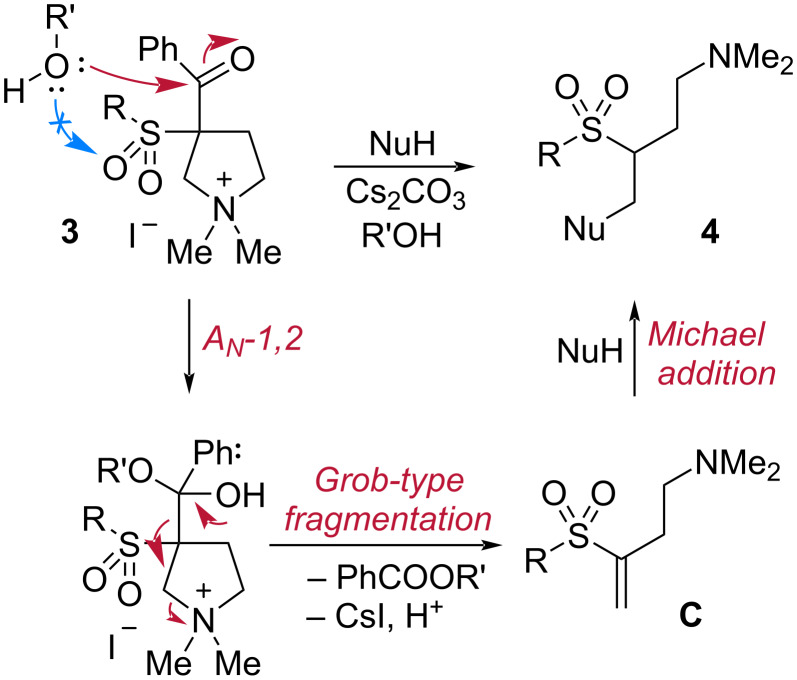
The presumable mechanism of the domino process.

It should be noted that the leaving 3-benzoyl group formed a methyl benzoate when the reactions were performed in methanol. We also observed the formation of *S*-butyl benzothioate in the reaction mixture in case of **4q**. These by-products were readily removed by an acid-base extraction or column chromatography. On the other hand, upon performing the reaction with amines in acetonitrile we did not observe the formation of benzamides. Presumably, debenzoylation occurred in basic media with water traces and cesium benzoate was discarded upon work-up. The presumable mechanism of the domino-process consists in nucleophilic addition of the alcohol, mercaptan or water (in case the reaction was performed in CH_3_CN) at the benzoyl group (Scheme 3). The subsequent ring-opening of the pyrrolidine ring can proceed via two possible pathways. First path is the tandem Grob-type fragmentation. Second is a step-wise route through retro-Claisen reaction, formation of the carbanion at the α-position to sulfonyl group, and Hofmann elimination of the quaternary nitrogen at the β-position. Both routes lead to the formation of terminal vinyl sulfone **C**, which reacts with the nucleophile present in the reaction media to form 4-Nu-3-(sulfonyl)butan-1-amines **4**.

**Scheme 4:**
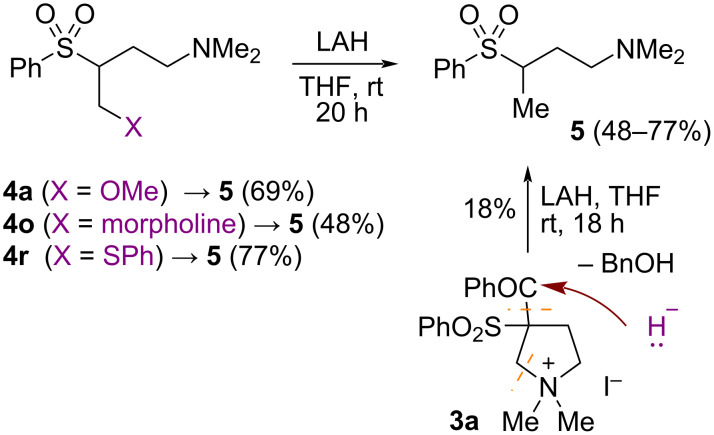
Reductive elimination of the nucleophiles.

An attempt to obtain 3,4-bis(phenylthio)butan-1-amine via reduction of the sulfonyl group [43–44] of the product **4r** with LiAlH_4_ gave an unexpected reductive elimination of the thiophenol moiety furnishing *N*,*N*-dimethyl-3-(phenylsulfonyl)butan-1-amine (**5**) in 77% yield (Scheme 4). This observation prompted us to examine methoxy and morpholine adducts **4a** and **4o** in the same process that resulted in the isolation of sulfonylamine **5** in 69 and 48%, respectively. The literature search revealed that previously Langler and co-authors observed the similar process of the reductive elimination of chlorine or chlorosulfonyl groups at the β-position to sulfone [45]. We also examined the starting ammonium salt **3a** in the reductive ring-cleavage. As the result, butanamine **5** was formed in 18% yield along with benzylic alcohol. An increase of the reaction temperature resulted in the formation of a complex mixture with only traces of product **5**. Nevertheless, the sequence of the nucleophilic ring-cleavage of the pyrrolidine ammonium salt **3** with alcohol, amine or thiol and the subsequent reduction with LiAlH_4_ serves as a formal reductive cleavage of the N–C2 bond of the pyrrolidines **2**. In this way, the hydride anion was formally added to the list of possible nucleophiles (H-, N-, O-, S-).

## Conclusion

In conclusion, we developed a practical route to the novel skeleton of *N*,*N*-dialkyl-3-(sulfonyl)butan-1-amines from β-ketosulfones. The proposed approach exploits pyrrolidines as sacrificial framework and two domino processes used for assembling of the pyrrolidine ring and for the ring-cleavage of it. Thus, one-pot pyrrolidination of β-ketosulfones and alkylation combined with the subsequent debenzoylative ring-cleavage allows for wide range of the incorporated reagents, that allows a broad functionalization of the 3-(sulfonyl)butan-1-amine with various substituents and their utilization as building blocks. We believe this approach has promising features for further development and pharmaceutical applications.

## Supporting Information

File 1 Experimental section, characterization data and copies of spectra.

## Data Availability

All data that supports the findings of this study is available in the published article and/or the supporting information of this article.
