# Supplementary material for: Dialkylaminoalkylation of β-ketosulfones via ring-opening of 3-sulfonylpyrrolidines
Source: Beilstein J Org Chem. 2026 Mar 3;22:383–9. doi: 10.3762/bjoc.22.26 (PMC12973443; doi:10.3762/bjoc.22.26)
Supplement: File 1 — Experimental section, characterization data and copies of spectra. [file Beilstein_J_Org_Chem-22-383-s001.pdf]

BEILSTEIN JOURNAL OF ORGANIC CHEMISTRY

Supporting Information

for

Dialkylaminoalkylation of β-ketosulfones via ring-opening of 3-sulfonylpyrrolidines

Evgeny M. Buev, Alexander V. Pavlushin, Vladimir S. Moshkin
and Vyacheslav Y. Sosnovskikh

Beilstein J. Org. Chem. **2026,** *22,* 383–389. doi:10.3762/bjoc.22.26

Experimental section, characterization data and copies of spectra

License and Terms: This is a supporting information file under the terms of the Creative Commons Attribution License (https://creativecommons.org/licenses/by/4.0). Please note that the reuse, redistribution and reproduction in particular requires that the author(s) and source are credited and that individual graphics may be subject to special legal provisions.
The license is subject to the *Beilstein Journal of Organic Chemistry* terms and conditions: (https://www.beilstein-journals.org/bjoc/terms)

Table of Contents

1. Material and methods S2
2. Experimental procedures S3
3. Analytical data of products S5
4. ^{1}H and ^{13}C NMR spectra of the synthesized compounds S15

1. Materials and methods:

Starting materials, reagents, and solvents were purchased from commercial sources and used as received unless stated otherwise. All solvents used were dried and distilled per standard procedures. Dry cesium carbonate was applied. Alkyl halogenides were obtained from the commercial sources and were used after distillation. Starting ketosulfones are known compounds and were prepared according to the literature procedures. Purification of the reaction products was carried out by flash column chromatography using silica gel (40–63 μm, ASTM). A mixture of chloroform/ethanol (95%) was used as eluent. Analytical studies were carried out using equipment at the Center for Joint Use 'Spectroscopy and Analysis of Organic Compounds' at the Postovsky Institute of Organic Synthesis of the Russian Academy of Sciences (Ural Branch) and the Laboratory of Complex Investigations and Expert Evaluation of Organic Materials of the Center for Joint Use at the Ural Federal University. ^{1}H and ^{13}C NMR spectra were recorded at 400/500 and 126 MHz, respectively; $CDCl_3$ was used as solvents. The chemical shifts are reported in ppm relative to internal standard TMS or to residual signals of the solvent for ^{13}C ($CDCl_3$, 77.16 ppm). The HRMS spectra were obtained using TOF mass spectrometer. Electrospray ionization with direct sample inlet (flow rate 240 μL/h) was used. The mass spectrometer was operating in positive mode in the mass range of 50–1550 Da.

2. General procedure:

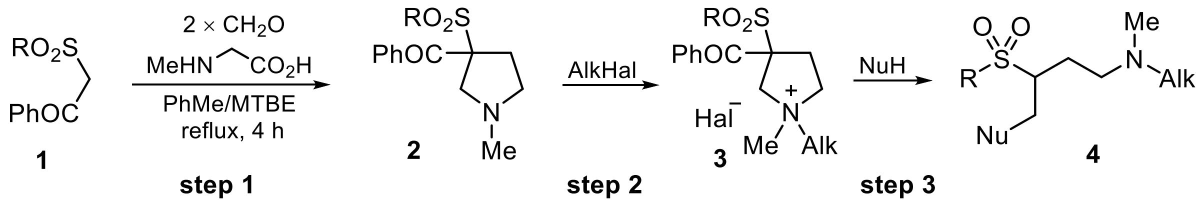

Step 1: A mixture of β-ketosulfone (4.0 mmol, 1.0 equiv), finely ground *N*-methylglycine (427 mg, 4.8 mmol, 1.2 equiv) and paraformaldehyde (300 mg, 10.0 mmol of formaldehyde, 2.5 equiv) was refluxed in a mixture of dried PhMe (17 mL) and MTBE (3 mL) in a 75 mL round-bottom flask fitted with a Dean-Stark trap (filled with PhMe) for 4 h. The resulting mixture was cooled to room temperature and undissolved unreacted excess of *N*-methylglycine was filtered off. (**NOTE**: at this step pyrrolidines **2** can be isolated if needed via an acid-base work-up to provide analytically pure compounds. See **acid-base work-up procedure** below).

Step 2: Corresponding alkyl halide (6.0 mmol, 1.5 equiv) was added dropwise at room temperature to the resulting solution under constant stirring and the stirring continued overnight at room temperature for 15–24 h in case of methyl iodide, ethyl iodide and allyl bromide. In case of benzyl chloride and 2-(2-bromoethyl)-1,3-dioxane the reaction was carried out at 70 °C for 7 days. The formed precipitate was filtered, washed with PhMe several times and dried at 65 °C to give corresponding ammonium salt **3**. Ammonium salt was used further without additional purification.

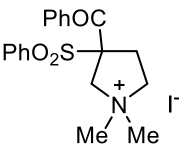

97%

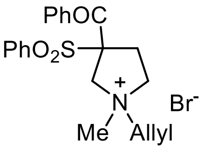

100%

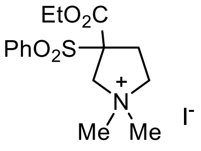

87%

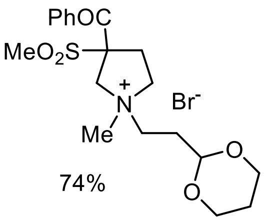

74%

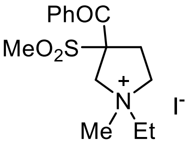

91%

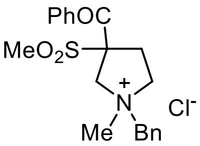

84%

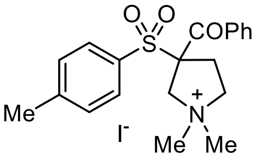

96%

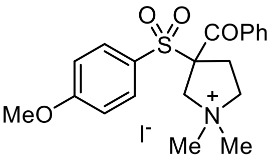

76%

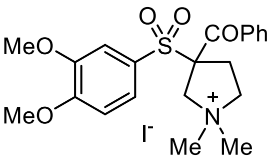

91%

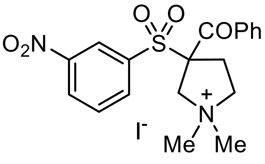

80%

Step 3:

Procedure for the ring-cleavage with **alcohol (products 4a–f,h–k) or mercaptan (products 4q–t)**:

Corresponding quaternary ammonium salt (0.5 mmol, 1.0 equiv), dry cesium carbonate (341 mg, 1.05 mmol, 2.1 equiv), MeOH (4 mL) were sequentially added to the 10 mL pressure microwave vial equipped with magnetic stirrer. Vial was sealed with cap and heated in an oil bath at 65 °C for 24 h under vigorous stirring.

In case of mercaptan products **4q**–**t**: butane-1-thiol (3.0 equiv, 1.5 mmol) or thiophenol (2.0 equiv, 1.0 mmol) were added to the starting mixture.

Procedure for the ring-cleavage with **amine (products 4g,l–p)**:

Corresponding quaternary ammonium salt (0.5 mmol, 1.0 equiv), dry cesium carbonate (341 mg, 1.05 mmol, 2.1 equiv), CH_3CN (4 mL) and amine (1.3–5.0 equiv) were sequentially added to the 10 mL pressure microwave vial equipped with magnetic stirrer. Vial was sealed with cap and heated in an oil bath at 80 °C for 24 h under vigorous stirring.

Work-up: The vial was cooled to room temperature and opened in a fume hood. The mixture was diluted with water (15 mL), extracted with CH_2Cl_2 (3 × 10 mL). Combined organic extracts were washed with water (3 × 10 mL), brine (10 mL), dried over sodium sulfate and evaporated under reduced pressure to give the desired product **4**. The latter was purified by a column chromatography or the acid-base work-up.

Acid-base work-up for the isolation of pyrrolidines **2** and purification of **products 4a**,**c**–**j**,**r**:

The residue was extracted with cold 2 M HCl (12 mL). Water phase was washed with PhMe (2 × 7 mL), basified with $NaHCO_3$ to pH = 8–9 and extracted with CH_2Cl_2 (3 × 7 mL). Combined organic extracts were washed with H_2O (10 mL), brine (10 mL) and dried over anhydrous Na_2SO_4. Solvent was evaporated in vacuo and the product, if necessary, was purified by column chromatography.

Procedure for the ring opening of the 3-ethoxycarbonylpyrrolidine:

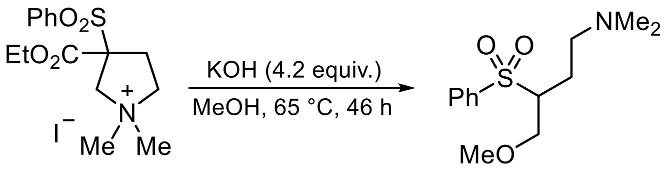

4a (89%)

Corresponding quaternary ammonium salt (0.5 mmol, 1.0 equiv), MeOH (2 mL) and solution of KOH (118 mg, 2.1 mmol, 4.2 equiv) in MeOH (2 mL) were sequentially added to the 10 mL pressure microwave vial equipped with magnetic stirrer. Vial was sealed with cap and heated in an oil bath at 65 °C for 46 h under constant stirring. Followed by the usual **work-up**. The product **4a** was isolated in 89% yield and 77% overall yield. NMR data was in match with the product **4a** obtained via **general procedure**.

3. Analytical data of products

(1-Methyl-3-(phenylsulfonyl)pyrrolidin-3-yl)(phenyl)methanone (2a)

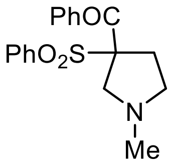

Synthesized from 1-phenyl-2-(phenylsulfonyl)ethan-1-one according to the **general procedure** and isolated by an **acid-base work-up**.

Brown viscous oil. Yield: 1276 mg (97%).

^{1}H NMR (400 MHz, $CDCl_3$) δ 8.04 (d, J = 8.6 Hz, 2H, COPhH-2,6), 7.73 (d, J = 8.5 Hz, 2H, SO_2PhH-2,6), 7.66 (t, J = 7.5 Hz, 1H), 7.57 (t, J = 7.4 Hz, 1H), 7.51 (t, J = 7.8 Hz, 2H), 7.46 (t, J = 7.6 Hz, 2H), 3.45 (d, J = 10.7 Hz, 1H, 2-C*H*H), 3.25 (d, J = 10.7 Hz, 1H, 2-CH*H*), 3.04–2.94 (m, 1H), 2.77–2.65 (m, 3H), 2.31 (s, 3H, NMe).

^{13}C NMR (126 MHz, $CDCl_3$) δ 196.1 (C=O), 136.0 (C), 135.6 (C), 134.4 (CH), 133.1 (CH), 130.5 (2CH), 130.3 (2CH), 128.9 (2CH), 128.3 (2CH), 83.0 (3-C), 62.1 (CH_2N), 56.1 (NCH_2), 41.7 (NMe), 33.4 (CH_2).

HRMS (ESI) calcd for $(C_{18}H_{20}NO_3S)^+$ $[M+H]^+$: 330.1158, found: 330.1170.

(1-Methyl-3-tosylpyrrolidin-3-yl)(phenyl)methanone (2b)

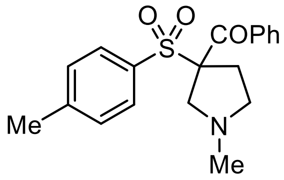

Synthesized from 1-phenyl-2-tosylethan-1-one according to the **general procedure** and isolated by an **acid-base work-up**.

Light brown solid, mp 125–127 °C. Yield: 1139 mg (83%).

^{1}H NMR (500 MHz, $CDCl_3$) δ 8.05 (d, J = 8.4 Hz, 2H, SO_2ArH-2,6), 7.60 (d, J = 8.4 Hz, 2H, SO_2ArH-3,5), 7.56 (t, J = 7.4 Hz, 1H, PhH-4), 7.46 (t, J = 7.7 Hz, 2H, PhH-3,5), 7.30 (d, J = 8.1 Hz, 2H, COPhH-2,6), 3.42 (d, J = 10.6 Hz, 1H, 2-C*H*H), 3.21 (d, J = 10.6 Hz, 1H, 2-CH*H*), 3.00–2.95 (m, 1H), 2.73–2.62 (m, 3H), 2.44 (s, 3H), 2.29 (s, 3H).

^{13}C NMR (126 MHz, $CDCl_3$) δ 196.4 (C=O), 145.5 (C), 135.7 (C), 133.0 (CH), 130.5 (2CH), 130.3 (2CH), 129.5 (2CH), 128.3 (2CH), 83.0 (3-C), 62.2 (CH_2N), 56.2 (NCH_2), 41.7 (NMe), 33.4 (CH_2), 21.8 (ArMe). One C is masked.

HRMS (ESI) calcd for $(C_{19}H_{22}NO_3S)^+$ $[M+H]^+$: 344.1315, found: 344.1320.

(3-((4-Methoxyphenyl)sulfonyl)-1-methylpyrrolidin-3-yl)(phenyl)methanone (2c)

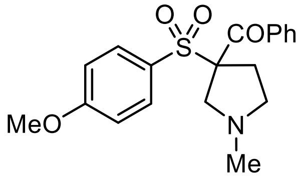

Synthesized from 2-((4-methoxyphenyl)sulfonyl)-1-phenylethan-1-one according to the **general procedure** and isolated by an **acid-base work-up**.

Light yellow solid, mp 82–83 °C. Yield: 1364 mg (95%).

^{1}H NMR (500 MHz, $CDCl_3$) δ 8.05 (d, J = 8.6 Hz, 2H, COPhH-2,6), 7.64 (d, J = 9.0 Hz, 2H, SO_2ArH-2,6), 7.55 (t, J = 7.4 Hz, 1H, COPhH-4), 7.45 (t, J = 7.7 Hz, 2H, COPhH-3,5), 6.95 (d, J = 9.0 Hz, 2H, SO_2ArH-3,5), 3.87 (s, 3H, OMe), 3.42 (d, J = 10.6 Hz, 1H, 2-C*H*H), 3.20 (d, J = 10.6 Hz, 1H, 2-CH*H*), 3.00–2.93 (m, 1H), 2.73–2.61 (m, 3H), 2.29 (s, 3H, NMe).

^{13}C NMR (126 MHz, $CDCl_3$) δ 196.6 (C=O), 164.3 (C-O), 135.8 (C), 133.0 (CH), 132.5 (2CH), 130.5 (2CH), 128.3 (2CH), 127.3 (C), 114.1 (2CH), 83.1 (3-C), 62.3 (CH_2N), 56.2 (NCH_2), 55.8 (OMe), 41.8 (NMe), 33.5 (CH_2).

HRMS (ESI) calcd for $(C_{19}H_{22}NO_4S)^+$ $[M+H]^+$: 360.1264, found: 360.1275.

(3-((3,4-Dimethoxyphenyl)sulfonyl)-1-methylpyrrolidin-3-yl)(phenyl)methanone (2d)

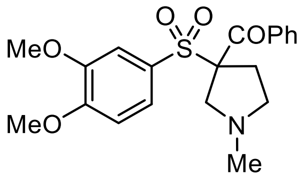

Synthesized from 2-((3,4-dimethoxyphenyl)sulfonyl)-1-phenylethan-1-one according to the **general procedure** and isolated by an **acid-base work-up**.
Light yellow amorphous solid. Yield: 1354 mg (87%).
^{1}H NMR (500 MHz, $CDCl_3$) δ 8.05 (d, *J* = 8.6 Hz, 2H, COPhH-2,6), 7.56 (t, *J* = 7.4 Hz, 1H, COPhH-4), 7.46 (t, *J* = 7.7 Hz, 2H, COPhH-3,5), 7.36 (dd, *J* = 8.5, 2.1 Hz, 1H, SO_2ArH-6), 7.10 (d, *J* = 2.1 Hz, 1H, SO_2ArH-2), 6.92 (d, *J* = 8.5 Hz, 1H, SO_2ArH-5), 3.95 (s, 3H, OMe), 3.79 (s, 3H, OMe), 3.41 (d, *J* = 10.7 Hz, 1H, 2-C*H*H), 3.25 (d, *J* = 10.7 Hz, 1H, 2-CH*H*), 2.99–2.93 (m, 1H), 2.74–2.64 (m, 3H), 2.30 (s, 3H, NMe).
^{13}C NMR (126 MHz, $CDCl_3$) δ 196.0 (C=O), 154.0 (C-O), 148.9 (C-O), 135.6 (C), 133.0 (CH), 130.6 (2CH), 128.3 (2CH), 127.6 (C), 124.6 (CH), 112.5 (CH), 110.4 (CH), 83.3 (3-C), 62.2 (CH_2N), 56.3, 56.1, 41.8 (NMe), 33.7 (CH_2).
HRMS (ESI) calcd for $(C_{20}H_{24}NO_5S)^+$ $[M+H]^+$: 390.1370, found: 390.1373.

(1-Methyl-3-(methylsulfonyl)pyrrolidin-3-yl)(phenyl)methanone (2e)

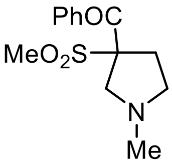

Synthesized from 2-(methylsulfonyl)-1-phenylethan-1-one according to the **general procedure** and isolated by an **acid-base work-up**.
Yellow solid, mp 74–76 °C. Yield: 961 mg (90%).
^{1}H NMR (500 MHz, $CDCl_3$) δ 7.99 (d, *J* = 8.6 Hz, 2H, COPhH-2,6), 7.55 (t, *J* = 7.4 Hz, 1H, COPhH-4), 7.45 (t, *J* = 7.8 Hz, 2H, COPhH-3,5), 3.65 (d, *J* = 10.5 Hz, 1H, 2-C*H*H), 3.21 (d, *J* = 10.6 Hz, 1H, 2-CH*H*), 2.98 (s, 3H, SO_2Me), 2.89–2.83 (m, 1H), 2.79–2.72 (m, 2H), 2.69–2.62 (m, 1H), 2.34 (s, 3H, NMe).
^{13}C NMR (126 MHz, $CDCl_3$) δ 198.2 (C=O), 135.7 (C), 133.1 (CH), 130.1 (2CH), 128.4 (2CH), 81.6 (3-C), 62.0 (CH_2N), 56.1 (NCH_2), 41.6 (NMe), 37.8 (SO_2Me), 32.9 (CH_2).
HRMS (ESI) calcd for $(C_{13}H_{18}NO_3S)^+$ $[M+H]^+$: 268.1002, found: 268.1001.

Ethyl 1-methyl-3-(phenylsulfonyl)pyrrolidine-3-carboxylate (2f)

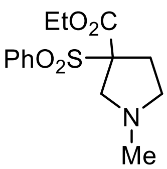

Synthesized from ethyl 2-(phenylsulfonyl)acetate according to the **general procedure** and isolated by an **acid-base work-up**.
Light yellow oil. Yield: 1033 mg (87%).
^{1}H NMR (400 MHz, $CDCl_3$) δ 7.85 (d, *J* = 8.5 Hz, 2H, SO_2PhH-2,6), 7.68 (t, *J* = 7.5 Hz, 1H, SO_2PhH-4), 7.55 (t, *J* = 7.8 Hz, 2H, SO_2PhH-3,5), 4.11 (q, *J* = 7.1 Hz, 2H, $CO_2CH_2CH_3$), 3.34 (d, *J* = 10.3 Hz, 1H, 2-C*H*H), 3.04 (d, *J* = 10.3 Hz, 1H, 2-CH*H*), 2.86 (td, *J* = 7.9, 3.8 Hz, 1H), 2.77–2.61 (m, 2H), 2.58–2.51 (m, 2H), 2.35 (s, 3H, NMe), 1.17 (t, *J* = 7.1 Hz, 3H, $CO_2CH_2CH_3$).
^{13}C NMR (126 MHz, $CDCl_3$) δ 168.0 (CO_2Et), 137.0 (C), 134.3 (CH), 130.0 (2CH), 129.0 (2CH), 78.4 (3-C), 62.9, 60.6, 55.8 (CH_2N), 41.7 (NMe), 30.9 (CH_2), 13.8 ($COOCH_2CH_3$).

HRMS (ESI) calcd for $(C_{14}H_{20}NO_4S)^+$ $[M+H]^+$: 298.1108, found: 298.1117.

4-Methoxy-*N*,*N*-dimethyl-3-(phenylsulfonyl)butan-1-amine (4a)

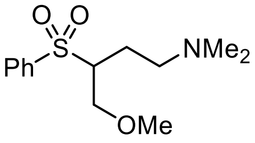

Synthesized from 1-phenyl-2-(phenylsulfonyl)ethan-1-one, applying methyl iodide as alkylating agent and methanol as nucleophile. Product was purified by an **acid-base work-up**.

Light yellow oil. Yield: 112 mg (83%).

^{1}H NMR (500 MHz, $CDCl_3$) δ 7.90 (dd, *J* = 8.6, 1.3 Hz, 2H, PhH-2,6), 7.64 (t, *J* = 7.4 Hz, 1H, PhH-4), 7.55 (t, *J* = 7.7 Hz, 2H, PhH-3,5), 3.69 (d, *J* = 4.8 Hz, 2H, CH_2OMe), 3.45–3.40 (m, 1H, C*H*SO_2Ph), 3.21 (s, 3H, OMe), 2.41 (dt, *J* = 12.2, 7.7 Hz, 1H, C*H*HNMe_2), 2.33 (ddd, *J* = 12.2, 7.3, 5.4 Hz, 1H, CH*H*NMe_2), 2.15 (s, 6H, NMe_2), 2.17–2.08 (m, 1H), 1.84–1.76 (m, 1H).

^{13}C NMR (126 MHz, $CDCl_3$) δ 138.9 (C), 133.6 (CH), 128.99 (2CH), 128.97 (2CH), 69.3 (CH_2OMe), 62.6, 58.9, 56.2, 45.2 (NMe_2), 23.7 (CH_2).

HRMS (ESI) calcd for $(C_{13}H_{22}NO_3S)^+$ $[M+H]^+$: 272.1315, found: 272.1316.

4-Ethoxy-*N*,*N*-dimethyl-3-(phenylsulfonyl)butan-1-amine (4b)

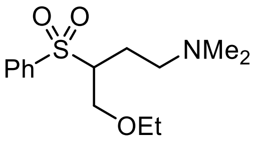

Synthesized from 1-phenyl-2-(phenylsulfonyl)ethan-1-one, applying methyl iodide as alkylating agent and ethanol as nucleophile. Ring-opening reaction was performed in ethanol at 80 °C for 36 h. Product was purified by column chromatography (eluent: chloroform/ethanol, 100/4; R_f (chloroform/ethanol, 100/4) = 0.14).

Light yellow oil. Yield: 106 mg (74%).

^{1}H NMR (400 MHz, $CDCl_3$) δ 7.90 (d, *J* = 8.6 Hz, 2H, PhH-2,6), 7.63 (t, *J* = 7.4 Hz, 1H, PhH-4), 7.53 (t, *J* = 7.6 Hz, 2H, PhH-3,5), 3.73 (dd, *J* = 10.6, 5.2 Hz, 1H, C*H*HOEt), 3.70 (dd, *J* = 10.6, 4.8 Hz, 1H, CH*H*OEt), 3.45–3.40 (m, 1H, C*H*SO_2Ph), 3.34 (q, *J* = 7.0 Hz, 1H, OC*H*HCH_3), 3.33 (q, *J* = 7.0 Hz, 1H, OCH*H*CH_3), 2.41 (dt, *J* = 12.3, 7.6 Hz, 1H, C*H*HNMe_2), 2.34 (ddd, *J* = 12.3, 7.3, 5.4 Hz, 1H, CH*H*NMe_2), 2.16 (s, 6H, NMe_2), 2.20–2.11 (m, 1H), 1.78 (ddd, *J* = 14.1, 7.2, 5.4 Hz, 1H), 0.99 (t, *J* = 7.0 Hz, 3H, OCH_2CH_3).

^{13}C NMR (126 MHz, $CDCl_3$) δ 139.2 (C), 133.5 (CH), 129.0 (2CH), 128.9 (2CH), 67.4, 66.7, 62.7, 56.4, 45.3 (NMe_2), 23.8 (CH_2), 14.9 (OCH_2CH_3).

HRMS (ESI) calcd for $(C_{14}H_{24}NO_3S)^+$ $[M+H]^+$: 286.1471, found: 286.1476.

4-Methoxy-*N*,*N*-dimethyl-3-tosylbutan-1-amine (4c)

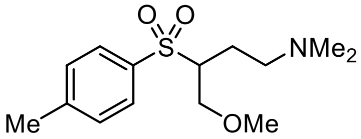

Synthesized from 1-phenyl-2-tosylethan-1-one, applying methyl iodide as alkylating agent and methanol as nucleophile. Product was purified by an **acid-base work-up**.

Light brown oil. Yield: 98 mg (69%).

^{1}H NMR (500 MHz, $CDCl_3$) δ 7.77 (d, *J* = 8.1 Hz, 2H, PhH-2,6), 7.34 (d, *J* = 8.1 Hz, 2H, PhH-3,5), 3.68 (dd, *J* = 10.5, 4.9 Hz, 1H, C*H*HOMe), 3.65 (dd, *J* = 10.5, 4.9 Hz, 1H, CH*H*OMe), 3.42–3.36 (m, 1H,

$CHSO_2Tol$), 3.22 (s, 3H, OMe), 2.44 (s, 3H, $ArCH_3$), 2.50–2.37 (m, 2H), 2.20 (s, 6H, NMe_2), 2.16–2.09 (m, 1H), 1.83 (ddd, *J* = 13.8, 7.8, 5.4 Hz, 1H).

^{13}C NMR (126 MHz, $CDCl_3$) δ 144.7, 135.8, 129.7 (2CH), 129.0 (2CH), 69.4 (CH_2OMe), 62.5, 59.0, 56.2, 45.1 (NMe_2), 23.7 (CH_2), 21.7 (CH_3).

HRMS (ESI) calcd for $(C_{14}H_{24}NO_3S)^+$ $[M+H]^+$: 286.1471, found: 286.1469.

4-Methoxy-3-((4-methoxyphenyl)sulfonyl)-*N,N*-dimethylbutan-1-amine (4d)

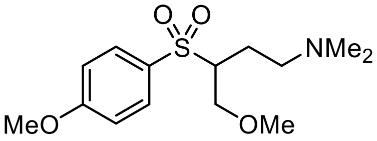

Synthesized from 2-((4-methoxyphenyl)sulfonyl)-1-phenylethan-1-one, applying methyl iodide as alkylating agent and methanol as nucleophile. Product was purified by an **acid-base work-up**.

Light yellow oil. Yield: 81 mg (54%).

^{1}H NMR (400 MHz, $CDCl_3$) δ 7.81 (d, *J* = 9.0 Hz, 2H, ArH-2,6), 7.00 (d, *J* = 9.0 Hz, 2H, ArH-3,5), 3.88 (s, 3H, OMe), 3.68 (d, *J* = 4.8 Hz, 2H, CH_2OMe), 3.39–3.33 (m, 1H, $CHSO_2Ar$), 3.23 (s, 3H, CH_2O*Me*), 2.40 (dt, *J* = 12.2, 7.6 Hz, 1H, C*H*HNMe_2), 2.33 (dt, *J* = 12.2, 6.3 Hz, 1H, CH*H*NMe_2), 2.15 (s, 6H, NMe_2), 2.14–2.04 (m, 1H), 1.77 (dddd, *J* = 12.6, 8.5, 7.3, 5.3 Hz, 1H).

^{13}C NMR (126 MHz, $CDCl_3$) δ 163.8 (C), 131.2 (2CH), 130.3 (C), 114.3 (2CH), 69.5 (CH_2OMe), 62.7, 59.0, 56.3, 55.8, 45.2 (NMe_2), 23.8 (CH_2).

HRMS (ESI) calcd for $(C_{14}H_{24}NO_4S)^+$ $[M+H]^+$: 302.1421, found: 302.1428.

3-((3,4-Dimethoxyphenyl)sulfonyl)-4-methoxy-*N,N*-dimethylbutan-1-amine (4e)

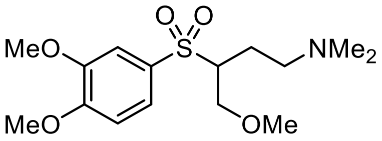

Synthesized from 2-((3,4-dimethoxyphenyl)sulfonyl)-1-phenylethan-1-one, applying methyl iodide as alkylating agent and methanol as nucleophile. Product was purified by an **acid-base work-up**.

Light yellow viscous oil. Yield: 93 mg (56%).

^{1}H NMR (500 MHz, $CDCl_3$) δ 7.51 (dd, *J* = 8.5, 2.1 Hz, 1H, ArH-6), 7.34 (d, *J* = 2.1 Hz, 1H, ArH-2), 6.97 (d, *J* = 8.5 Hz, 1H, ArH-5), 3.96 (s, 3H, OMe), 3.94 (s, 3H, OMe), 3.69 (d, *J* = 4.8 Hz, 2H, CH_2OMe), 3.41–3.36 (m, 1H, $CHSO_2Ar$), 3.26 (s, 3H, OMe), 2.40 (dt, *J* = 12.3, 7.6 Hz, 1H, C*H*HNMe_2), 2.32 (ddd, *J* = 12.3, 7.4, 5.3 Hz, 1H, CH*H*NMe_2), 2.16 (s, 6H, NMe_2), 2.13–2.06 (m, 1H), 1.80 (dddd, *J* = 12.5, 8.4, 7.3, 5.3 Hz, 1H).

^{13}C NMR (126 MHz, $CDCl_3$) δ 153.4 (C), 149.1 (C), 130.3 (C), 123.1 (CH), 111.2 (CH), 110.6 (CH), 69.4 (CH_2OMe), 62.8, 59.0, 56.4 (OMe), 56.33 (OMe), 56.29 (OMe), 45.3 (NMe_2), 23.9 (CH_2).

HRMS (ESI) calcd for $(C_{15}H_{26}NO_5S)^+$ $[M+H]^+$: 332.1526, found: 332.1524.

4-Methoxy-*N,N*-dimethyl-3-((3-nitrophenyl)sulfonyl)butan-1-amine (4f)

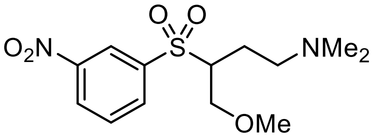

Synthesized from 2-((3-nitrophenyl)sulfonyl)-1-phenylethan-1-one, applying methyl iodide as alkylating agent and methanol as nucleophile. Product was purified by an **acid-base work-up** and column chromatography (eluent: chloroform/ethanol, 100/4; R_f (chloroform/ethanol, 100/4) = 0.1).

Brown oil. Yield: 33 mg (21%).

^{1}H NMR (400 MHz, $CDCl_3$) δ 8.76 (t, *J* = 1.8 Hz, 1H, ArH), 8.48 (ddd, *J* = 8.3, 2.2, 1.0 Hz, 1H, ArH), 8.22 (ddd, *J* = 7.8, 1.7, 1.1 Hz, 1H, ArH), 7.74 (t, *J* = 8.0 Hz, 1H, ArH), 3.82 (dd, *J* = 10.6, 3.3 Hz, 1H, C*H*HOMe), 3.68 (dd, *J* = 10.6, 5.6 Hz, 1H, CH*H*OMe), 3.55–3.49 (m, 1H, $CHSO_2Ph$), 3.18 (s, 3H, OMe), 2.46–2.30 (m, 2H), 2.23–2.14 (m, 1H), 2.19 (s, 6H, NMe_2), 1.74–1.64 (m, 1H).

^{13}C NMR (126 MHz, $CDCl_3$) δ 148.2 (C), 141.8 (C), 134.7 (CH), 130.0 (CH), 128.0 (CH), 124.7 (CH), 69.0 (*CH_2*OMe), 63.4, 58.8, 55.9, 45.3 (NMe_2), 23.2 (CH_2).

HRMS (ESI) calcd for $(C_{13}H_{21}N_2O_5S)^+$ $[M+H]^+$: 317.1166, found: 317.1165.

N^1,N^1,N^4,N^4-tetramethyl-2-((3-nitrophenyl)sulfonyl)butane-1,4-diamine (4g)

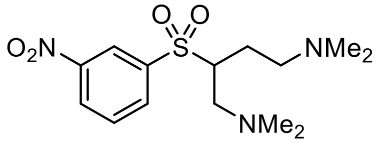

Synthesized from 2-((3-nitrophenyl)sulfonyl)-1-phenylethan-1-one, applying methyl iodide as alkylating agent and 33% aqueous solution of dimethylamine as nucleophile (5.0 equiv, 2.5 mmol of $NHMe_2$). Product was purified by an **acid-base work-up** and column chromatography (eluent: chloroform/ethanol, 100/12; R*f* (chloroform/ethanol, 100/12) = 0.15).

Brown oil. Yield: 40 mg (24%).

^{1}H NMR (400 MHz, $CDCl_3$) δ 8.78 (t, *J* = 1.8 Hz, 1H, ArH), 8.46 (ddd, *J* = 8.2, 2.2, 1.0 Hz, 1H, ArH), 8.23 (ddd, *J* = 7.8, 1.6, 1.0 Hz, 1H, ArH), 7.74 (t, *J* = 8.0 Hz, 1H, ArH), 3.52 (ddd, *J* = 12.3, 7.6, 4.8 Hz, 1H, $CHSO_2Ph$), 2.76 (dd, *J* = 13.2, 7.6 Hz, 1H), 2.45–2.41 (m, 2H), 2.38 (dd, *J* = 13.2, 5.9 Hz, 1H), 2.31–2.21 (m, 1H), 2.19 (s, 6H, NMe_2), 1.98 (s, 6H, N′Me_2), 1.71 (dt, *J* = 13.5, 6.2 Hz, 1H).

^{13}C NMR (126 MHz, $CDCl_3$) δ 148.1 (C), 142.5 (C), 134.3 (CH), 130.0 (CH), 127.7 (CH), 124.4 (CH), 60.4, 58.9, 56.2, 45.3 (NMe_2), 45.0 (N′Me_2), 24.8 (CH_2).

HRMS (ESI) calcd for $(C_{14}H_{24}N_3O_4S)^+$ $[M+H]^+$: 330.1482, found: 330.1487.

***N*-Ethyl-4-methoxy-*N*-methyl-3-(methylsulfonyl)butan-1-amine (4h)**

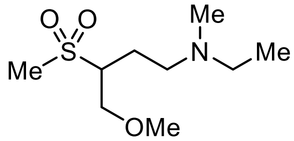

Synthesized from 2-(methylsulfonyl)-1-phenylethan-1-one, applying ethyl iodide as alkylating agent and methanol as nucleophile. Product was purified by an **acid-base work-up**.

Light yellow oil. Yield: 45 mg (40%).

^{1}H NMR (500 MHz, $CDCl_3$) δ 3.83 (dd, *J* = 10.5, 2.8 Hz, 1H, C*H*HOMe), 3.70 (dd, *J* = 10.5, 6.3 Hz, 1H, CH*H*OMe), 3.37 (s, 3H, OMe), 3.22–3.17 (m, 1H, C*H*SO_2Me), 2.93 (s, 3H, SO_2Me), 2.54–2.36 (m, 4H), 2.28–2.18 (m, 1H), 2.21 (s, 3H, NMe), 1.76 (dddd, *J* = 14.3, 9.5, 6.8, 5.1 Hz, 1H), 1.04 (t, *J* = 7.1 Hz, 3H, NCH_2CH_3).

^{13}C NMR (126 MHz, $CDCl_3$) δ 69.6 (*CH_2*OMe), 62.2, 59.2, 53.7, 51.3, 41.33, 41.25, 22.1 (CH_2), 12.2 (CH_3).

HRMS (ESI) calcd for $(C_9H_{22}NO_3S)^+$ $[M+H]^+$: 224.1315, found: 224.1323.

N-Benzyl-4-methoxy-N-methyl-3-(methylsulfonyl)butan-1-amine (4i)

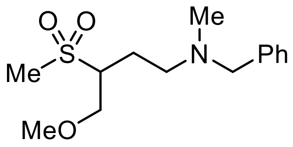

Synthesized from 2-(methylsulfonyl)-1-phenylethan-1-one, applying benzyl chloride as alkylating agent (reaction was performed at 70 °C for 7 days) and methanol as nucleophile. Product was purified by an **acid-base work-up**.

Yellow oil. Yield: 95 mg (67%).

^{1}H NMR (400 MHz, $CDCl_3$) δ 7.34–7.23 (m, 5H, PhH), 3.69 (dd, *J* = 10.6, 2.6 Hz, 1H, C*H*HOMe), 3.60 (d, *J* = 13.0 Hz, 1H, NC*H*HPh), 3.47 (dd, *J* = 10.6, 6.2 Hz, 1H, CH*H*OMe), 3.39 (d, *J* = 13.0 Hz, 1H, NCH*H*Ph), 3.26 (s, 3H, OMe), 3.28–3.21 (m, 1H, C*H*SO_2Me), 2.90 (d, *J* = 0.5 Hz, 3H, SO_2Me), 2.53 (ddd, *J* = 12.5, 9.5, 5.8 Hz, 1H, C*H*HNMeBn), 2.41 (ddd, *J* = 12.5, 6.2, 4.6 Hz, 1H, CH*H*NMeBn), 2.28 (ddd, *J* = 14.0, 6.0, 3.4 Hz, 1H), 2.24 (s, 3H, NMe), 1.78–1.68 (m, 1H).

^{13}C NMR (126 MHz, $CDCl_3$) δ 139.1 (C), 129.2 (2CH), 128.4 (2CH), 127.3 (CH), 69.0 (*CH_2*OMe), 62.6, 62.0, 59.1, 53.1, 42.0, 41.3, 22.0 (CH_2).

HRMS (ESI) calcd for $(C_{14}H_{24}NO_3S)^+$ $[M+H]^+$: 286.1471, found: 286.1471.

N-Allyl-4-methoxy-N-methyl-3-(phenylsulfonyl)butan-1-amine (4j)

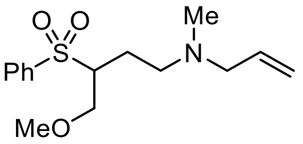

Synthesized from 1-phenyl-2-(phenylsulfonyl)ethan-1-one, applying allyl bromide as alkylating agent and methanol as nucleophile. Product was purified by an **acid-base work-up**.

Yellow oil. Yield: 101 mg (68%).

^{1}H NMR (400 MHz, $CDCl_3$) δ 7.90 (d, *J* = 7.6 Hz, 1H, PhH-2,6), 7.64 (t, *J* = 7.4 Hz, 1H, PhH-4), 7.55 (t, *J* = 7.6 Hz, 1H, PhH-3,5), 5.76 (ddt, *J* = 16.9, 10.2, 6.5 Hz, 1H, NCH_2C*H*=), 5.16–5.08 (m, 2H, NCH_2CH=C*H_2*), 3.71 (dd, *J* = 9.3, 3.4 Hz, 1H, C*H*HOMe), 3.67 (dd, *J* = 9.3, 3.4 Hz, 1H, CH*H*OMe), 3.46–3.39 (m, 1H), 3.21 (s, 3H, OMe), 2.96 (dd, *J* = 13.6, 6.3 Hz, 1H, NC*H*HCH=), 2.89 (dd, *J* = 13.6, 6.8 Hz, 1H, NCH*H*CH=), 2.47 (ddd, *J* = 12.4, 7.9, 7.0 Hz, 1H, C*H*HNMeAllyl), 2.39 (ddd, *J* = 12.4, 7.2, 5.2 Hz, 1H, CH*H*NMeAllyl), 2.17–2.08 (m, 1H), 2.12 (s, 3H, NMe), 1.78 (dddd, *J* = 14.1, 8.6, 7.0, 5.2 Hz, 1H).

^{13}C NMR (126 MHz, $CDCl_3$) δ 139.0 (C), 135.6 (CH), 133.6 (CH), 129.0 (2CH), 117.6 (CH_2=), 69.2 (*CH_2*OMe), 62.6, 60.8, 58.9, 53.6, 41.7, 23.4 (CH_2).

HRMS (ESI) calcd for $(C_{15}H_{24}NO_3S)^+$ $[M+H]^+$: 298.1471, found: 298.1466.

N-(2-(1,3-Dioxan-2-yl)ethyl)-4-methoxy-N-methyl-3-(methylsulfonyl)butan-1-amine (4k)

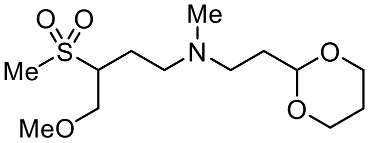

Synthesized from 2-(methylsulfonyl)-1-phenylethan-1-one, applying 2-(2-bromoethyl)-1,3-dioxane as alkylating agent (reaction was performed at 70 °C for 7 days) and methanol as nucleophile.

Brown viscous oil. Yield: 91 mg (59%).

^{1}H NMR (400 MHz, $CDCl_3$) δ 4.59 (t, *J* = 5.2 Hz, 1H, OC*H*O), 4.09 (ddd, *J* = 6.4, 5.4, 1.6 Hz, 2H), 3.84 (dd, *J* = 10.6, 2.6 Hz, 1H, C*H*HOMe), 3.75 (tt, *J* = 11.9, 2.6 Hz, 2H), 3.68 (dd, *J* = 10.6, 6.1 Hz, 1H, CH*H*OMe), 3.37 (s, 3H, OMe), 3.25–3.18 (m, 1H), 2.92 (s, 3H, $MeSO_2$), 2.53–2.39 (m, 4H), 2.26–2.17 (m, 1H), 2.20 (s, 3H, NMe), 2.07 (qt, *J* = 13.3, 5.0 Hz, 1H), 1.80–1.70 (m, 3H), 1.33 (dddd, *J* = 13.5, 3.8, 2.5,

1.3 Hz, 1H).

^{13}C NMR (126 MHz, $CDCl_3$) δ 101.0 (O*C*HO), 69.3 (*CH*$_2$OMe), 67.0 (2OCH_2), 62.0, 59.2, 54.2, 52.3, 41.6, 41.3, 33.0, 25.9, 22.2.

HRMS (ESI) calcd for $(C_{13}H_{28}NO_5S)^+$ $[M+H]^+$: 310.1683, found: 310.1685.

N^1,N^1,N^4,N^4-Tetramethyl-2-(phenylsulfonyl)butane-1,4-diamine (4l)

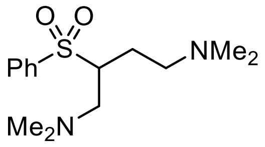

Synthesized from 1-phenyl-2-(phenylsulfonyl)ethan-1-one, applying methyl iodide as alkylating agent and 33% aqueous solution of dimethylamine as nucleophile (5.0 equiv, 2.5 mmol of $NHMe_2$).

Brown oil. Yield: 138 mg (97%).

^{1}H NMR (500 MHz, $CDCl_3$) δ 7.90 (d, J = 8.6 Hz, 2H, PhH-2,6), 7.63 (t, J = 7.4 Hz, 1H, PhH-4), 7.55 (t, J = 7.7 Hz, 2H, PhH-3,5), 3.39 (dq, J = 8.4, 5.3 Hz, 1H, $CHSO_2Ph$), 2.65 (dd, J = 12.8, 5.0 Hz, 1H), 2.41 (dd, J = 12.8, 8.4 Hz, 1H), 2.40–2.35 (m, 2H), 2.14 (s, 6H, NMe_2), 2.09 (s, 6H, N′Me_2), 2.12–2.05 (m, 1H), 1.80 (td, J = 13.6, 6.5 Hz, 1H).

^{13}C NMR (126 MHz, $CDCl_3$) δ 139.0 (C), 133.5 (CH), 129.0 (2CH), 128.9 (2CH), 60.3, 58.7, 56.6, 45.5 (NMe_2), 45.3 (N′Me_2), 25.8 (CH_2).

HRMS (ESI) calcd for $(C_{14}H_{25}N_2O_2S)^+$ $[M+H]^+$: 285.1631, found: 285.1633.

N^1,N^1-Diethyl-N^4,N^4-dimethyl-2-(phenylsulfonyl)butane-1,4-diamine (4m)

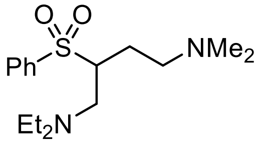

Synthesized from 1-phenyl-2-(phenylsulfonyl)ethan-1-one, applying methyl iodide as alkylating agent and diethylamine (4.0 equiv, 2.0 mmol) as nucleophile.

Brown oil. Yield: 117 mg (75%).

^{1}H NMR (400 MHz, $CDCl_3$) δ 7.91–7.89 (m, 2H, PhH-2,6), 7.63 (tt, J = 7.4, 1.3 Hz, 1H, PhH-4), 7.55 (t, J = 7.5 Hz, 2H, PhH-3,5), 3.37 (dq, J = 8.8, 5.5 Hz, 1H, $CHSO_2Ph$), 2.81 (dd, J = 13.2, 4.6 Hz, 1H, C*H*HNEt$_2$), 2.49 (dd, J = 13.2, 8.8 Hz, 1H, CH*H*NEt$_2$), 2.47–2.26 (m, 6H), 2.15 (s, 6H, NMe_2), 2.16–2.06 (m, 1H), 1.90–1.80 (m, 1H), 0.84 (t, J = 7.1 Hz, 6H, N(CH$_2$C*H*$_3$)$_2$).

^{13}C NMR (126 MHz, $CDCl_3$) δ 139.3 (C), 133.5 (CH), 129.0 (2CH), 128.8 (2CH), 60.3, 56.9, 53.0, 46.8 (2C), 45.3 (2C), 25.5 (CH_2), 11.4 (N(CH$_2$*CH*$_3$)$_2$).

HRMS (ESI) calcd for $(C_{16}H_{29}N_2O_2S)^+$ $[M+H]^+$: 313.1944, found: 313.1945.

***N*,*N*-Dimethyl-3-(phenylsulfonyl)-4-(pyrrolidin-1-yl)butan-1-amine (4n)**

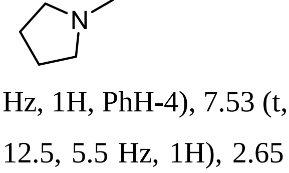

Synthesized from 1-phenyl-2-(phenylsulfonyl)ethan-1-one, applying methyl iodide as alkylating agent and pyrrolidine (4.0 equiv, 2.0 mmol) as nucleophile.

Brown oil. Yield: 141 mg (91%).

^{1}H NMR (500 MHz, $CDCl_3$) δ 7.91–7.89 (m, 2H, PhH-2,6), 7.62 (tt, J = 7.4, 1.2 Hz, 1H, PhH-4), 7.53 (t, J = 7.7 Hz, 2H, PhH-3,5), 3.42 (dq, J = 8.2, 5.5 Hz, 1H, $CHSO_2Ph$), 2.81 (dd, J = 12.5, 5.5 Hz, 1H), 2.65 (dd, J = 12.5, 8.2 Hz, 1H), 2.42–2.36 (m, 4H), 2.29–2.24 (m, 2H), 2.15 (s, 6H, NMe_2), 2.18–2.09 (m, 1H), 1.87–1.79 (m, 1H), 1.61–1.55 (m, 4H).

^{13}C NMR (126 MHz, $CDCl_3$) δ 139.4 (C), 133.4 (CH), 128.9 (2CH), 128.8 (2CH), 61.0, 56.8, 55.1, 53.9 (2 NCH_2), 45.3 (NMe_2), 25.4 (CH_2), 23.7 ($2CH_2$).

HRMS (ESI) calcd for $(C_{16}H_{27}N_2O_2S)^+$ $[M+H]^+$: 311.1788, found: 311.1791.

_N,N_-Dimethyl-4-morpholino-3-(phenylsulfonyl)butan-1-amine (4o)

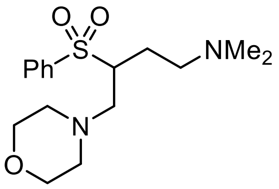

Synthesized from 1-phenyl-2-(phenylsulfonyl)ethan-1-one, applying methyl iodide as alkylating agent and morpholine (4.0 equiv, 2.0 mmol) as nucleophile. Brown oil. Yield: 153 mg (94%).

^{1}H NMR (500 MHz, $CDCl_3$) δ 7.90 (d, J = 8.5 Hz, 2H, PhH-2,6), 7.64 (t, J = 7.4 Hz, 1H, PhH-4), 7.55 (t, J = 7.7 Hz, 2H, PhH-3,5), 3.53–3.47 (m, 1H, $CHSO_2Ph$), 3.47–3.39 (m, 4H, CH_2OCH_2), 2.77 (dd, J = 13.1, 6.1 Hz, 1H), 2.46–2.36 (m, 2H), 2.43 (dd, J = 13.1, 7.3 Hz, 1H), 2.34–2.17 (m, 5H), 2.16 (s, 6H, NMe_2), 1.76 (td, J = 13.0, 6.5 Hz, 1H).

^{13}C NMR (126 MHz, $CDCl_3$) δ 139.6 (C), 133.5 (CH), 129.0 (2CH), 128.8 (2CH), 66.8 ($\mathit{C}H_2O\mathit{C}H_2$), 58.9, 58.0, 56.4, 53.6 ($\mathit{C}H_2N\mathit{C}H_2$), 45.3 ($NMe_2$), 25.1 ($CH_2$).

HRMS (ESI) calcd for $(C_{16}H_{27}N_2O_3S)^+$ $[M+H]^+$: 327.1737, found: 327.1740.

4-(3,4-Dihydroisoquinolin-2(_1H_)-yl)-_N,N_-dimethyl-3-(phenylsulfonyl)butan-1-amine (4p)

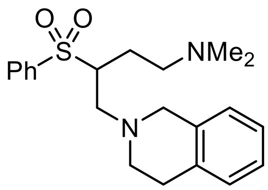

Synthesized from 1-phenyl-2-(phenylsulfonyl)ethan-1-one, applying methyl iodide as alkylating agent and 1,2,3,4-tetrahydroisoquinoline (1.3 equiv, 0.65 mmol) as nucleophile. Product was purified by column chromatography (eluent: chloroform/ethanol, from 100/0 to 100/3; R_f (chloroform/ethanol, 100/3) = 0.22). Brown amorphous solid. Yield: 125 mg (67%).

^{1}H NMR (500 MHz, $CDCl_3$) δ 7.90 (d, J = 8.5 Hz, 2H, PhH-2,6), 7.55 (t, J = 7.4 Hz, 1H, PhH-4), 7.46 (t, J = 7.7 Hz, 2H, PhH-3,5), 7.11–7.05 (m, 2H), 7.01 (dd, J = 6.2, 2.1 Hz, 1H), 6.92 (dd, J = 6.2, 2.1 Hz, 1H), 3.63–3.58 (m, 1H, $CHSO_2Ph$), 3.56 (d, J = 14.7 Hz, 1H, $^{THIQ}NC\mathit{H}H$), 3.43 (d, J = 14.7 Hz, 1H, $^{THIQ}NCH\mathit{H}$), 2.93 (dd, J = 13.0, 5.5 Hz, 1H), 2.72–2.59 (m, 4H), 2.59–2.53 (m, 1H), 2.45–2.37 (m, 2H), 2.20 (dtd, J = 12.3, 7.1, 5.2 Hz, 1H), 2.13 (s, 6H, NMe_2), 1.89–1.81 (m, 1H).

^{13}C NMR (126 MHz, $CDCl_3$) δ 139.3 (C), 134.5 (C), 134.2 (C), 133.5 (CH), 129.0 (2CH), 128.8 (2CH), 128.6 (CH), 126.5 (CH), 126.2 (CH), 125.7 (CH), 59.7, 57.4, 56.6, 56.1, 50.9, 45.3 (NMe_2), 29.1 (CH_2), 25.4 (CH_2).

HRMS (ESI) calcd for $(C_{21}H_{29}N_2O_2S)^+$ $[M+H]^+$: 373.1944, found: 373.1950.

4-(Butylthio)-_N,N_-dimethyl-3-(phenylsulfonyl)butan-1-amine (4q)

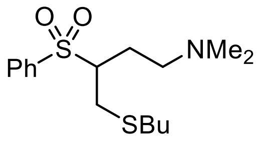

Synthesized from 1-phenyl-2-(phenylsulfonyl)ethan-1-one, applying methyl iodide as alkylating agent and butane-1-thiol (3.0 equiv, 1.5 mmol) as nucleophile. Product was purified by column chromatography (eluent: chloroform/ethanol, 100/5; R_f (chloroform/ethanol, 100/5) = 0.7).

Light orange oil. Yield: 100 mg (61%).

^{1}H NMR (500 MHz, $CDCl_3$) δ 7.91 (d, *J* = 8.5 Hz, 2H, PhH-2,6), 7.67 (t, *J* = 7.4 Hz, 1H, PhH-4), 7.58 (t, *J* = 7.7 Hz, 2H, PhH-3,5), 3.38 (dtd, *J* = 9.4, 5.7, 3.3 Hz, 1H, $CHSO_2Ph$), 3.03 (dd, *J* = 13.6, 3.3 Hz, 1H, C*H*HSBu), 2.56 (dd, *J* = 13.6, 9.9 Hz, 1H, CH*H*SBu), 2.43 (t, *J* = 7.3 Hz, 2H, SC*H₂*Pr), 2.47–2.36 (m, 2H, CH_2NMe_2), 2.16 (s, 6H, NMe_2), 2.10–2.02 (m, 1H), 1.96 (tdd, *J* = 12.6, 7.1, 5.2 Hz, 1H), 1.48–1.41 (m, 2H), 1.37–1.29 (m, 2H), 0.87 (t, *J* = 7.3 Hz, 3H, $S(CH_2)_3CH_3$).

^{13}C NMR (126 MHz, $CDCl_3$) δ 137.9 (C), 133.9 (CH), 129.4 (2CH), 129.0 (2CH), 61.7, 56.5 (CH_2NMe_2), 45.3 (NMe_2), 32.3 (CH_2), 31.3 (CH_2), 30.9 (CH_2), 25.8 (CH_2), 22.0 (CH_2), 13.7 ($S(CH_2)_3CH_3$).

HRMS (ESI) calcd for $(C_{16}H_{28}NO_2S_2)^+$ $[M+H]^+$: 330.1556, found: 330.1570.

_N,N_-Dimethyl-3-(phenylsulfonyl)-4-(phenylthio)butan-1-amine (4r)

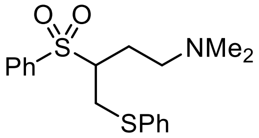

Synthesized from 1-phenyl-2-(phenylsulfonyl)ethan-1-one, applying methyl iodide as alkylating agent and thiophenol (2.0 equiv, 1.0 mmol) as nucleophile. Product was purified by an **acid-base work-up**.

Brown oil. Yield: 145 mg (83%).

^{1}H NMR (500 MHz, $CDCl_3$) δ 7.86 (dd, *J* = 8.3, 1.1 Hz, 2H, SO_2PhH-2,6), 7.67 (t, *J* = 7.5 Hz, 1H, SO_2PhH-4), 7.56 (t, *J* = 7.8 Hz, 2H, SO_2PhH-3,5), 7.24–7.15 (m, 5H, SPhH), 3.48 (dd, *J* = 13.9, 3.2 Hz, 1H, PhSC*H*H), 3.33 (dddd, *J* = 9.7, 6.0, 5.3, 3.2 Hz, 1H, $CHSO_2Ph$), 2.89 (dd, *J* = 13.9, 9.7 Hz, 1H, PhSCH*H*), 2.41–2.34 (m, 2H, CH_2NMe_2), 2.17–2.10 (m, 1H), 2.13 (s, 6H, NMe_2), 2.00 (dtd, *J* = 12.7, 7.2, 5.2 Hz, 1H).

^{13}C NMR (126 MHz, $CDCl_3$) δ 137.8 (C), 134.7 (C), 133.9 (CH), 129.9 (2CH), 129.4 (2CH), 129.3 (2CH), 129.0 (2CH), 126.9 (CH), 61.2, 56.5 (CH_2NMe_2), 45.3 (NMe_2), 33.1, 25.7 (CH_2).

HRMS (ESI) calcd for $(C_{18}H_{24}NO_2S_2)^+$ $[M+H]^+$: 350.1243, found: 350. 1251.

3-((4-Methoxyphenyl)sulfonyl)-_N,N_-dimethyl-4-(phenylthio)butan-1-amine (4s)

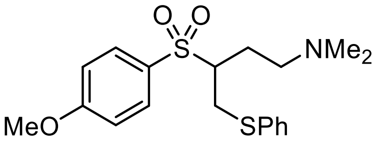

Synthesized from 2-((4-methoxyphenyl)sulfonyl)-1-phenylethan-1-one, applying methyl iodide as alkylating agent and thiophenol (2.0 equiv, 1.0 mmol) as nucleophile. Product was purified by column chromatography (eluent: chloroform/ethanol, from 100/0 to 100/5; R_f (chloroform/ethanol, 100/5) = 0.33).

Yellow viscous oil. Yield: 165 mg (87%).

^{1}H NMR (400 MHz, $CDCl_3$) δ 7.78 (d, *J* = 8.9 Hz, 2H, SO_2ArH-2,6), 7.24–7.17 (m, 5H, SPhH), 7.01 (d, *J* = 8.9 Hz, 2H, SO_2ArH-3,5), 3.90 (s, 3H, OMe), 3.49 (dd, *J* = 13.8, 3.1 Hz, 1H, PhSC*H*H), 3.30–3.23 (m, 1H, $CHSO_2Ar$), 2.87 (dd, *J* = 13.8, 9.9 Hz, 1H, PhSCH*H*), 2.40 (t, *J* = 7.3 Hz, 2H, CH_2NMe_2), 2.15 (s, 6H, NMe_2), 2.16–2.07 (m, 1H), 1.98 (dtd, *J* = 12.5, 7.3, 5.3 Hz, 1H).

^{13}C NMR (126 MHz, $CDCl_3$) δ 164.1 (C-O), 134.9 (C), 131.2 (2CH), 129.8 (2CH), 129.3 (2CH), 129.2 (C), 126.9 (CH), 114.6 (2CH), 61.6 ($CHSO_2$), 56.6, 55.9, 45.3 (NMe_2), 33.2 (SCH_2), 25.8 (CH_2).

HRMS (ESI) calcd for $(C_{19}H_{26}NO_3S_2)^+$ $[M+H]^+$: 380.1349, found: 380.1355.

3-((3,4-Dimethoxyphenyl)sulfonyl)-*N*,*N*-dimethyl-4-(phenylthio)butan-1-amine (4t)

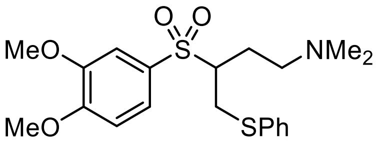

Synthesized from 2-((3,4-dimethoxyphenyl)sulfonyl)-1-phenylethan-1-one, applying methyl iodide as alkylating agent and thiophenol (2.0 equiv, 1.0 mmol) as nucleophile. Product was purified by column chromatography (eluent: chloroform/ethanol, from 100/1 to 100/4; R_f (chloroform/ethanol, 100/4) = 0.21).

Yellow viscous oil. Yield: 135 mg (66%).

^{1}H NMR (500 MHz, $CDCl_3$) δ 7.48 (dd, *J* = 8.4, 2.1 Hz, 1H, SO_2ArH-6), 7.24 (d, *J* = 2.1 Hz, 1H, SO_2ArH-2), 7.23–7.16 (m, 5H, SPhH), 6.97 (d, *J* = 8.4 Hz, 1H, SO_2ArH-5), 3.97 (s, 3H, OMe), 3.89 (s, 3H, OMe), 3.45 (dd, *J* = 13.9, 3.1 Hz, 1H, PhSC*H*H), 3.21–3.25 (m, 1H, $CHSO_2$Ar), 2.89 (dd, *J* = 13.9, 9.8 Hz, 1H, PhSCH*H*), 2.42 (t, *J* = 7.1 Hz, 2H, *CH_2*NMe_2), 2.17 (s, 6H, NMe_2), 2.21–2.12 (m, 1H), 2.01 (dtd, *J* = 12.5, 7.3, 5.4 Hz, 1H).

^{13}C NMR (126 MHz, $CDCl_3$) δ 153.7 (C-O), 149.5 (C-O), 134.8 (C), 130.0 (2CH), 129.23 (C), 129.20 (2CH), 126.9 (CH), 123.2 (CH), 110.9 (CH), 110.8 (CH), 61.6 ($CHSO_2$), 56.5 (NCH_2), 56.41 (OMe), 56.40 (OMe), 45.3 (NMe_2), 33.3 (SCH_2), 25.6 (CH_2).

HRMS (ESI) calcd for $(C_{20}H_{28}NO_4S_2)^+$ $[M+H]^+$: 410.1454, found: 410.1469.

***N*,*N*-Dimethyl-3-(phenylsulfonyl)butan-1-amine (5)**

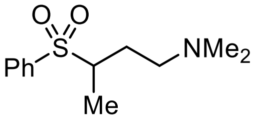

N,*N*-Dimethyl-3-(phenylsulfonyl)-4-(phenylthio)butan-1-amine (**4r**) (175 mg, 0.5 mmol) was dissolved in anhydrous THF (5.0 mL) and $LiAlH_4$ (76 mg, 2.0 mmol, 4.0 equiv) was added in small portions at room temperature. The resulting suspension was stirred at 25 °C for 20 h. Few drops of H_2O/THF mixture (v/v 1/1) was carefully added to the resulting mixture, following by an addition of brine (5 mL) and 40% aqueous NaOH solution (5 mL) and then it was extracted with DCM (2 × 10 mL). Combined DCM extracts were washed with H_2O (2 × 10 mL), brine (10 mL), dried over Na_2SO_4 and evaporated under reduced pressure to give pure product.

Yellow viscous oil. Yield: 93 mg (77%).

^{1}H NMR (500 MHz, $CDCl_3$) δ 7.89 (d, *J* = 8.6 Hz, 2H, SO_2PhH-2,6), 7.65 (t, *J* = 7.5 Hz, 1H, SO_2PhH-4), 7.56 (t, *J* = 7.7 Hz, 2H, SO_2PhH-3,5), 3.24 (dqd, *J* = 10.2, 6.9, 3.9 Hz, 1H, $CHSO_2$Ph), 2.38 (ddd, *J* = 12.0, 8.7, 7.2 Hz, 1H, C*H*HNMe_2), 2.27 (ddd, *J* = 12.0, 7.5, 3.9 Hz, 1H, CH*H*NMe_2), 2.22–2.13 (m, 1H), 2.14 (s, 6H, NMe_2), 1.52–1.45 (m, 1H), 1.27 (d, *J* = 6.9 Hz, 3H, CH_3).

^{13}C NMR (126 MHz, $CDCl_3$) δ 137.5 (C), 133.7 (CH), 129.2 (2CH), 129.1 (2CH), 57.8, 55.9, 45.3 (NMe_2), 27.1 (CH_2), 13.19 (CH_3).

HRMS (ESI) calcd for $(C_{12}H_{20}NO_2S)^+$ $[M+H]^+$: 242.1209, found: 242.1215.

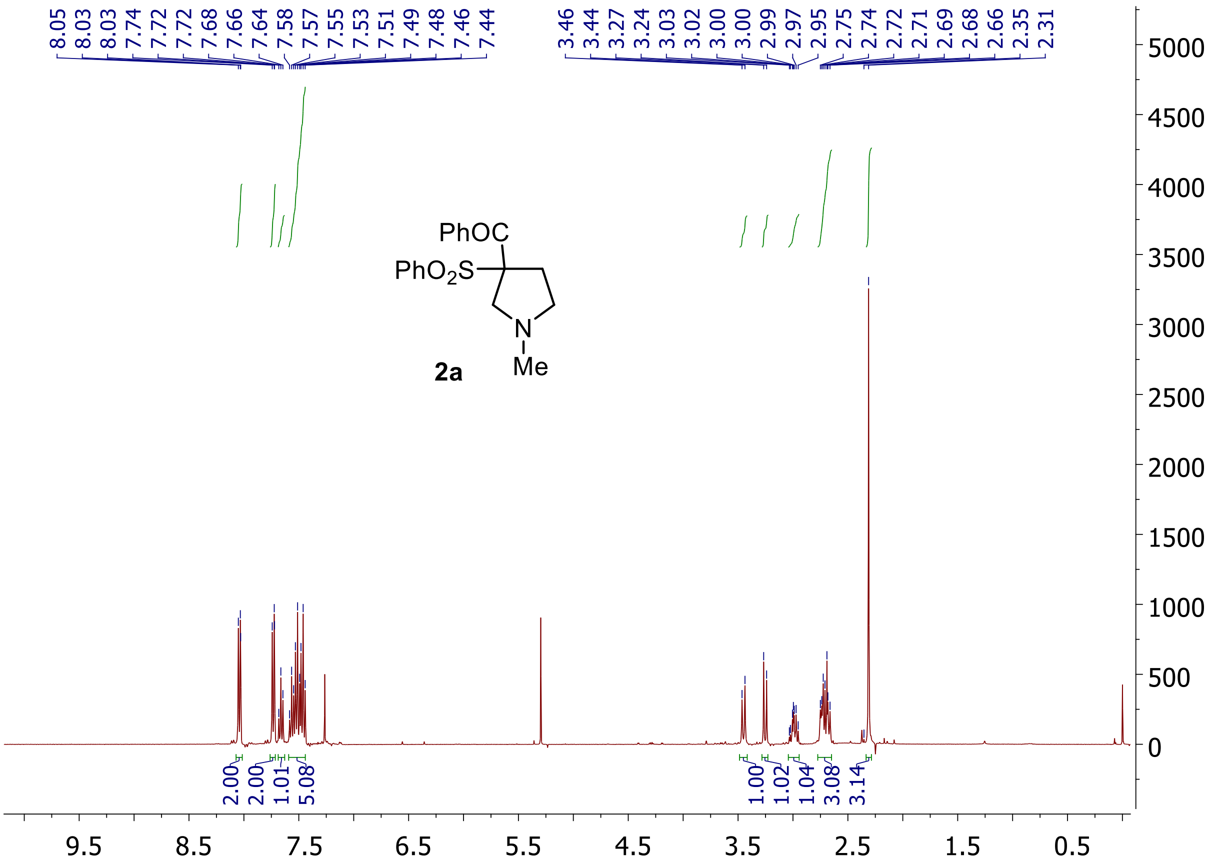
8.05 8.03 8.03 7.74 7.72 7.72 7.68 7.66 7.64 7.58 7.57 7.55 7.53 7.51 7.49 7.48 7.46 7.44
3.46 3.44 3.27 3.24 3.03 3.02 3.00 3.00 2.99 2.97 2.95 2.75 2.74 2.72 2.71 2.69 2.68 2.66 2.35 2.31
PhOC
PhO2S
N
Me
2a
2.00 2.00 1.01 5.08
1.00 1.02 1.04 3.08 3.14

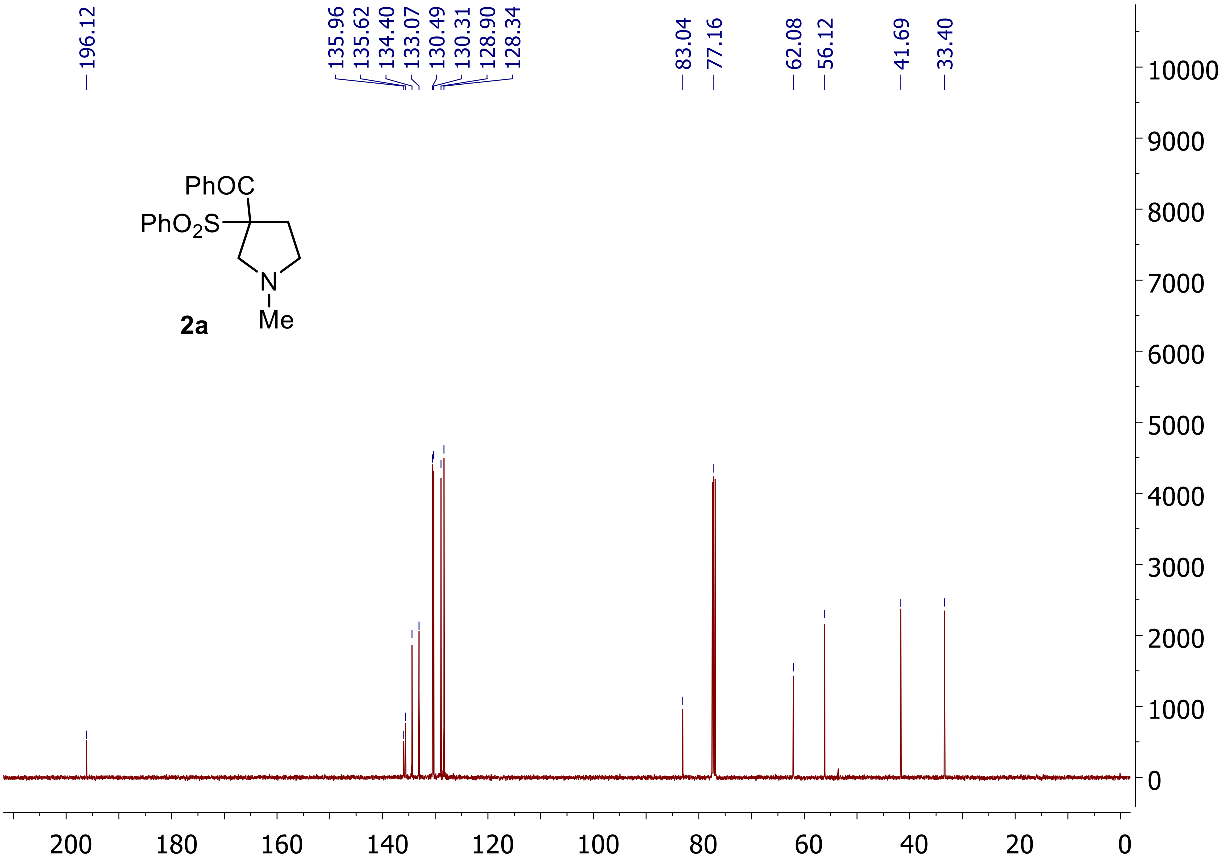
196.12
135.96 135.62 134.40 133.07 130.49 130.31 128.90 128.34
83.04 77.16 62.08 56.12 41.69 33.40
PhOC
PhO2S
N
Me
2a

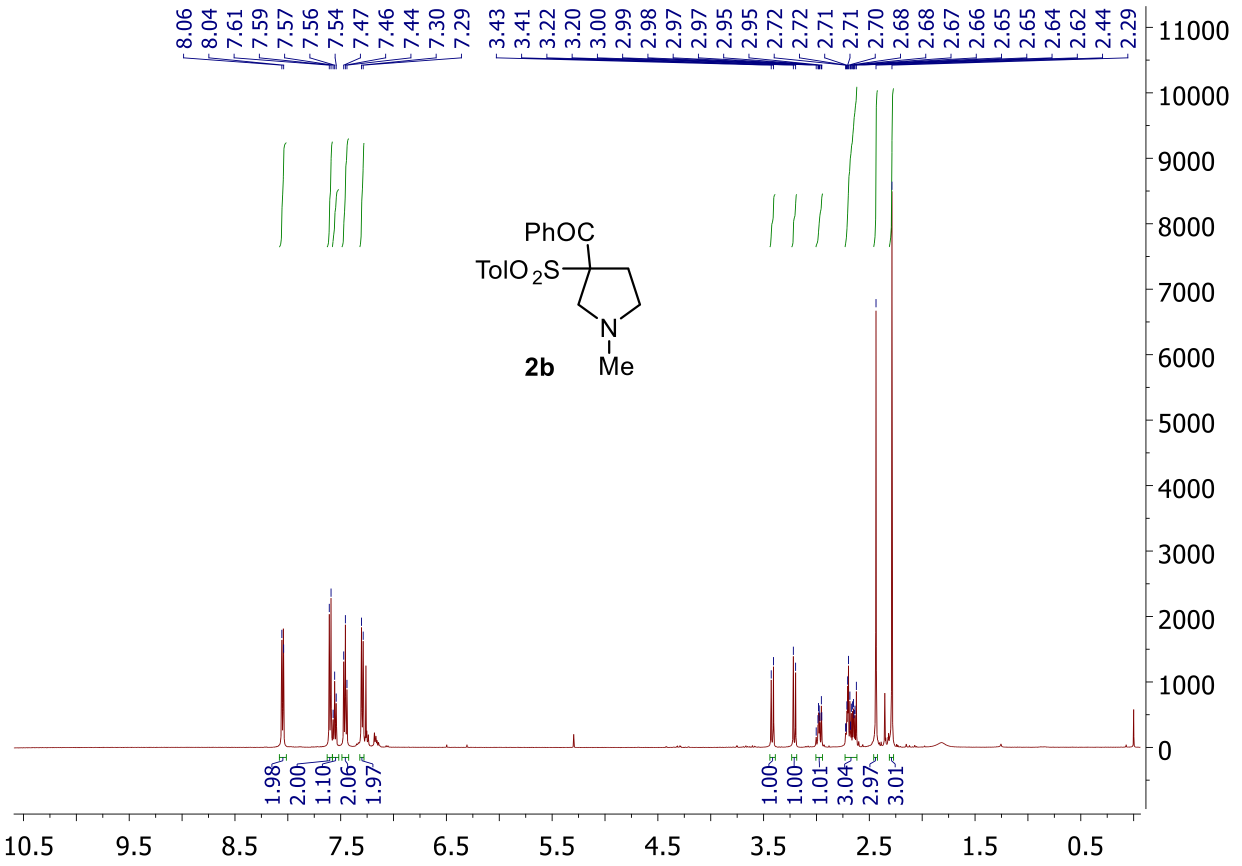
8.06 8.04 7.61 7.59 7.57 7.56 7.54 7.47 7.46 7.44 7.30 7.29
3.43 3.41 3.22 3.20 3.00 2.99 2.98 2.97 2.97 2.95 2.95 2.72 2.72 2.71 2.71 2.70 2.68 2.68 2.67 2.66 2.65 2.65 2.64 2.62 2.44 2.29
PhOC
TolO2S
N
Me
2b
1.98 2.00 1.10 2.06 1.97
1.00 1.00 1.01 3.04 2.97 3.01
10.5 9.5 8.5 7.5 6.5 5.5 4.5 3.5 2.5 1.5 0.5
11000 10000 9000 8000 7000 6000 5000 4000 3000 2000 1000 0

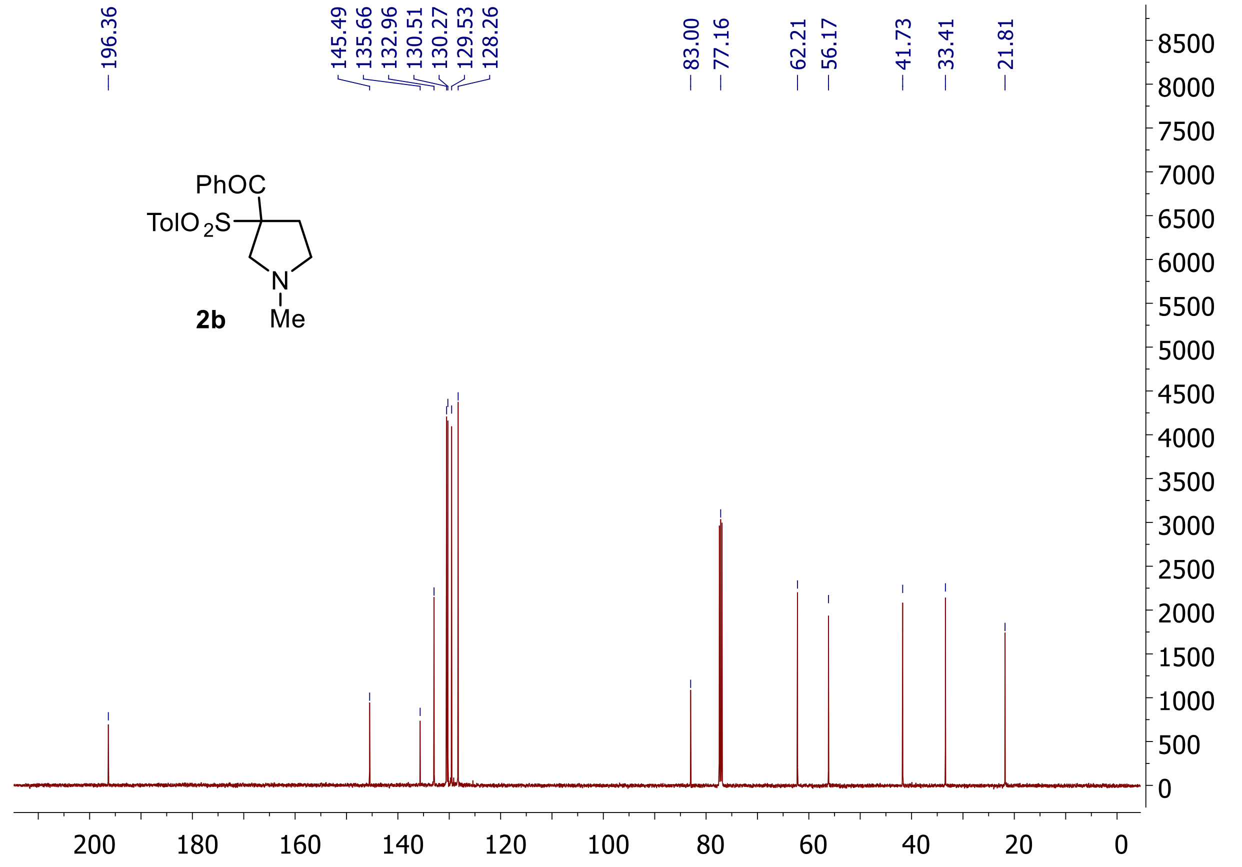
196.36
145.49 135.66 132.96 130.51 130.27 129.53 128.26
83.00 77.16
62.21 56.17
41.73 33.41 21.81
PhOC
TolO2S
N
Me
2b
200 180 160 140 120 100 80 60 40 20 0
8500 8000 7500 7000 6500 6000 5500 5000 4500 4000 3500 3000 2500 2000 1500 1000 500 0

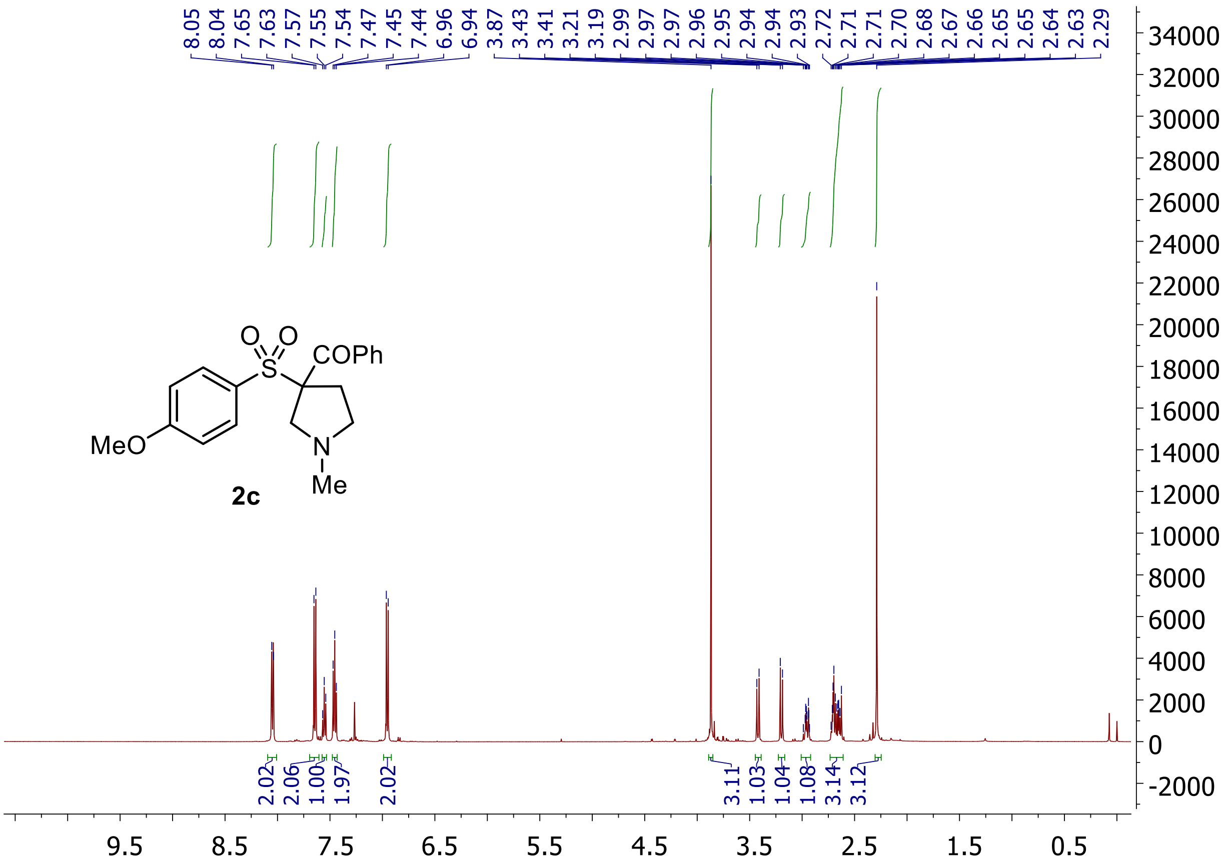
8.05 8.04 7.65 7.63 7.57 7.55 7.54 7.47 7.45 7.44 6.96 6.94 3.87 3.43 3.41 3.21 3.19 2.99 2.97 2.97 2.96 2.95 2.94 2.94 2.93 2.72 2.71 2.71 2.70 2.68 2.67 2.66 2.65 2.65 2.64 2.63 2.29
2.02 2.06 1.00 1.97 2.02 3.11 1.03 1.04 1.08 3.14 3.12
MeO
COPh
N
Me
2c

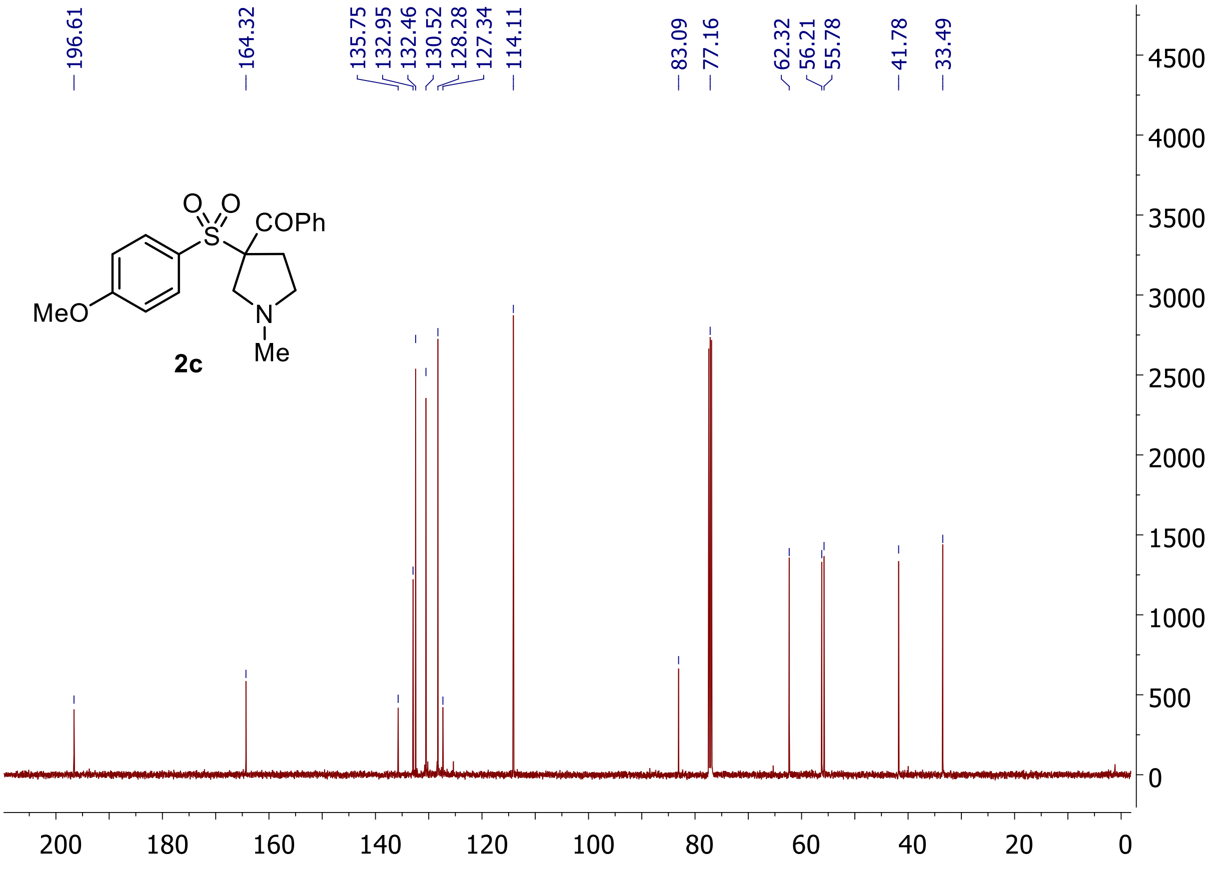
196.61 164.32 135.75 132.95 132.46 130.52 128.28 127.34 114.11 83.09 77.16 62.32 56.21 55.78 41.78 33.49
MeO
COPh
N
Me
2c

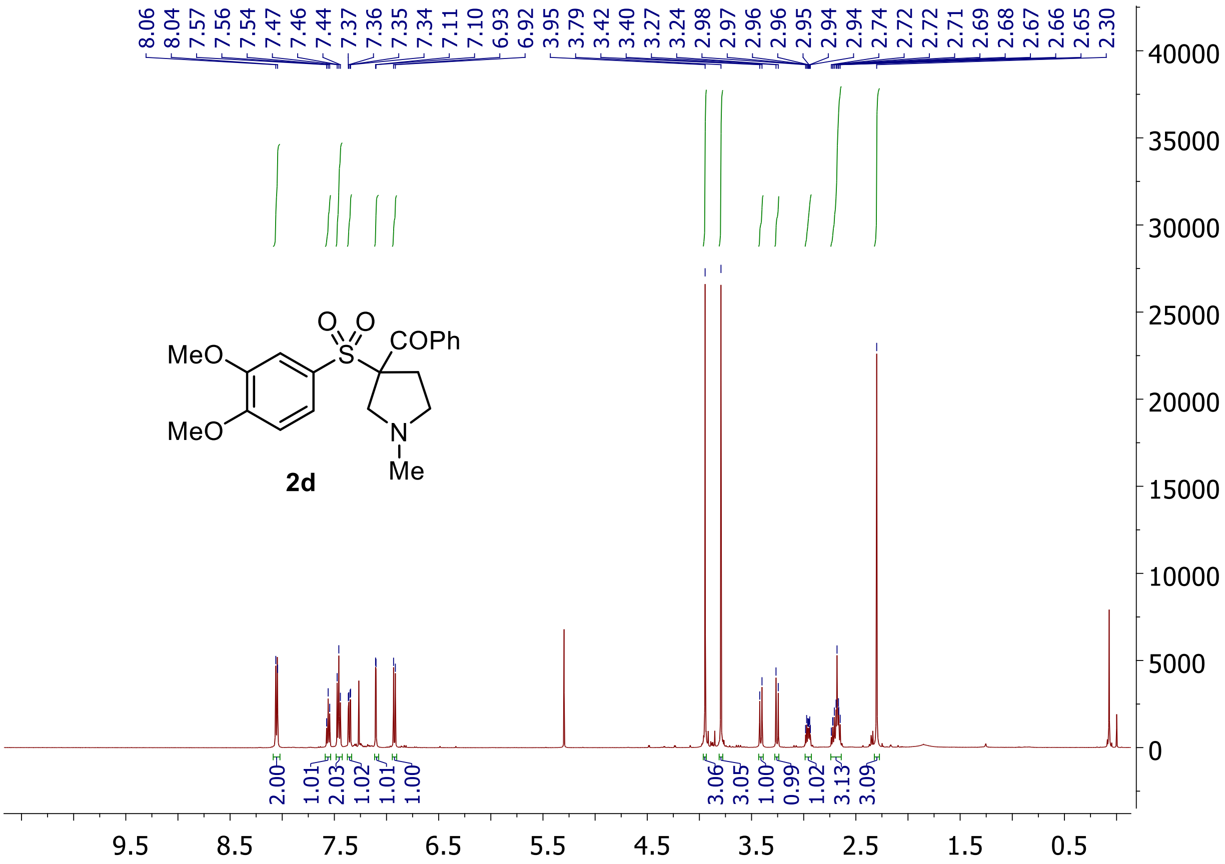
8.06 8.04 7.57 7.56 7.54 7.47 7.46 7.44 7.37 7.36 7.35 7.34 7.11 7.10 6.93 6.92 3.95 3.79 3.42 3.40 3.27 3.24 2.98 2.97 2.96 2.96 2.95 2.94 2.94 2.74 2.72 2.72 2.71 2.69 2.68 2.67 2.66 2.65 2.30
MeO
MeO
COPh
N
Me
2d
2.00 1.01 2.03 1.02 1.01 1.00 3.06 3.05 1.00 0.99 1.02 3.13 3.09
9.5 8.5 7.5 6.5 5.5 4.5 3.5 2.5 1.5 0.5
40000 35000 30000 25000 20000 15000 10000 5000 0

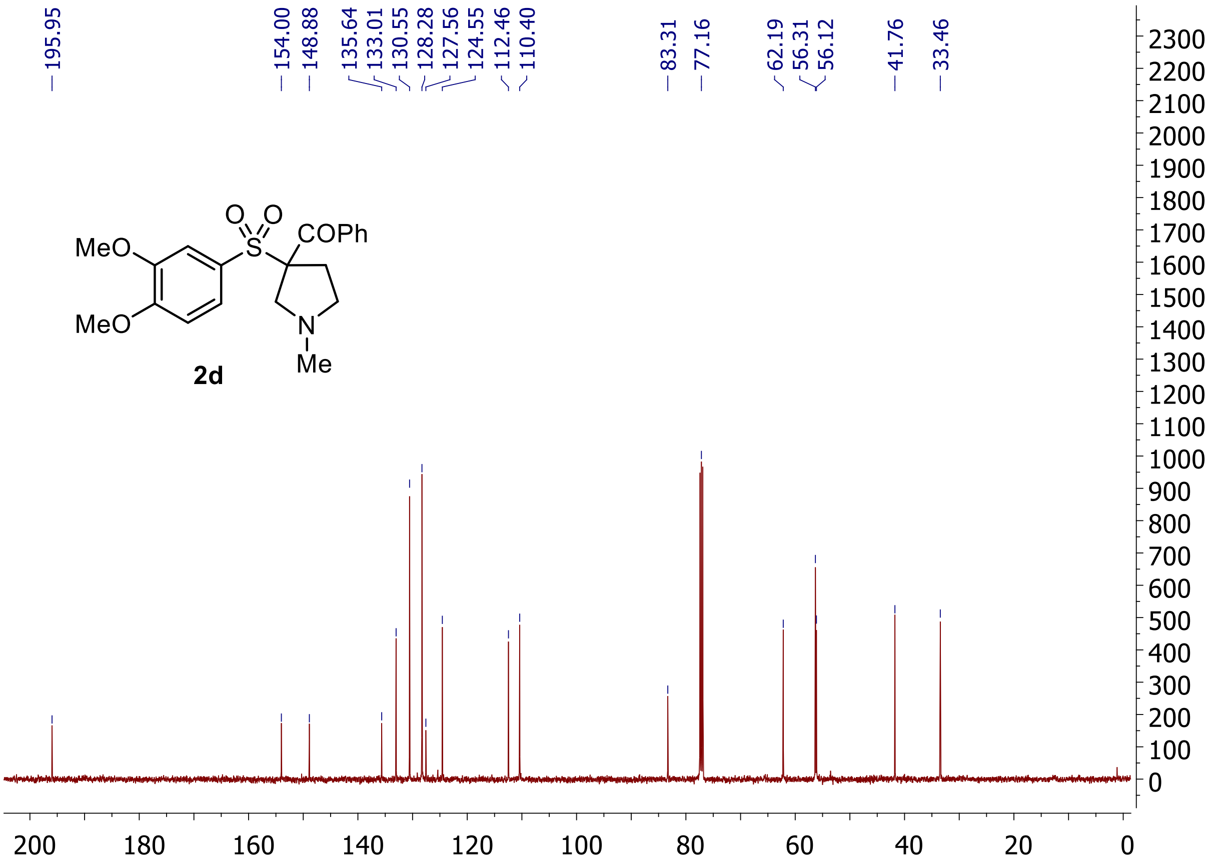
195.95 154.00 148.88 135.64 133.01 130.55 128.28 127.56 124.55 112.46 110.40 83.31 77.16 62.19 56.31 56.12 41.76 33.46
MeO
MeO
COPh
N
Me
2d
200 180 160 140 120 100 80 60 40 20 0
2300 2200 2100 2000 1900 1800 1700 1600 1500 1400 1300 1200 1100 1000 900 800 700 600 500 400 300 200 100 0

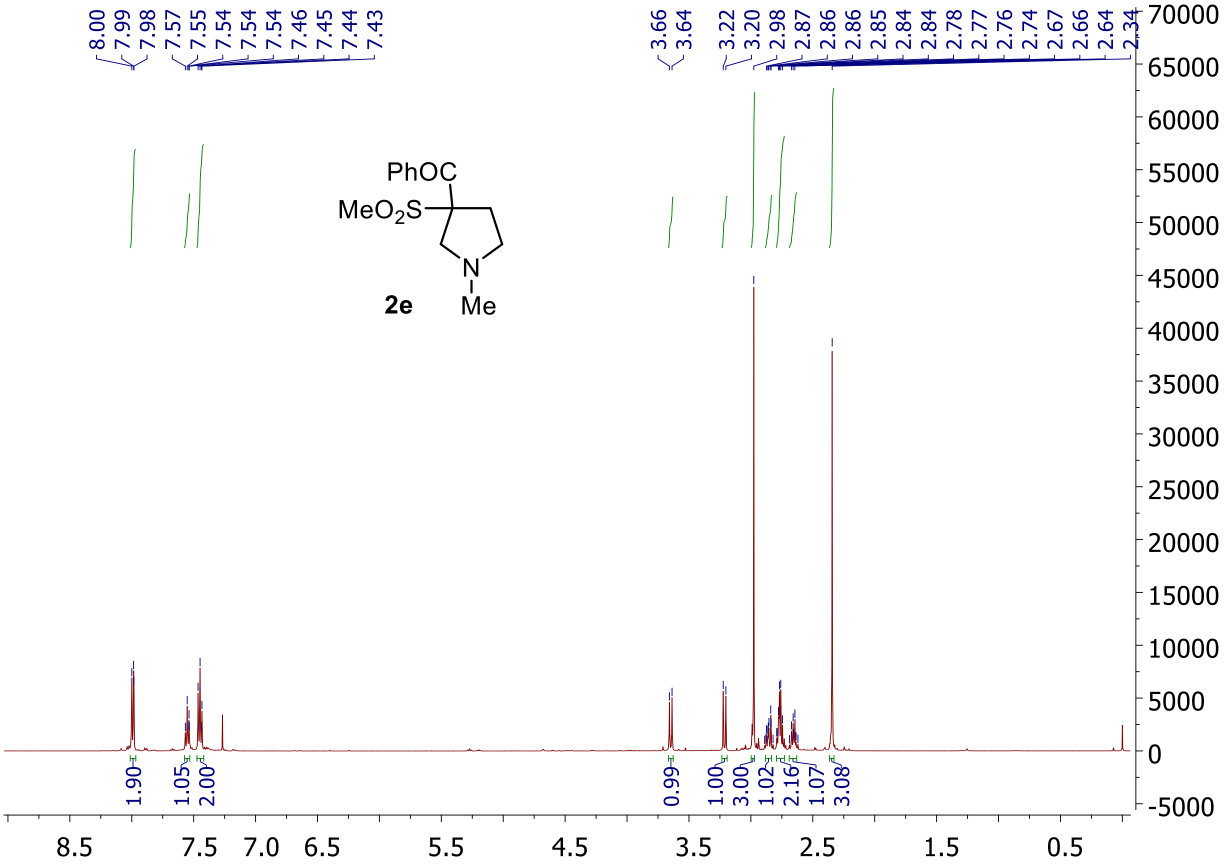
8.00
7.99
7.98
7.57
7.55
7.54
7.54
7.54
7.46
7.45
7.44
7.43
3.66
3.64
3.22
3.20
2.98
2.87
2.86
2.86
2.85
2.84
2.84
2.78
2.77
2.76
2.74
2.67
2.66
2.64
2.34
PhOC
MeO2S
N
Me
2e
1.90
1.05
2.00
0.99
1.00
3.00
1.02
2.16
1.07
3.08

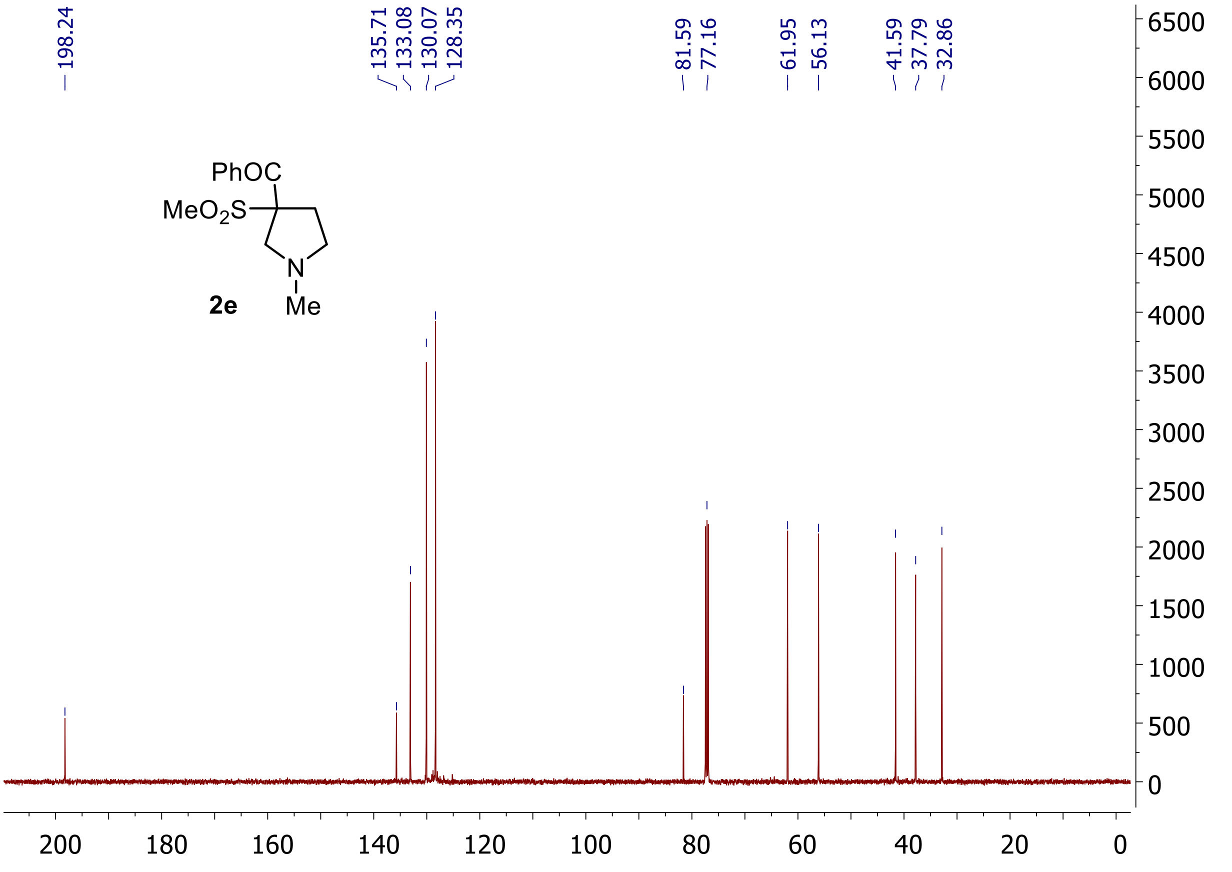
198.24
135.71
133.08
130.07
128.35
81.59
77.16
61.95
56.13
41.59
37.79
32.86
PhOC
MeO2S
N
Me
2e

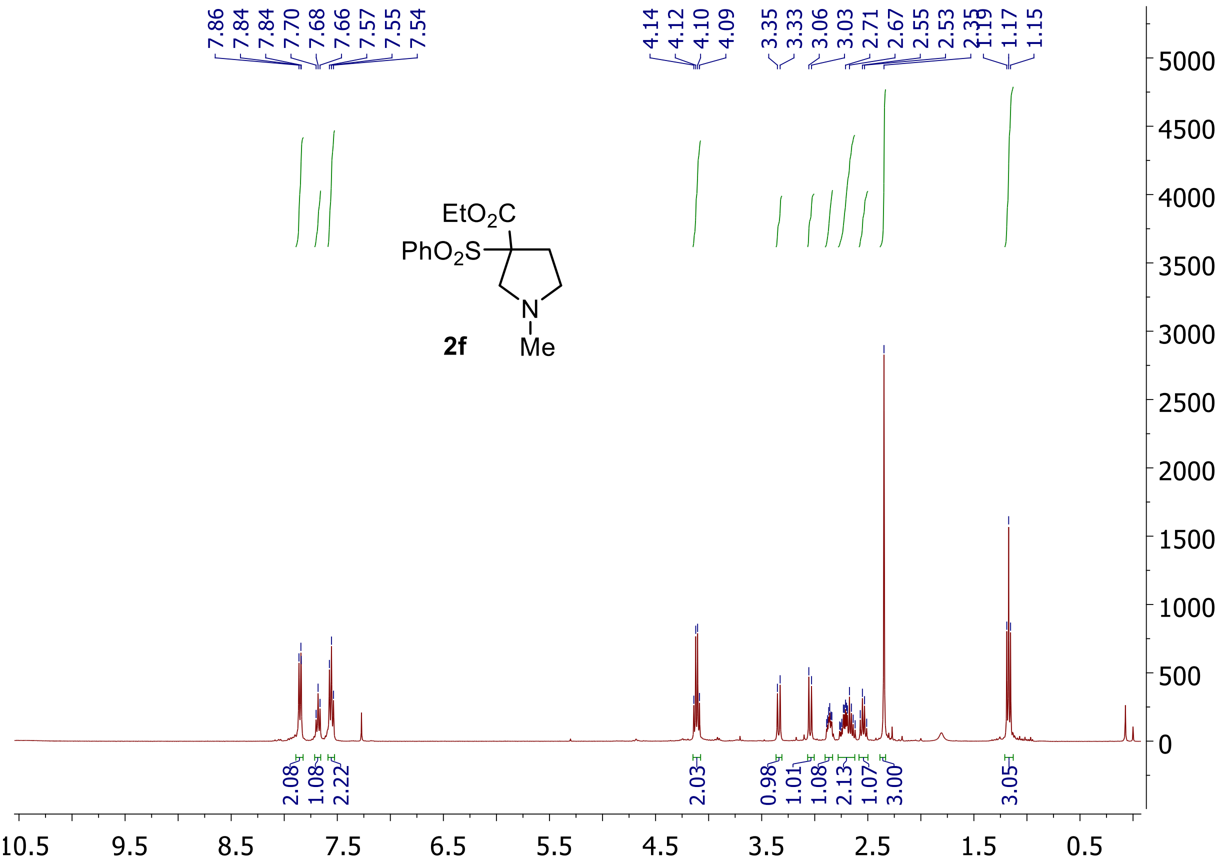
EtO2C
PhO2S
N
Me
2f

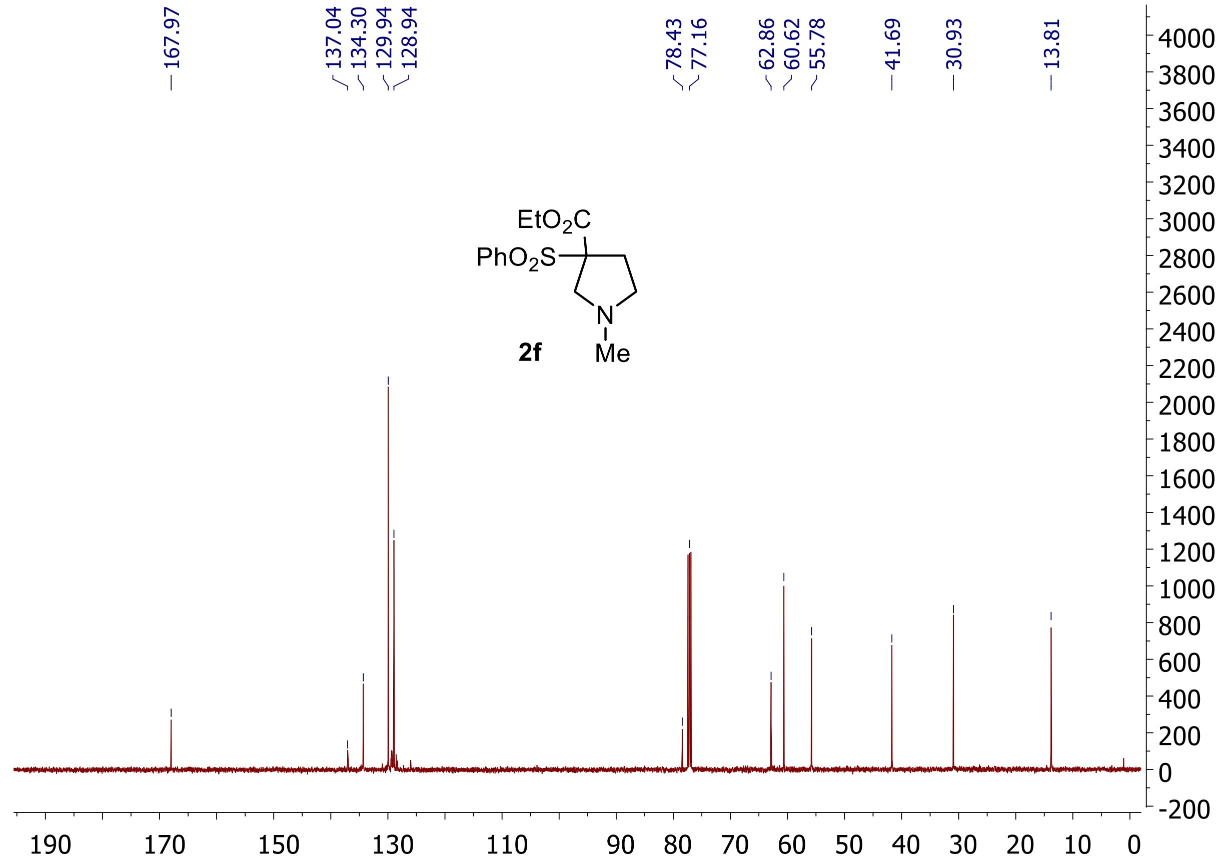
EtO2C
PhO2S
N
Me
2f

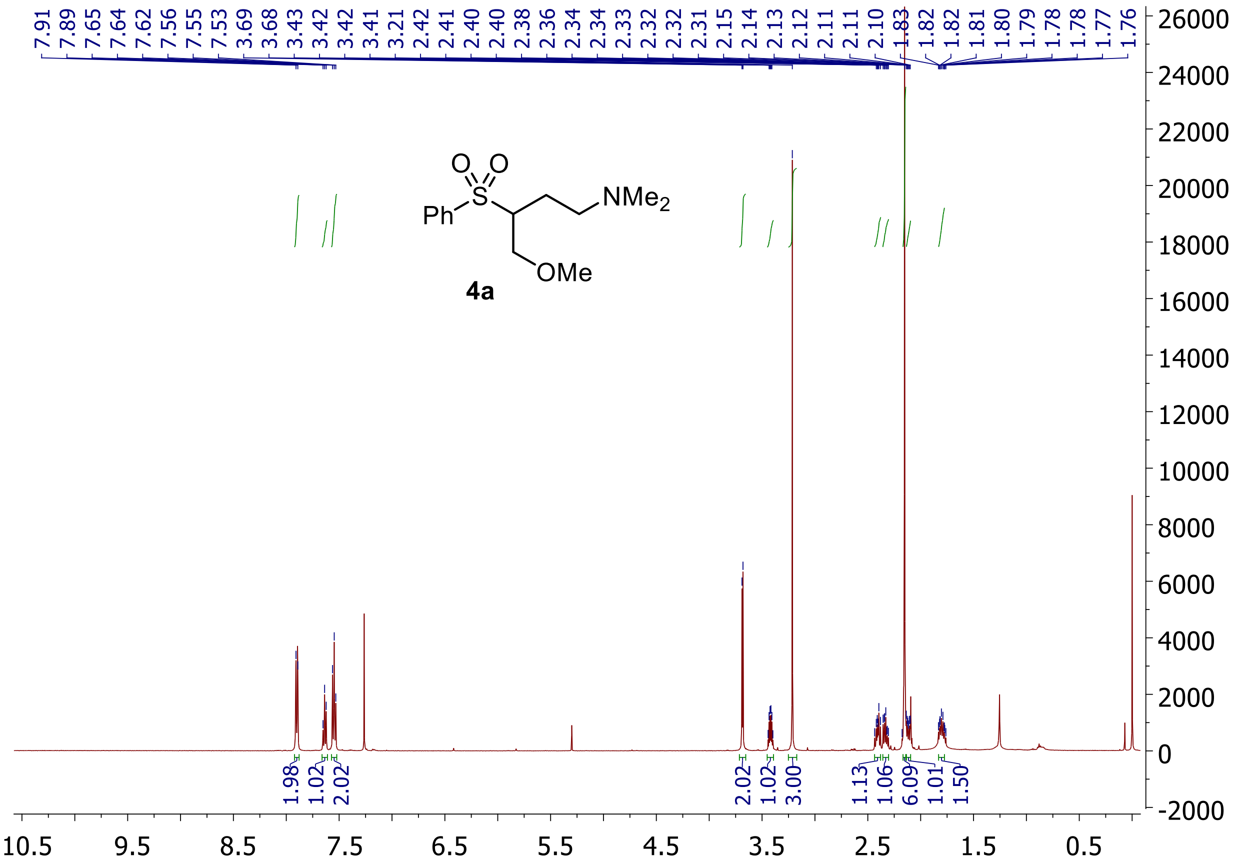
7.91 7.89 7.65 7.64 7.62 7.56 7.55 7.53 3.69 3.68 3.43 3.42 3.42 3.41 3.21 2.42 2.41 2.40 2.40 2.38 2.36 2.34 2.34 2.33 2.32 2.32 2.31 2.15 2.14 2.13 2.12 2.11 2.11 2.10 1.83 1.82 1.82 1.81 1.80 1.79 1.78 1.78 1.77 1.76
Ph
S
O O
NMe2
OMe
4a
1.98 1.02 2.02
2.02 1.02 3.00
1.13 1.06 6.09 1.01 1.50
10.5 9.5 8.5 7.5 6.5 5.5 4.5 3.5 2.5 1.5 0.5
26000 24000 22000 20000 18000 16000 14000 12000 10000 8000 6000 4000 2000 0 -2000

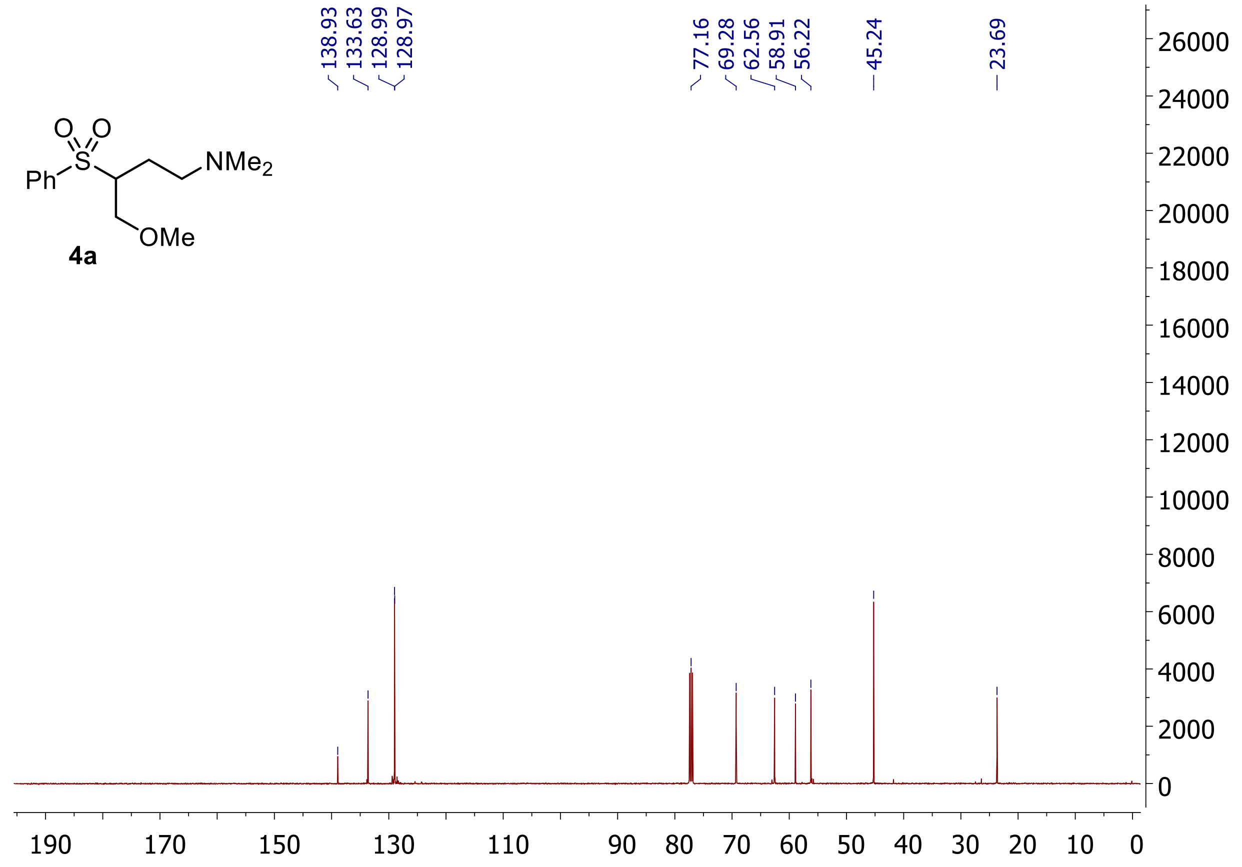
138.93 133.63 128.99 128.97
77.16 69.28 62.56 58.91 56.22
45.24
23.69
Ph
S
O O
NMe2
OMe
4a
190 170 150 130 110 90 80 70 60 50 40 30 20 10 0
26000 24000 22000 20000 18000 16000 14000 12000 10000 8000 6000 4000 2000 0

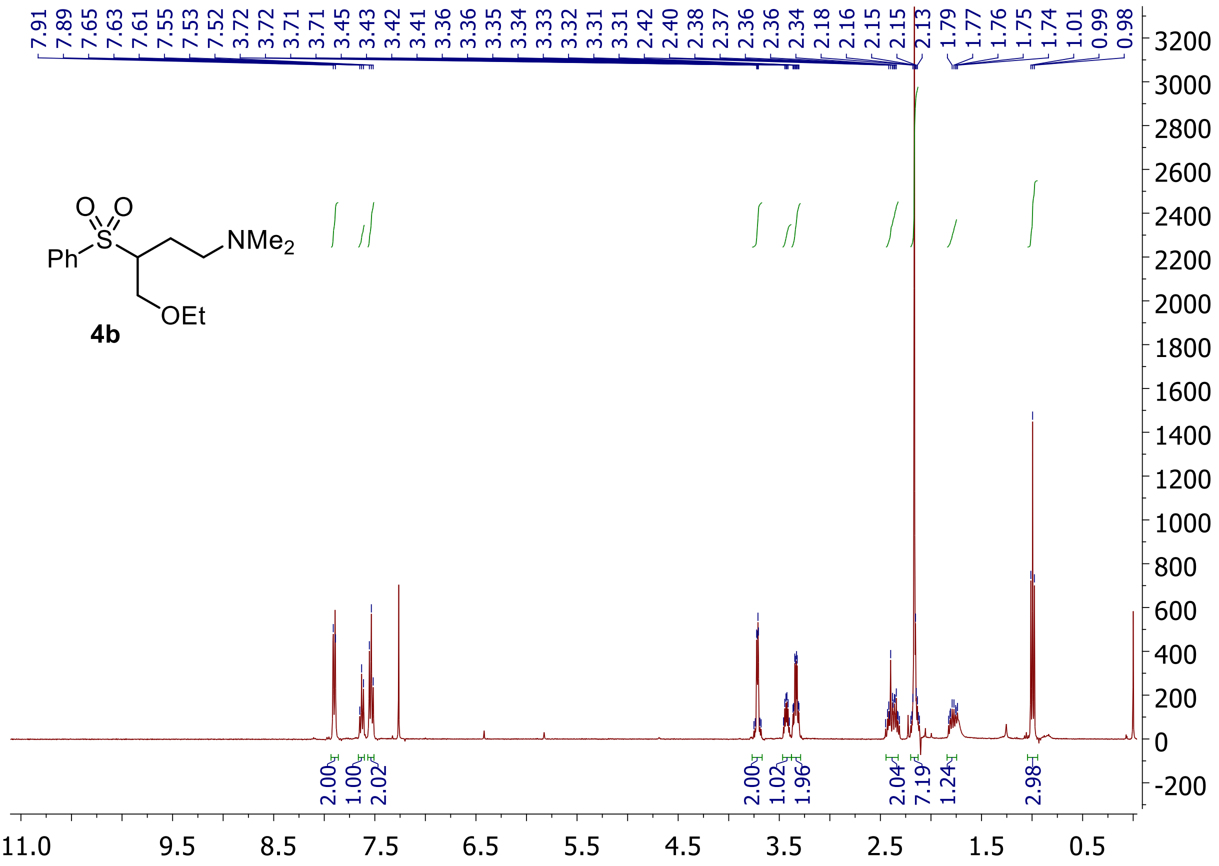

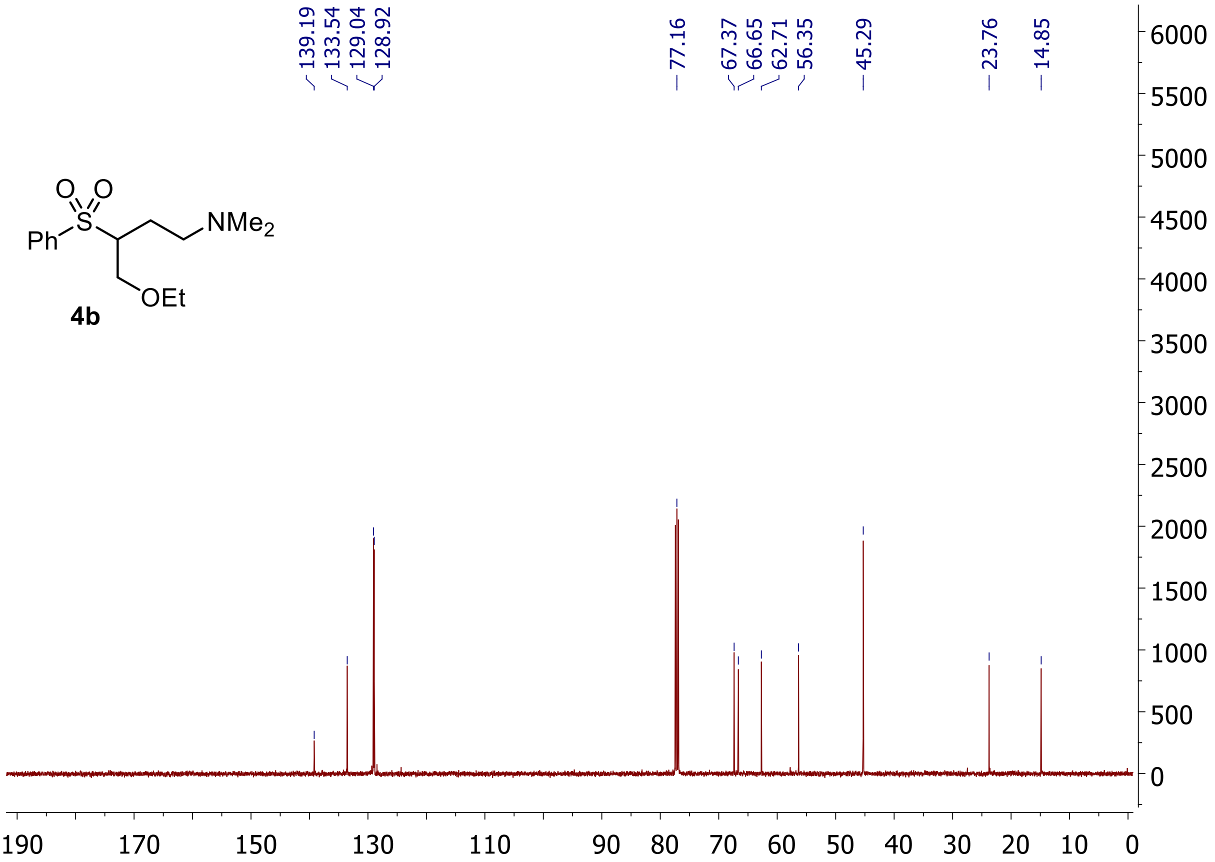

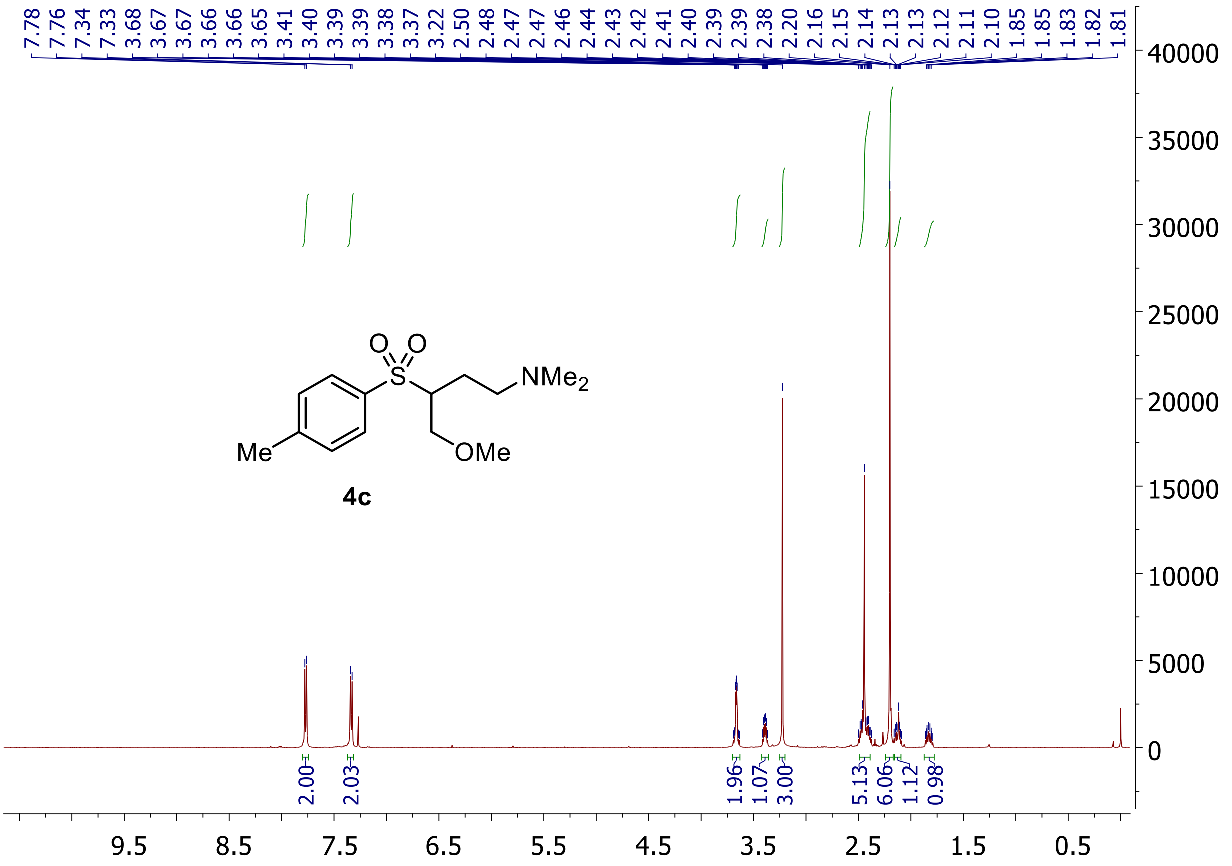
7.78 7.76 7.34 7.33 3.68 3.67 3.67 3.66 3.66 3.65 3.41 3.40 3.39 3.39 3.38 3.37 3.22 2.50 2.48 2.47 2.47 2.46 2.44 2.43 2.42 2.41 2.40 2.39 2.39 2.38 2.20 2.16 2.15 2.14 2.13 2.13 2.12 2.11 2.10 1.85 1.85 1.83 1.82 1.81
NMe2
Me
OMe
4c
2.00 2.03 1.96 1.07 3.00 5.13 6.06 1.12 0.98
9.5 8.5 7.5 6.5 5.5 4.5 3.5 2.5 1.5 0.5
40000 35000 30000 25000 20000 15000 10000 5000 0

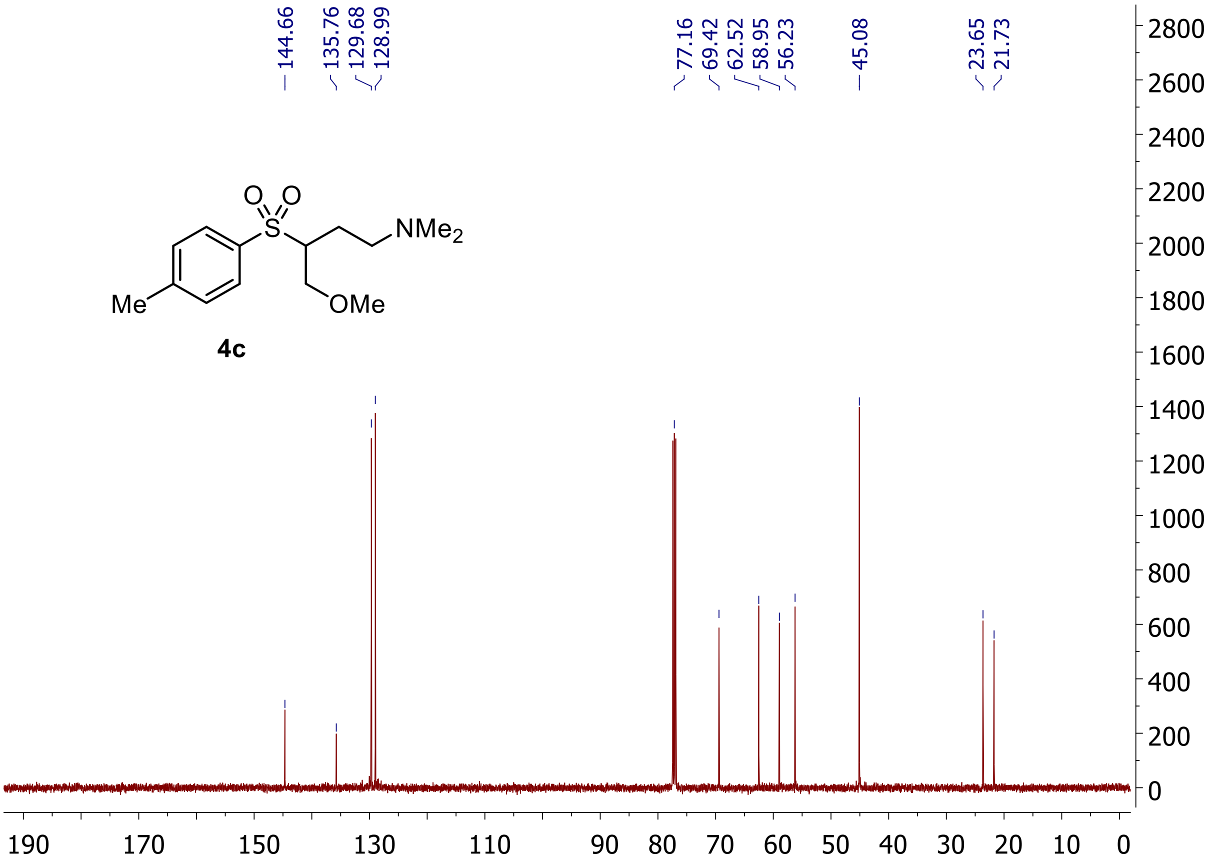
144.66 135.76 129.68 128.99 77.16 69.42 62.52 58.95 56.23 45.08 23.65 21.73
NMe2
Me
OMe
4c
190 170 150 130 110 90 80 70 60 50 40 30 20 10 0
2800 2600 2400 2200 2000 1800 1600 1400 1200 1000 800 600 400 200 0

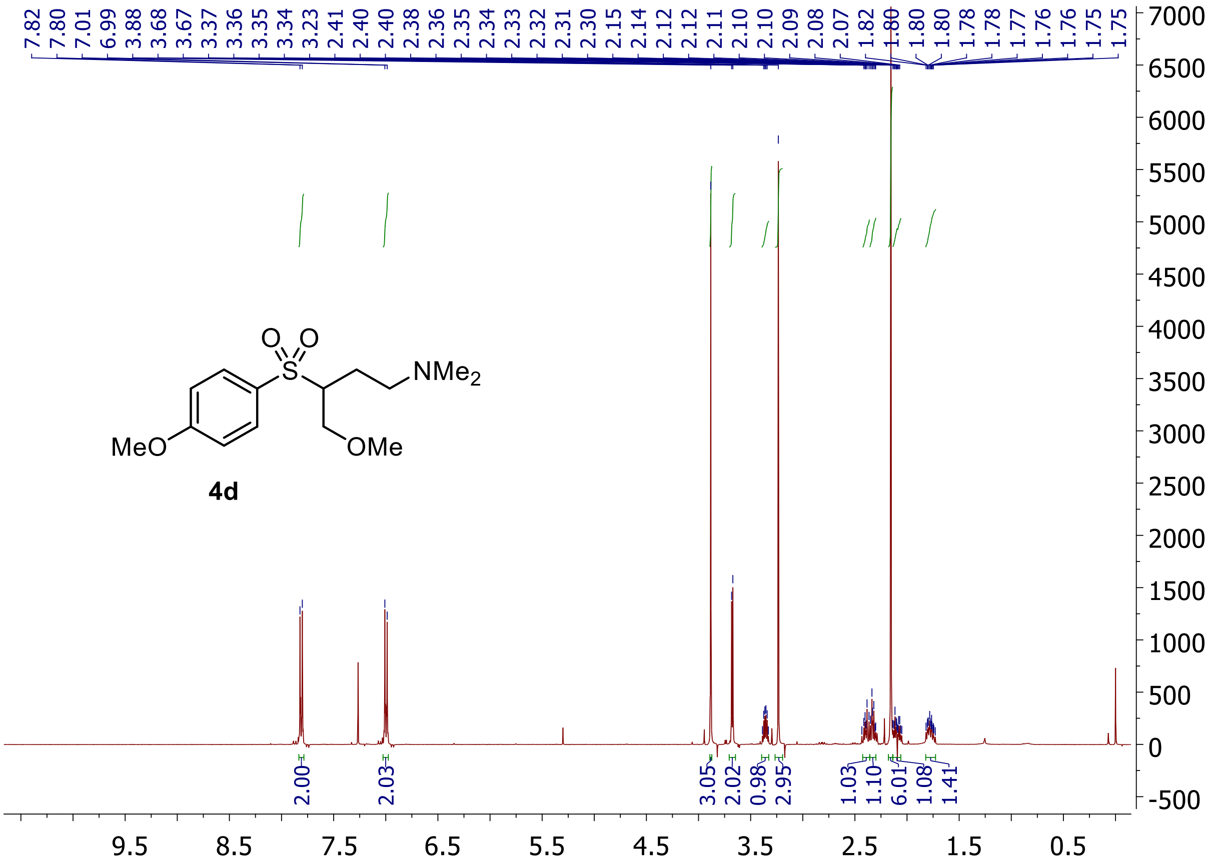
7.82
7.80
7.01
6.99
3.88
3.68
3.67
3.37
3.36
3.35
3.34
3.23
2.41
2.40
2.40
2.38
2.36
2.35
2.34
2.33
2.32
2.31
2.30
2.15
2.14
2.12
2.12
2.11
2.10
2.10
2.09
2.08
2.07
1.82
1.80
1.80
1.80
1.78
1.78
1.77
1.76
1.76
1.75
1.75
NMe2
MeO
OMe
4d
2.00
2.03
3.05
2.02
0.98
2.95
1.03
1.10
6.01
1.08
1.41
9.5
8.5
7.5
6.5
5.5
4.5
3.5
2.5
1.5
0.5
7000
6500
6000
5500
5000
4500
4000
3500
3000
2500
2000
1500
1000
500
0
-500

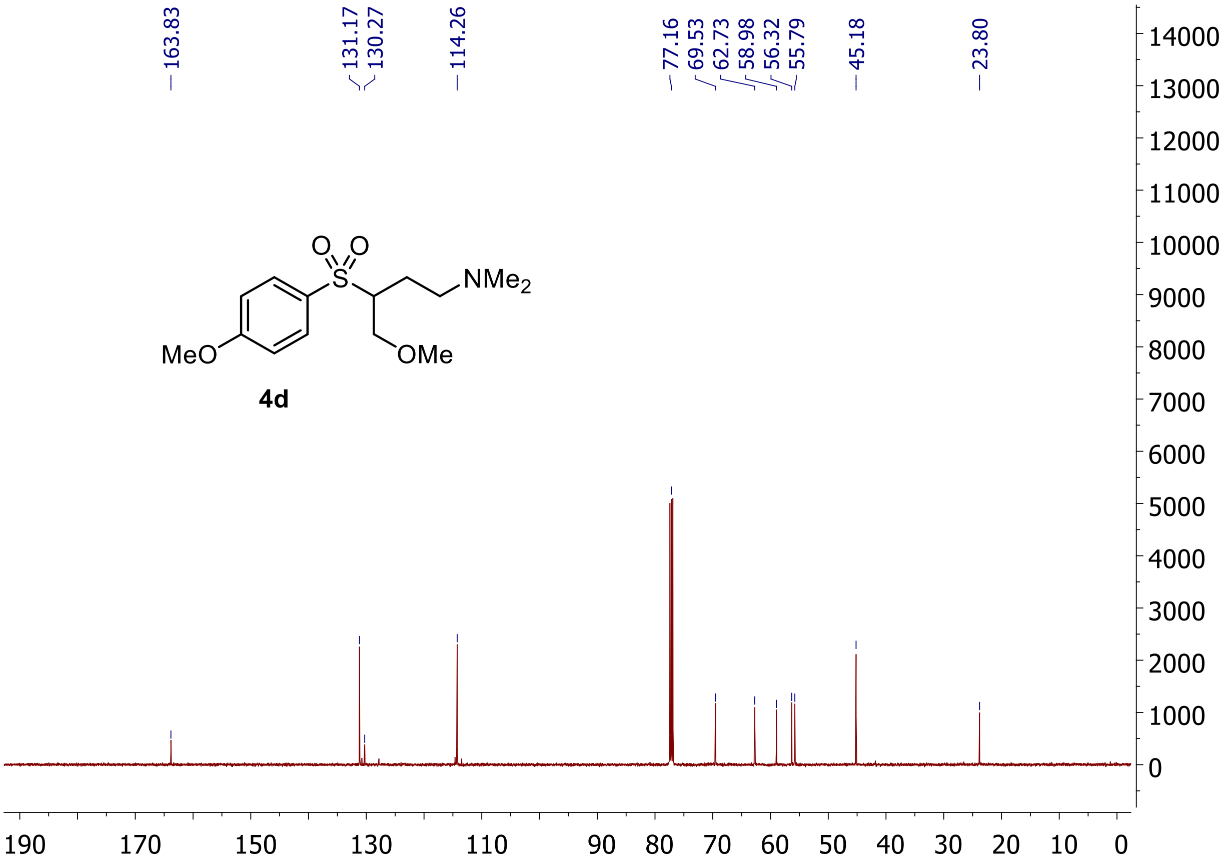
163.83
131.17
130.27
114.26
77.16
69.53
62.73
58.98
56.32
55.79
45.18
23.80
NMe2
MeO
OMe
4d
190
170
150
130
110
90
80
70
60
50
40
30
20
10
0
14000
13000
12000
11000
10000
9000
8000
7000
6000
5000
4000
3000
2000
1000
0

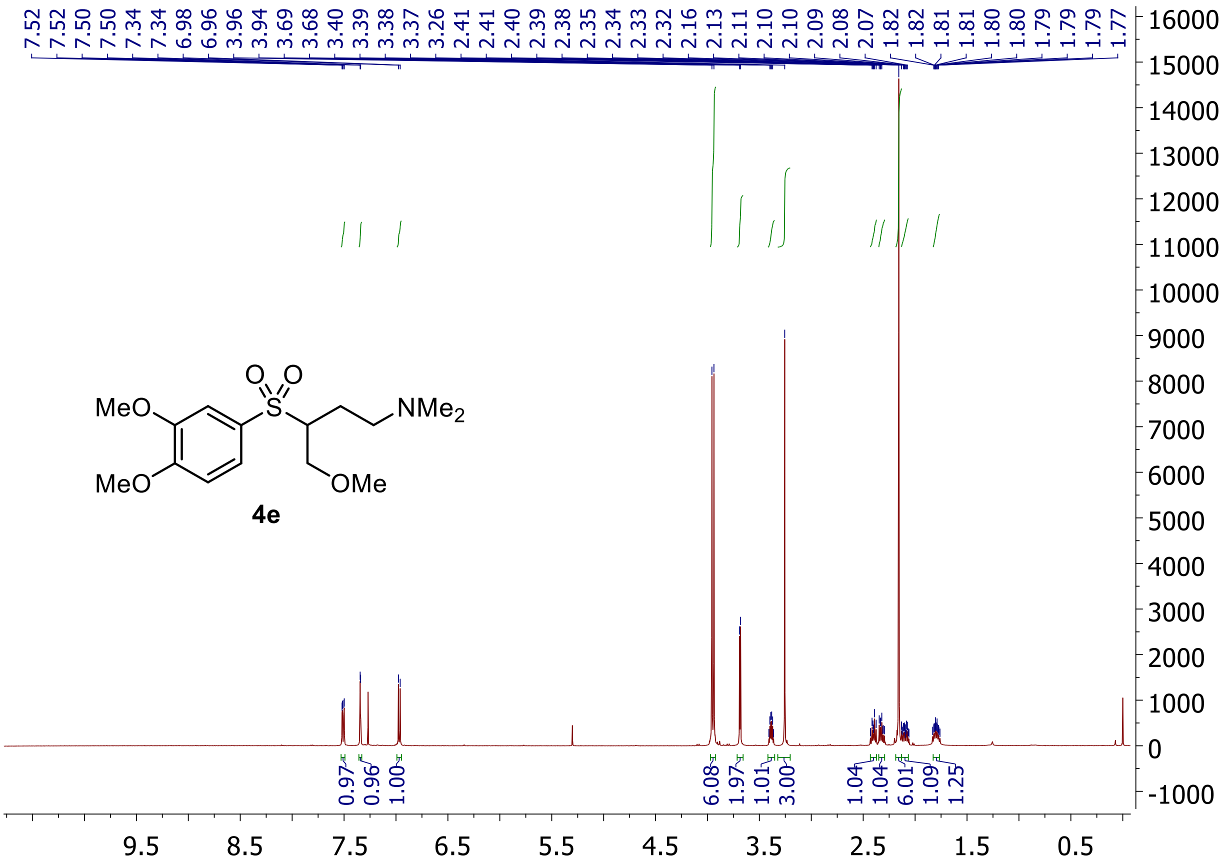
7.52 7.52 7.50 7.50 7.34 7.34 6.98 6.96 3.96 3.94 3.69 3.68 3.40 3.39 3.38 3.37 3.26 2.41 2.41 2.40 2.39 2.38 2.35 2.34 2.33 2.32 2.16 2.13 2.11 2.10 2.10 2.09 2.08 2.07 1.82 1.82 1.81 1.81 1.80 1.80 1.79 1.79 1.79 1.77
MeO
MeO
NMe2
OMe
4e
0.97 0.96 1.00 6.08 1.97 1.01 3.00 1.04 1.04 6.01 1.09 1.25
9.5 8.5 7.5 6.5 5.5 4.5 3.5 2.5 1.5 0.5

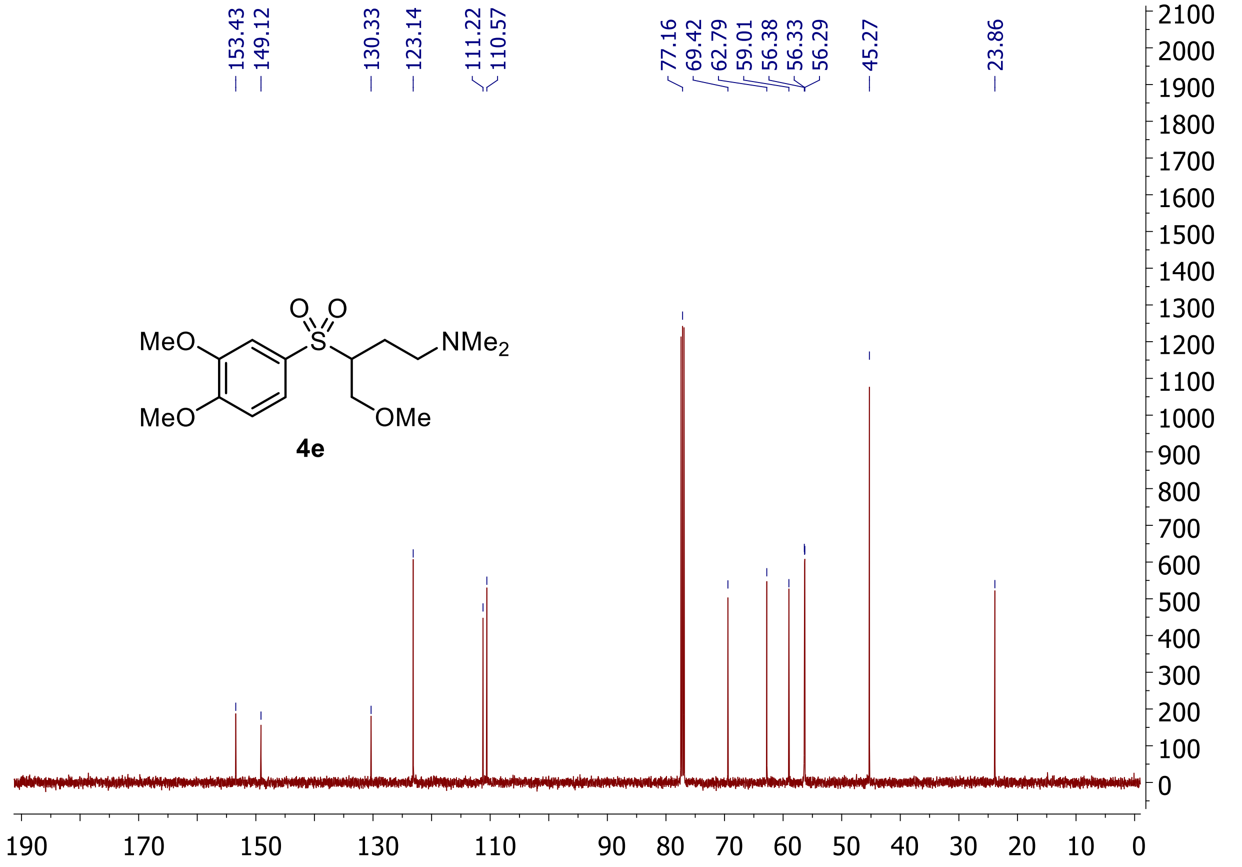
153.43 149.12 130.33 123.14 111.22 110.57 77.16 69.42 62.79 59.01 56.38 56.33 56.29 45.27 23.86
MeO
MeO
NMe2
OMe
4e
190 170 150 130 110 90 80 70 60 50 40 30 20 10 0

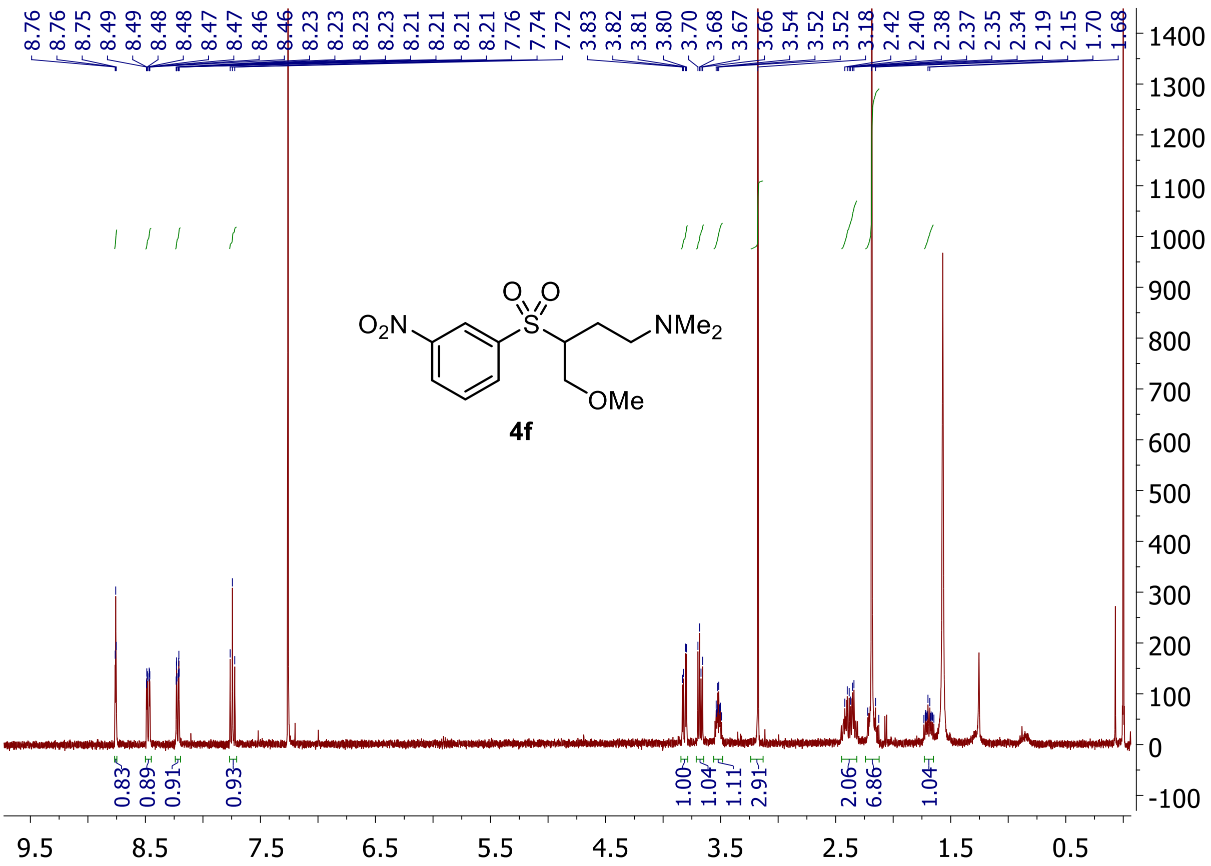
8.76 8.76 8.75 8.49 8.49 8.48 8.48 8.47 8.47 8.46 8.46 8.23 8.23 8.23 8.23 8.21 8.21 8.21 8.21 7.76 7.74 7.72 3.83 3.82 3.81 3.80 3.70 3.68 3.67 3.66 3.54 3.52 3.52 3.18 2.42 2.40 2.38 2.37 2.35 2.34 2.19 2.15 1.70 1.68
O$_2$N
NMe$_2$
OMe
4f
0.83 0.89 0.91 0.93 1.00 1.04 1.11 2.91 2.06 6.86 1.04
9.5 8.5 7.5 6.5 5.5 4.5 3.5 2.5 1.5 0.5

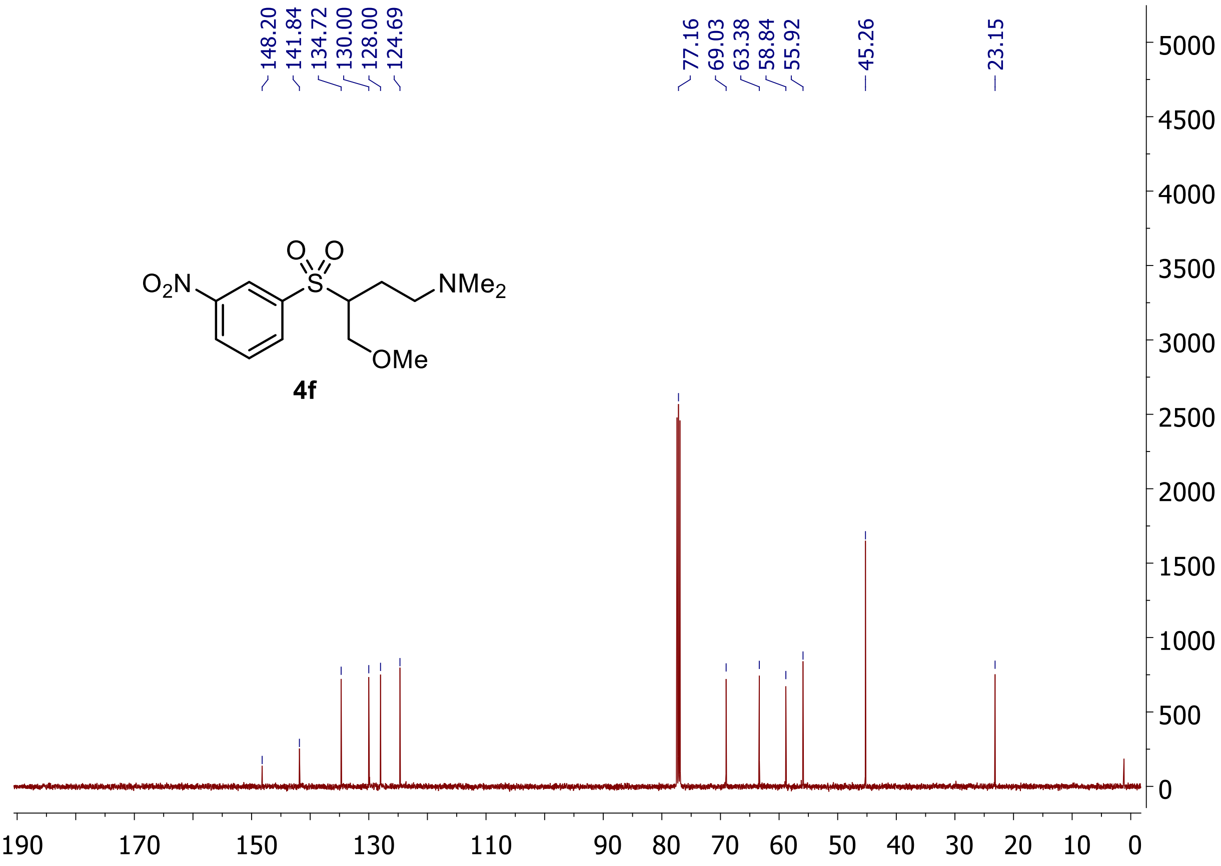
148.20 141.84 134.72 130.00 128.00 124.69 77.16 69.03 63.38 58.84 55.92 45.26 23.15
O$_2$N
NMe$_2$
OMe
4f
190 170 150 130 110 90 80 70 60 50 40 30 20 10 0

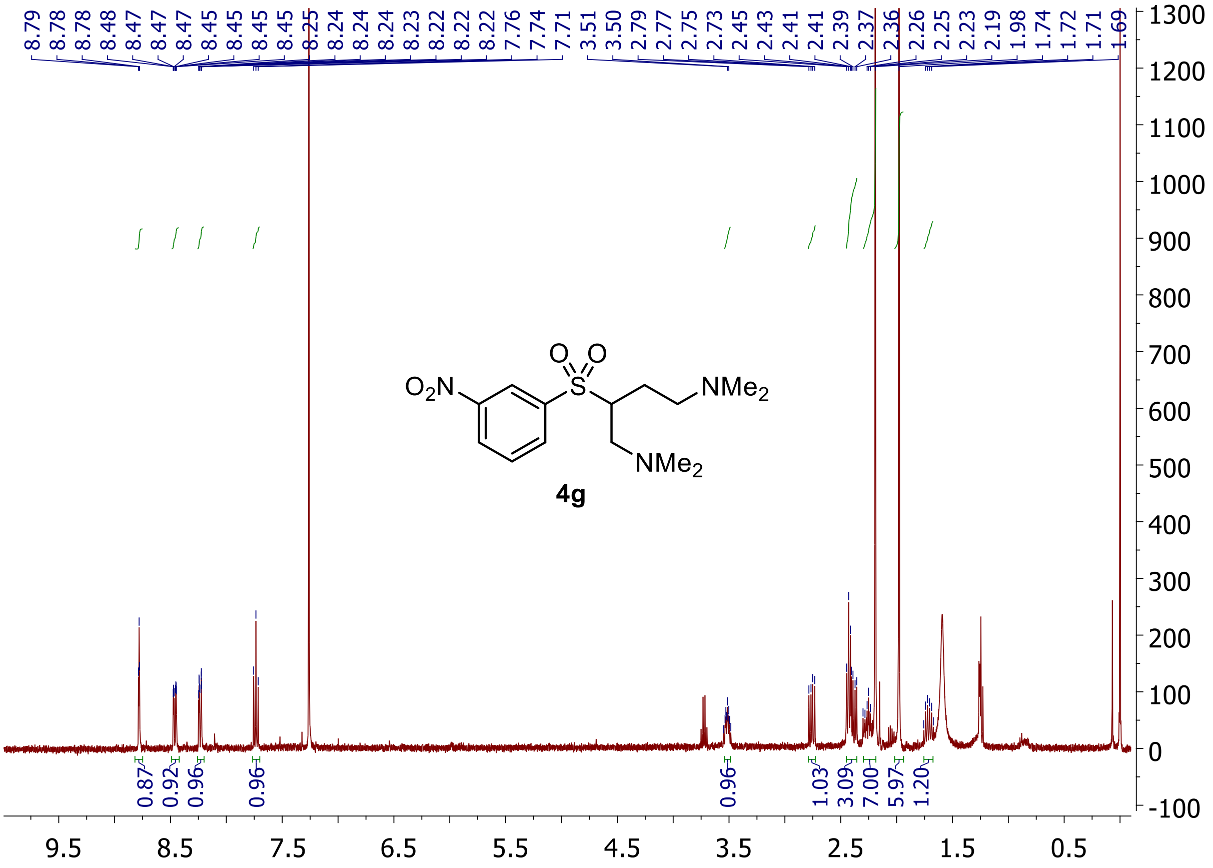

8.79
8.78
8.78
8.48
8.47
8.47
8.47
8.45
8.45
8.45
8.45
8.25
8.24
8.24
8.24
8.23
8.22
8.22
8.22
7.76
7.74
7.71
3.51
3.50
2.79
2.77
2.75
2.73
2.45
2.43
2.41
2.41
2.39
2.37
2.36
2.26
2.25
2.23
2.19
1.98
1.74
1.72
1.71
1.69
O2N
O
O
S
NMe2
NMe2
4g
0.87
0.92
0.96
0.96
0.96
1.03
3.09
7.00
5.97
1.20

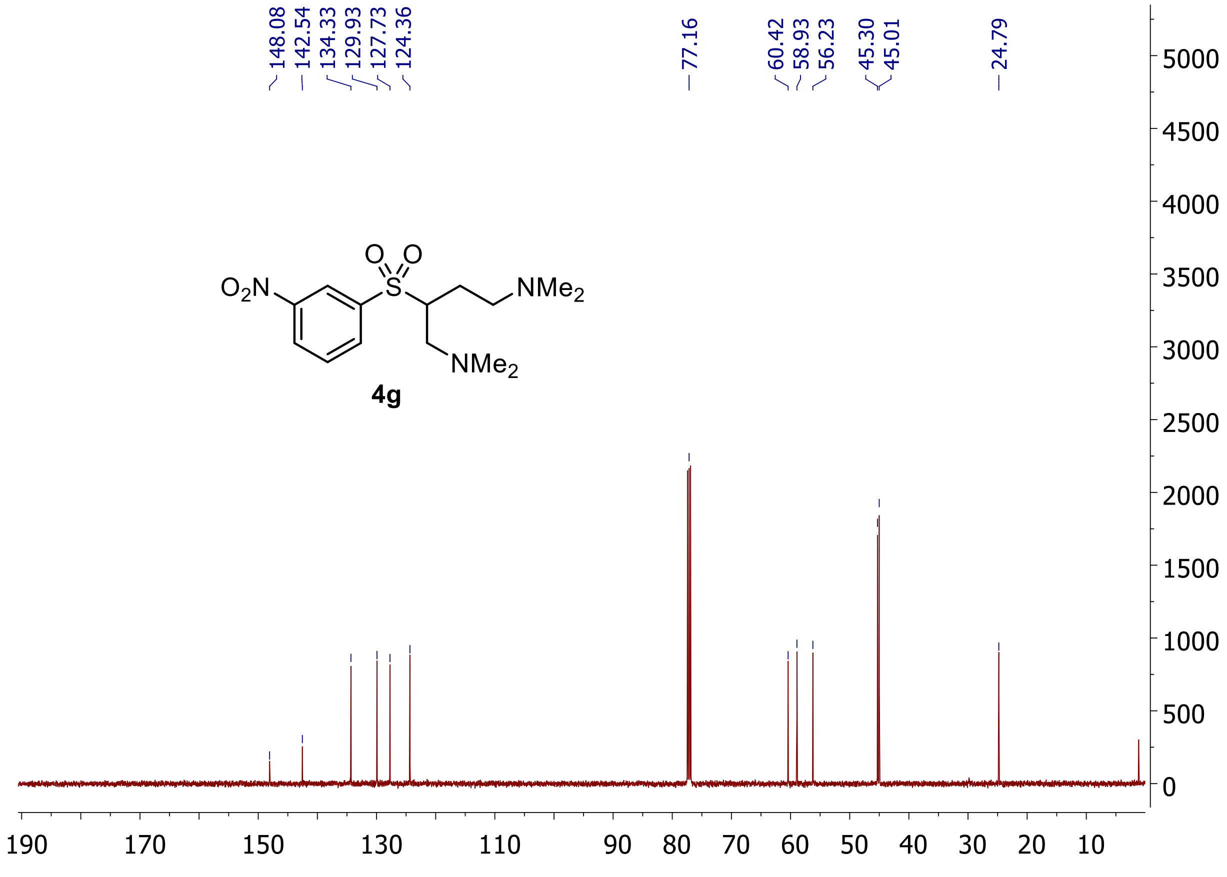

148.08
142.54
134.33
129.93
127.73
124.36
77.16
60.42
58.93
56.23
45.30
45.01
24.79
O2N
O
O
S
NMe2
NMe2
4g

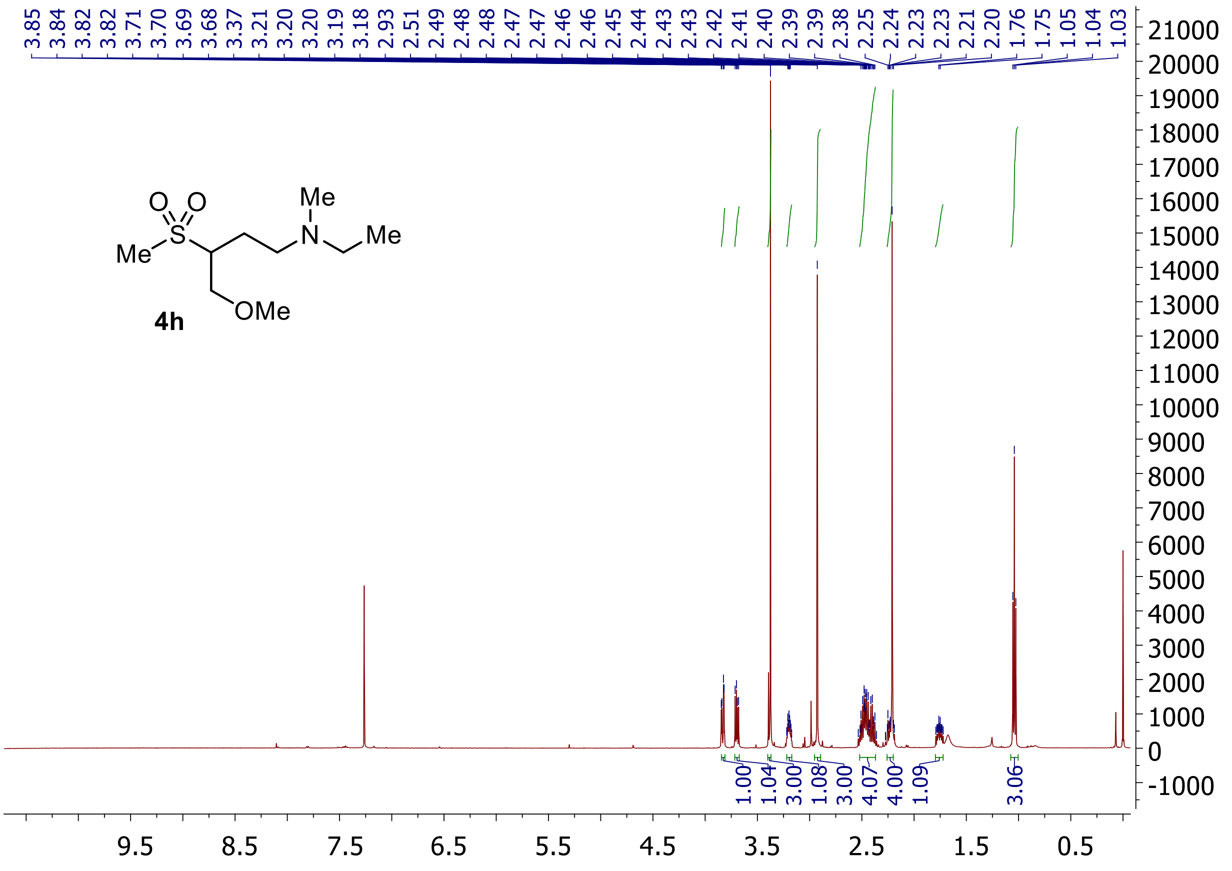

3.85 3.84 3.82 3.82 3.71 3.70 3.69 3.68 3.37 3.21 3.20 3.20 3.19 3.18 2.93 2.51 2.49 2.48 2.48 2.47 2.47 2.46 2.46 2.45 2.44 2.43 2.43 2.42 2.41 2.40 2.39 2.39 2.38 2.25 2.24 2.23 2.23 2.21 2.20 1.76 1.75 1.05 1.04 1.03
Me
O O
S
Me
N
Me
OMe
4h
1.00 1.04 3.00 1.08 3.00 4.07 4.00 1.09 3.06
9.5 8.5 7.5 6.5 5.5 4.5 3.5 2.5 1.5 0.5

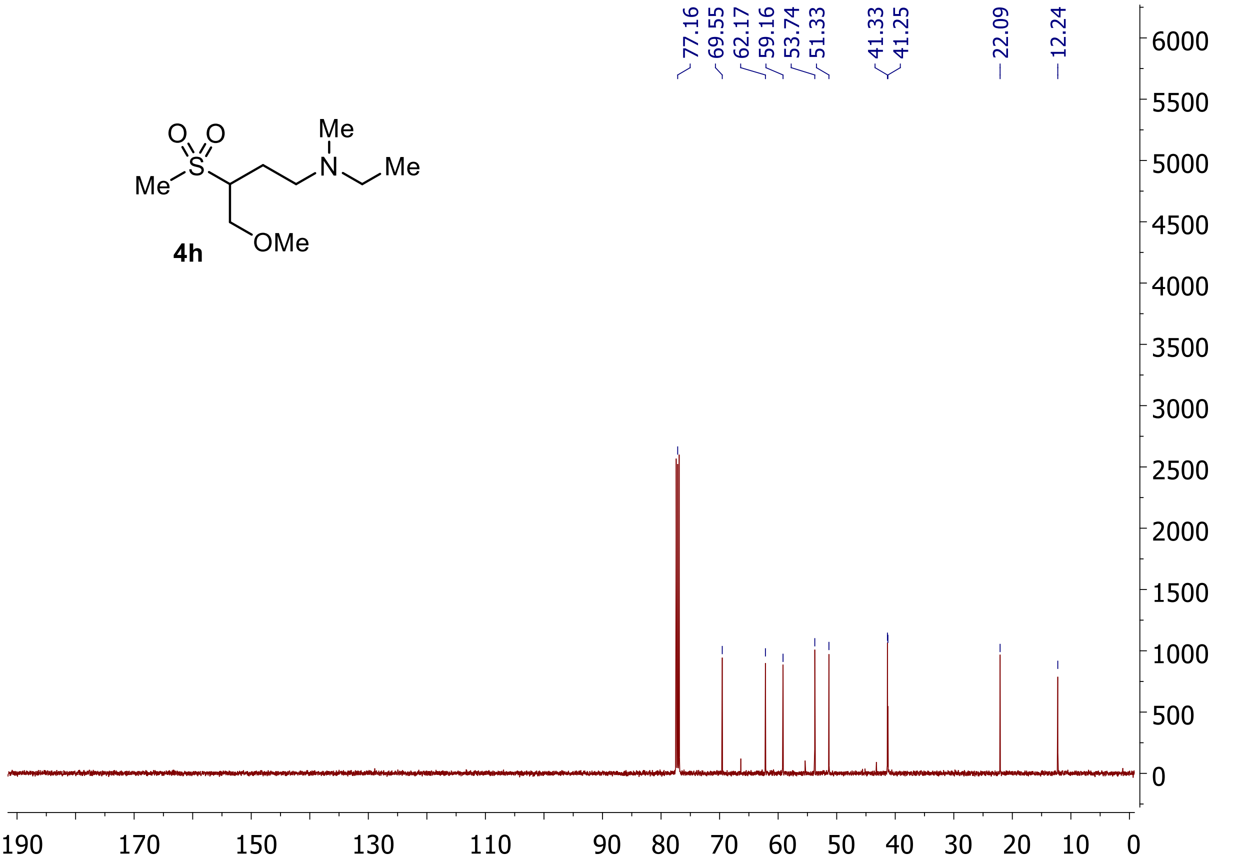

77.16 69.55 62.17 59.16 53.74 51.33 41.33 41.25 22.09 12.24
Me
O O
S
Me
N
Me
OMe
4h
190 170 150 130 110 90 80 70 60 50 40 30 20 10 0

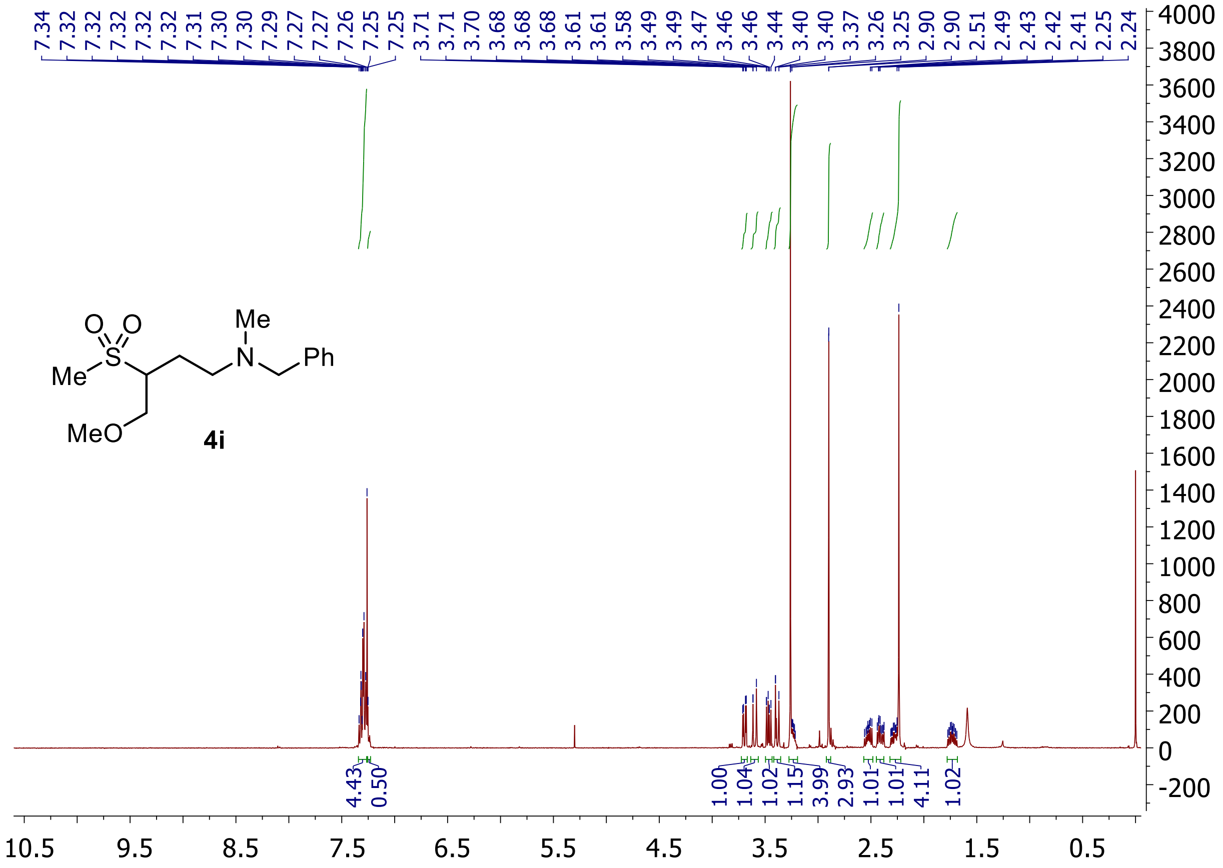

7.34 7.32 7.32 7.32 7.32 7.32 7.31 7.30 7.30 7.29 7.27 7.27 7.26 7.25 7.25
3.71 3.71 3.70 3.68 3.68 3.68 3.61 3.61 3.58 3.49 3.49 3.47 3.46 3.46 3.44 3.40 3.40 3.37 3.26 3.25 2.90 2.90 2.51 2.49 2.43 2.42 2.41 2.25 2.24
Me
N
Ph
O O
S
Me
MeO
4i
4.43 0.50
1.00 1.04 1.02 1.15 3.99 2.93 1.01 1.01 4.11 1.02
10.5 9.5 8.5 7.5 6.5 5.5 4.5 3.5 2.5 1.5 0.5
4000 3800 3600 3400 3200 3000 2800 2600 2400 2200 2000 1800 1600 1400 1200 1000 800 600 400 200 0 -200

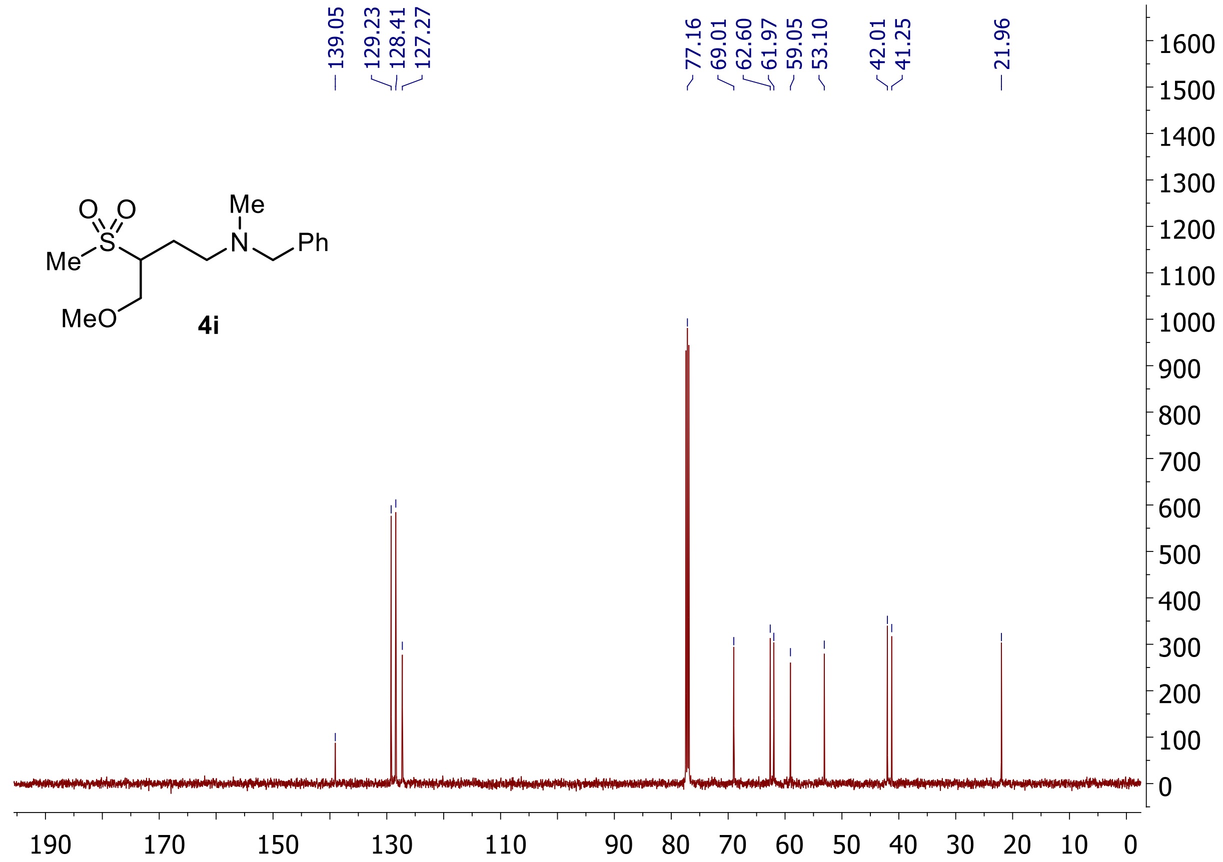

139.05
129.23 128.41 127.27
77.16 69.01 62.60 61.97 59.05 53.10
42.01 41.25
21.96
Me
N
Ph
O O
S
Me
MeO
4i
190 170 150 130 110 90 80 70 60 50 40 30 20 10 0
1600 1500 1400 1300 1200 1100 1000 900 800 700 600 500 400 300 200 100 0

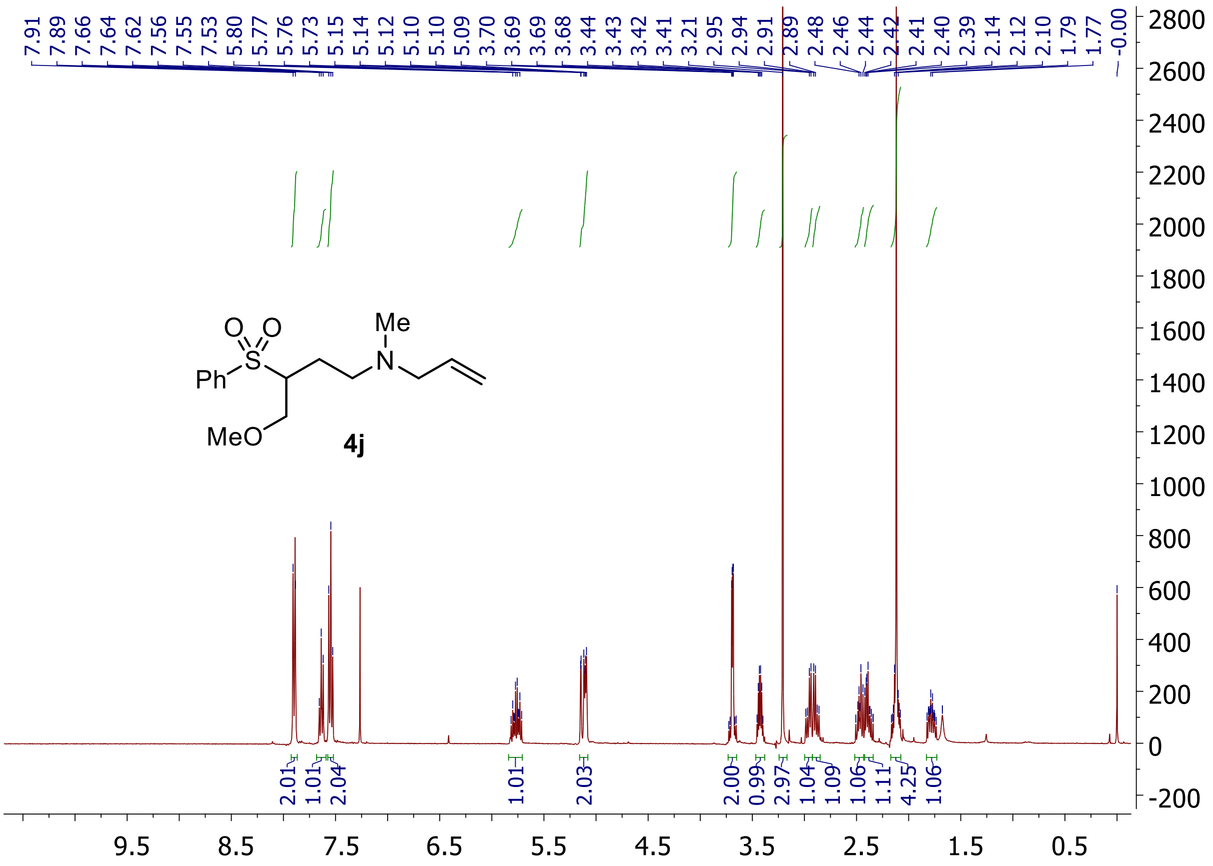

7.91 7.89 7.66 7.64 7.62 7.56 7.55 7.53 5.80 5.77 5.76 5.73 5.15 5.14 5.12 5.10 5.10 5.09 3.70 3.69 3.69 3.68 3.44 3.43 3.42 3.41 3.21 2.95 2.94 2.91 2.89 2.48 2.46 2.44 2.42 2.41 2.40 2.39 2.14 2.12 2.10 1.79 1.77 -0.00
Me
O O
S
N
Ph
MeO
4j
2.01 1.01 2.04 1.01 2.03 2.00 0.99 2.97 1.04 1.09 1.06 1.11 4.25 1.06
9.5 8.5 7.5 6.5 5.5 4.5 3.5 2.5 1.5 0.5
2800 2600 2400 2200 2000 1800 1600 1400 1200 1000 800 600 400 200 0 -200

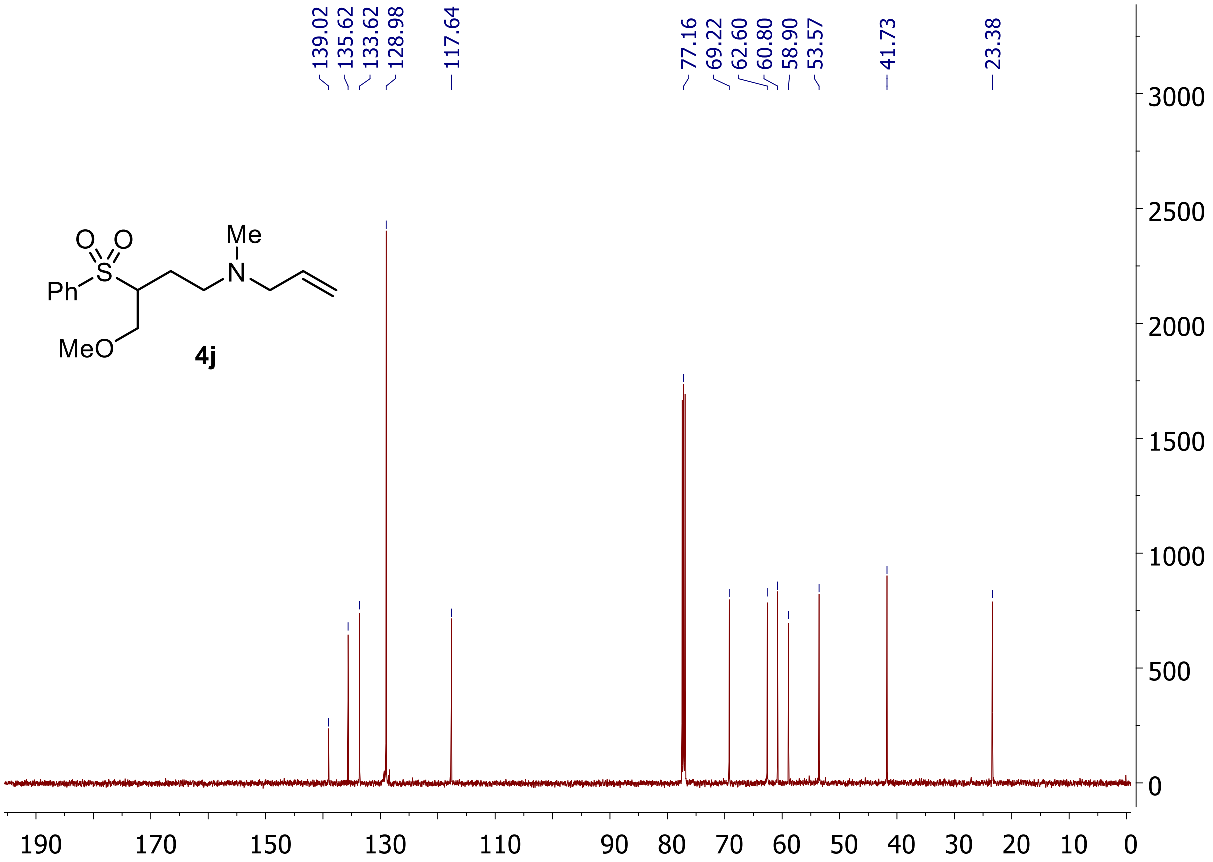

139.02 135.62 133.62 128.98 117.64 77.16 69.22 62.60 60.80 58.90 53.57 41.73 23.38
Me
O O
S
N
Ph
MeO
4j
190 170 150 130 110 90 80 70 60 50 40 30 20 10 0
3000 2500 2000 1500 1000 500 0

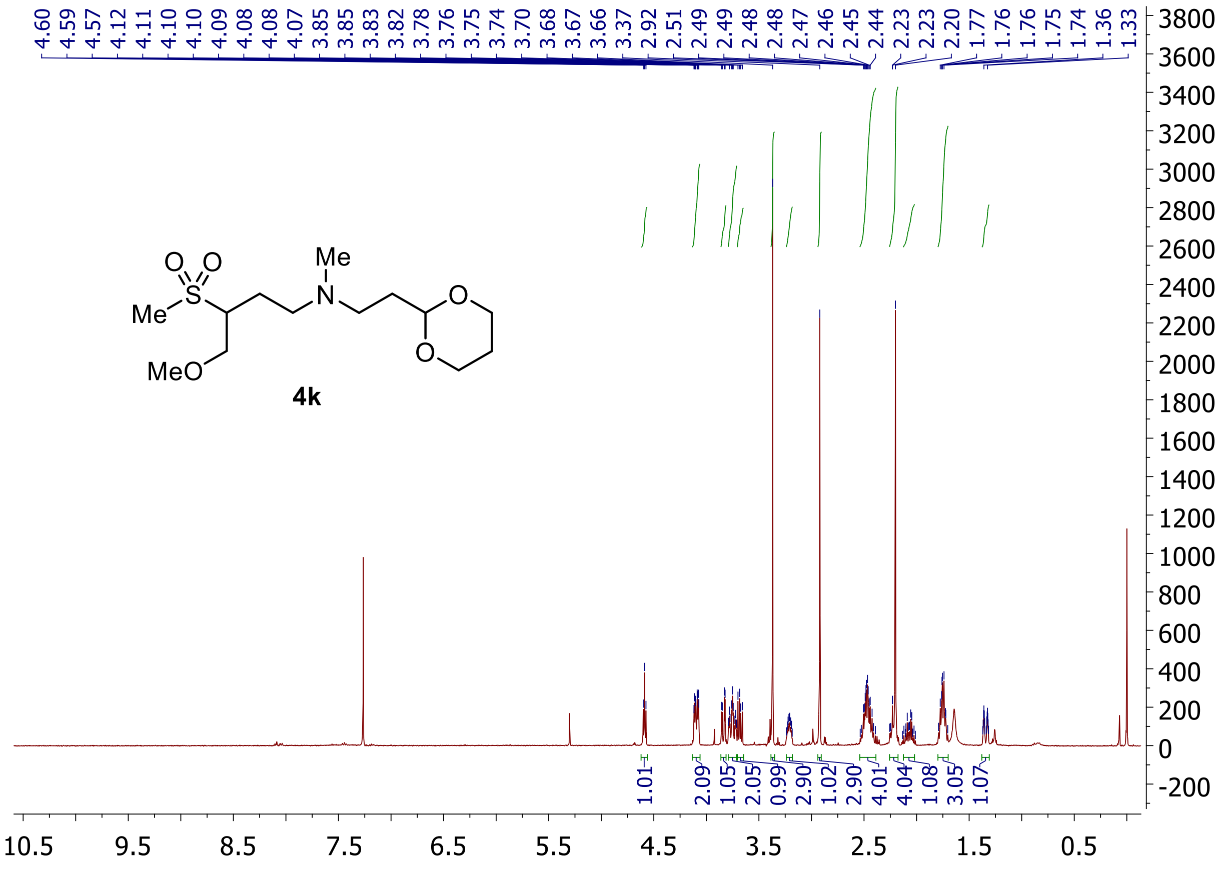

4.60
4.59
4.57
4.12
4.11
4.10
4.10
4.09
4.08
4.08
4.07
3.85
3.85
3.83
3.82
3.78
3.76
3.75
3.74
3.70
3.68
3.67
3.66
3.37
2.92
2.51
2.49
2.49
2.48
2.48
2.47
2.46
2.45
2.44
2.23
2.23
2.20
1.77
1.76
1.76
1.75
1.74
1.36
1.33
Me
O O
S
Me
N
O
O
MeO
4k
1.01
2.09
1.05
2.05
0.99
2.90
1.02
2.90
4.01
4.04
1.08
3.05
1.07

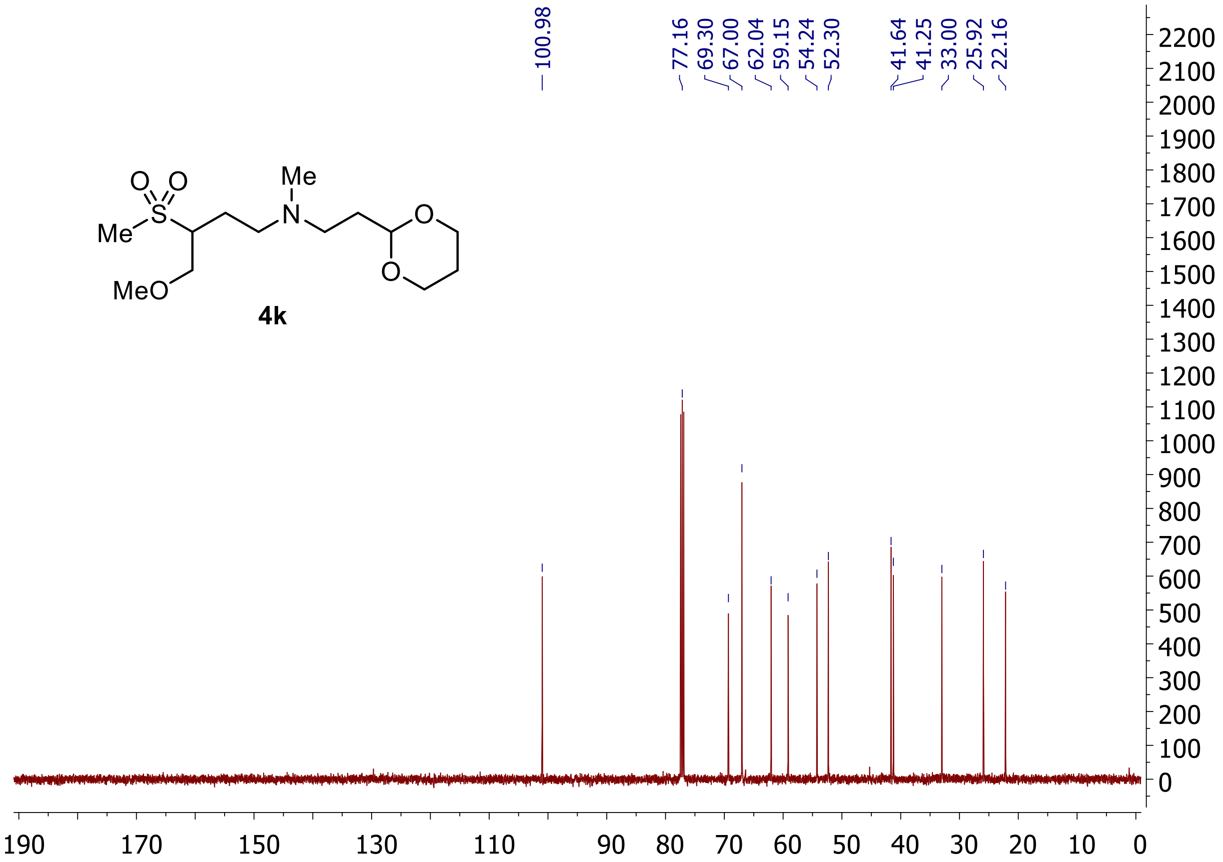

100.98
77.16
69.30
67.00
62.04
59.15
54.24
52.30
41.64
41.25
33.00
25.92
22.16
Me
O O
S
Me
N
O
O
MeO
4k

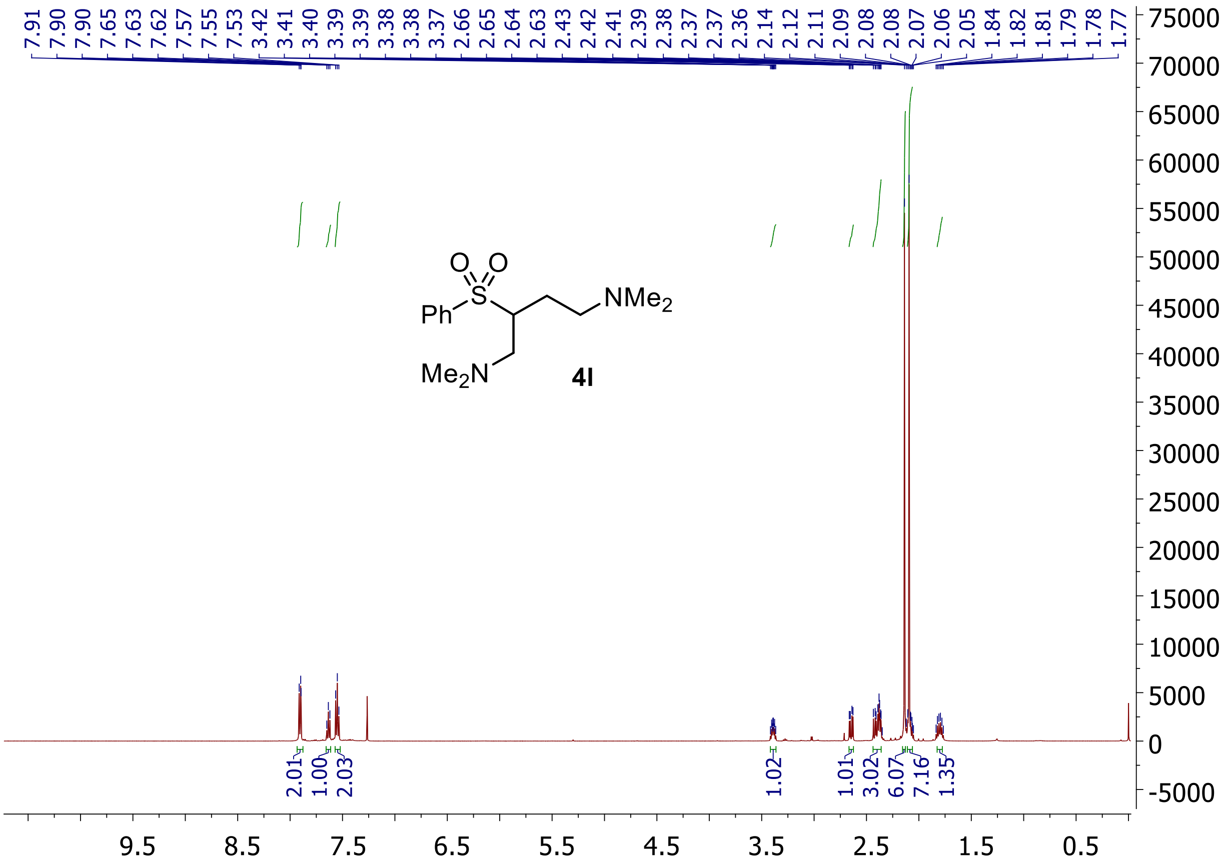
7.91
7.90
7.90
7.65
7.63
7.62
7.57
7.55
7.53
3.42
3.41
3.40
3.39
3.39
3.38
3.38
3.37
2.66
2.65
2.64
2.63
2.43
2.42
2.41
2.39
2.38
2.37
2.37
2.36
2.14
2.12
2.11
2.09
2.08
2.08
2.07
2.06
2.05
1.84
1.82
1.81
1.79
1.78
1.77
Ph
NMe2
Me2N
4l
2.01
1.00
2.03
1.02
1.01
3.02
6.07
7.16
1.35
9.5
8.5
7.5
6.5
5.5
4.5
3.5
2.5
1.5
0.5

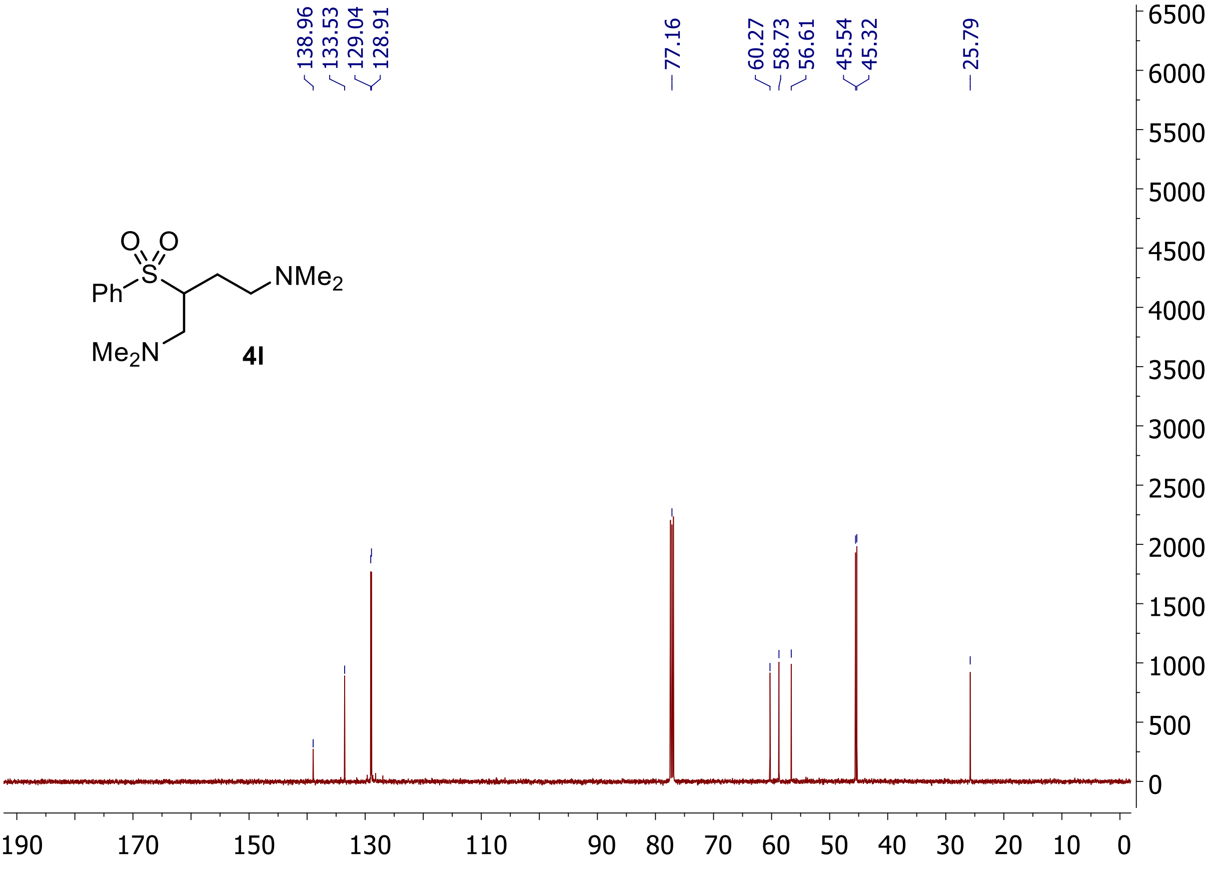
138.96
133.53
129.04
128.91
77.16
60.27
58.73
56.61
45.54
45.32
25.79
Ph
NMe2
Me2N
4l
190
170
150
130
110
90
80
70
60
50
40
30
20
10
0

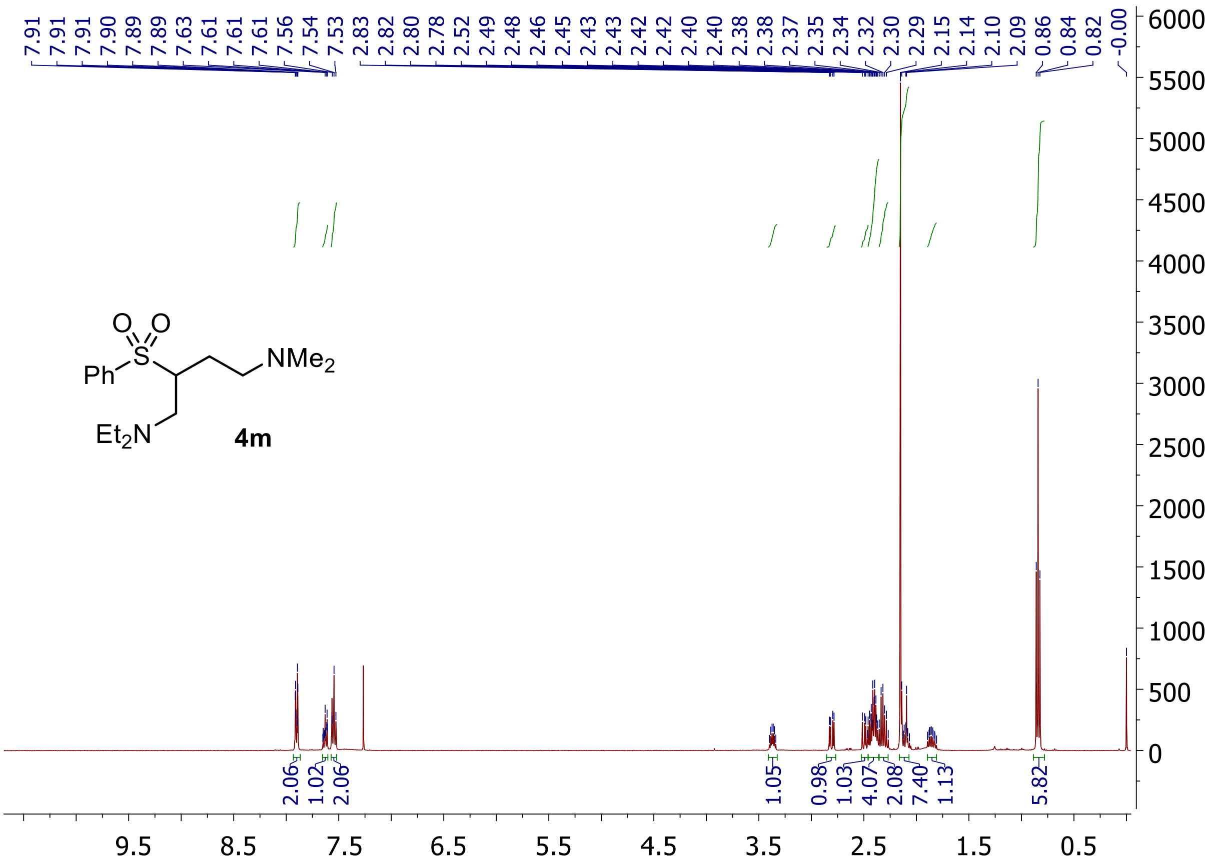
7.91 7.91 7.91 7.90 7.89 7.89 7.63 7.61 7.61 7.61 7.56 7.54 7.53 2.83 2.82 2.80 2.78 2.52 2.49 2.48 2.46 2.45 2.43 2.43 2.42 2.42 2.40 2.40 2.38 2.38 2.37 2.35 2.34 2.32 2.30 2.29 2.15 2.14 2.10 2.09 0.86 0.84 0.82 -0.00
Ph
S
O O
NMe2
Et2N
4m
2.06 1.02 2.06 1.05 0.98 1.03 4.07 2.08 7.40 1.13 5.82
9.5 8.5 7.5 6.5 5.5 4.5 3.5 2.5 1.5 0.5
6000 5500 5000 4500 4000 3500 3000 2500 2000 1500 1000 500 0

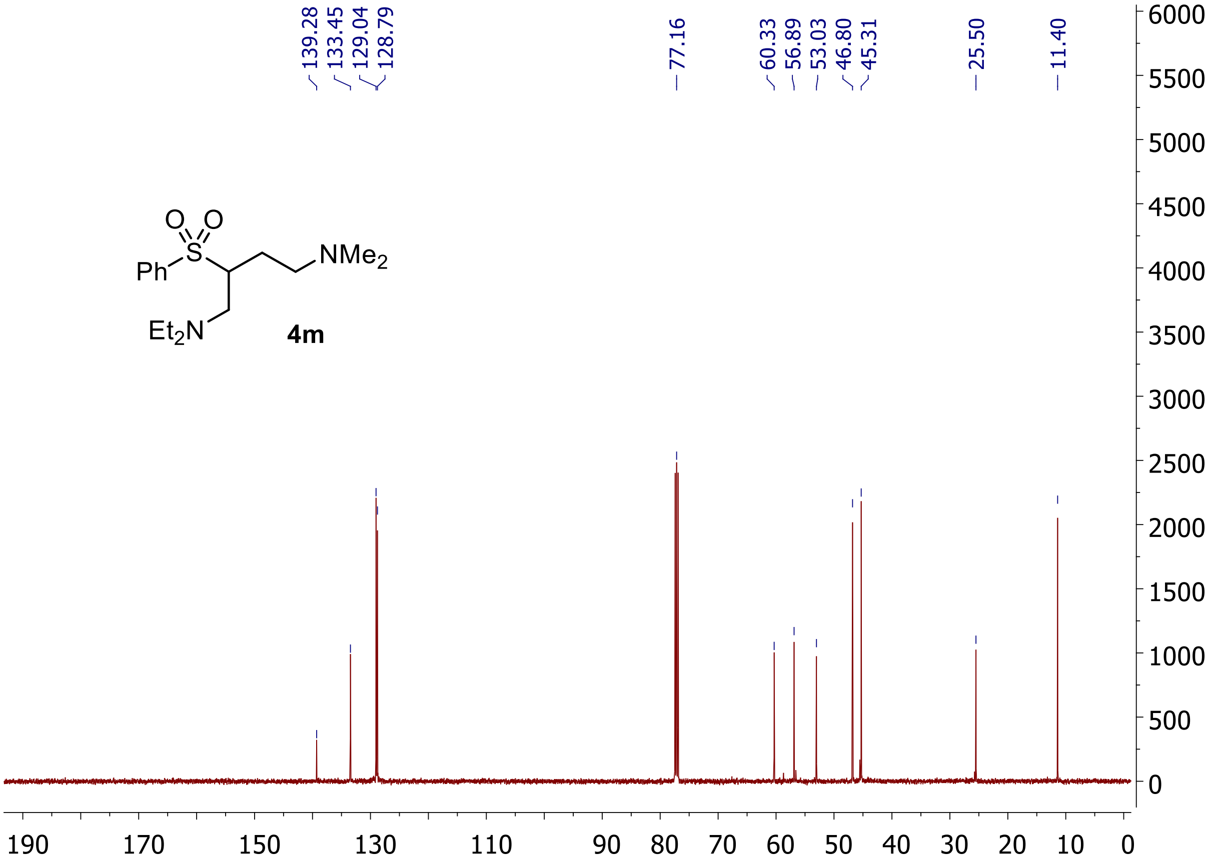
139.28 133.45 129.04 128.79 77.16 60.33 56.89 53.03 46.80 45.31 25.50 11.40
Ph
S
O O
NMe2
Et2N
4m
190 170 150 130 110 90 80 70 60 50 40 30 20 10 0
6000 5500 5000 4500 4000 3500 3000 2500 2000 1500 1000 500 0

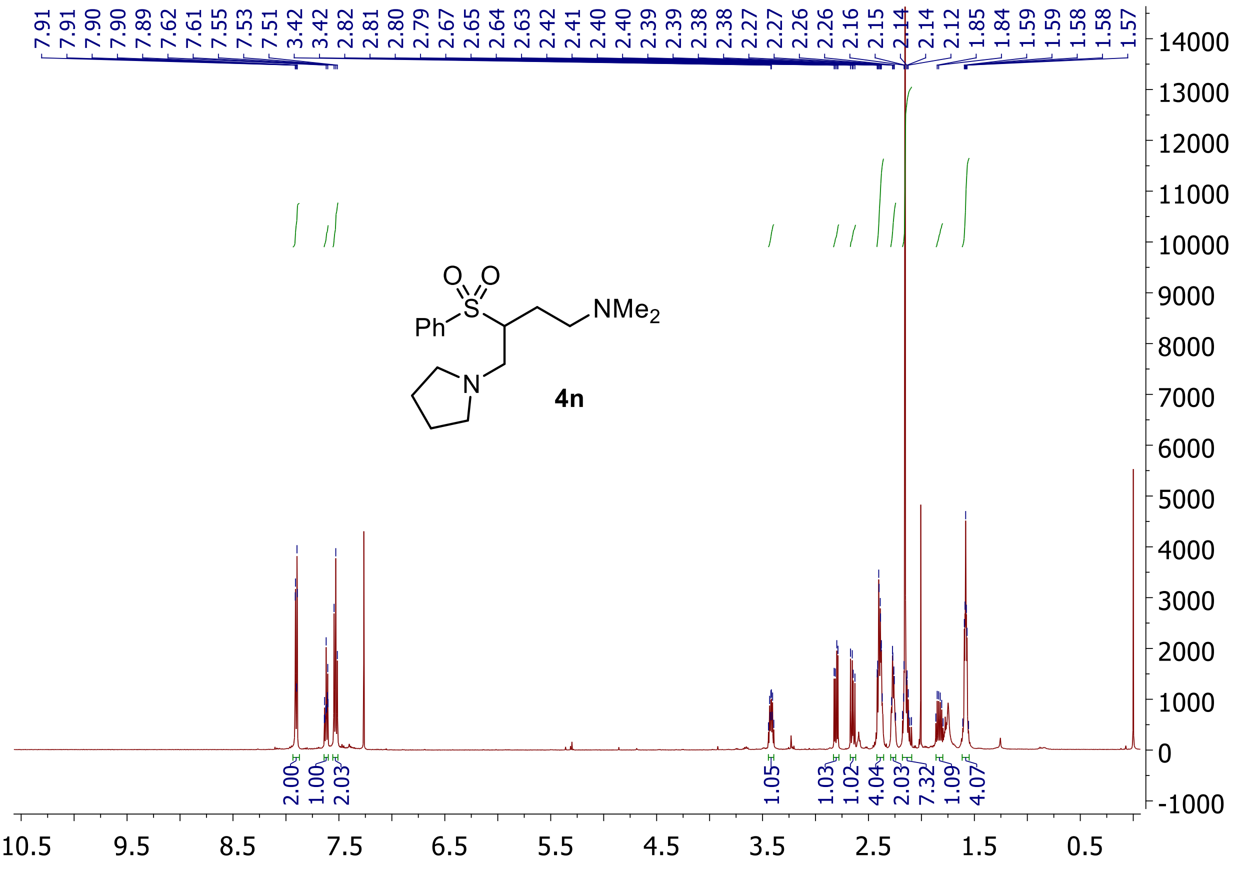
7.91
7.91
7.90
7.90
7.89
7.62
7.61
7.55
7.53
7.51
3.42
3.42
2.82
2.81
2.80
2.79
2.67
2.65
2.64
2.63
2.42
2.41
2.40
2.40
2.39
2.39
2.38
2.38
2.27
2.27
2.26
2.26
2.16
2.15
2.14
2.14
2.12
1.85
1.84
1.59
1.59
1.58
1.58
1.57
Ph
S
O
O
NMe2
N
4n
2.00
1.00
2.03
1.05
1.03
1.02
4.04
2.03
7.32
1.09
4.07
10.5
9.5
8.5
7.5
6.5
5.5
4.5
3.5
2.5
1.5
0.5

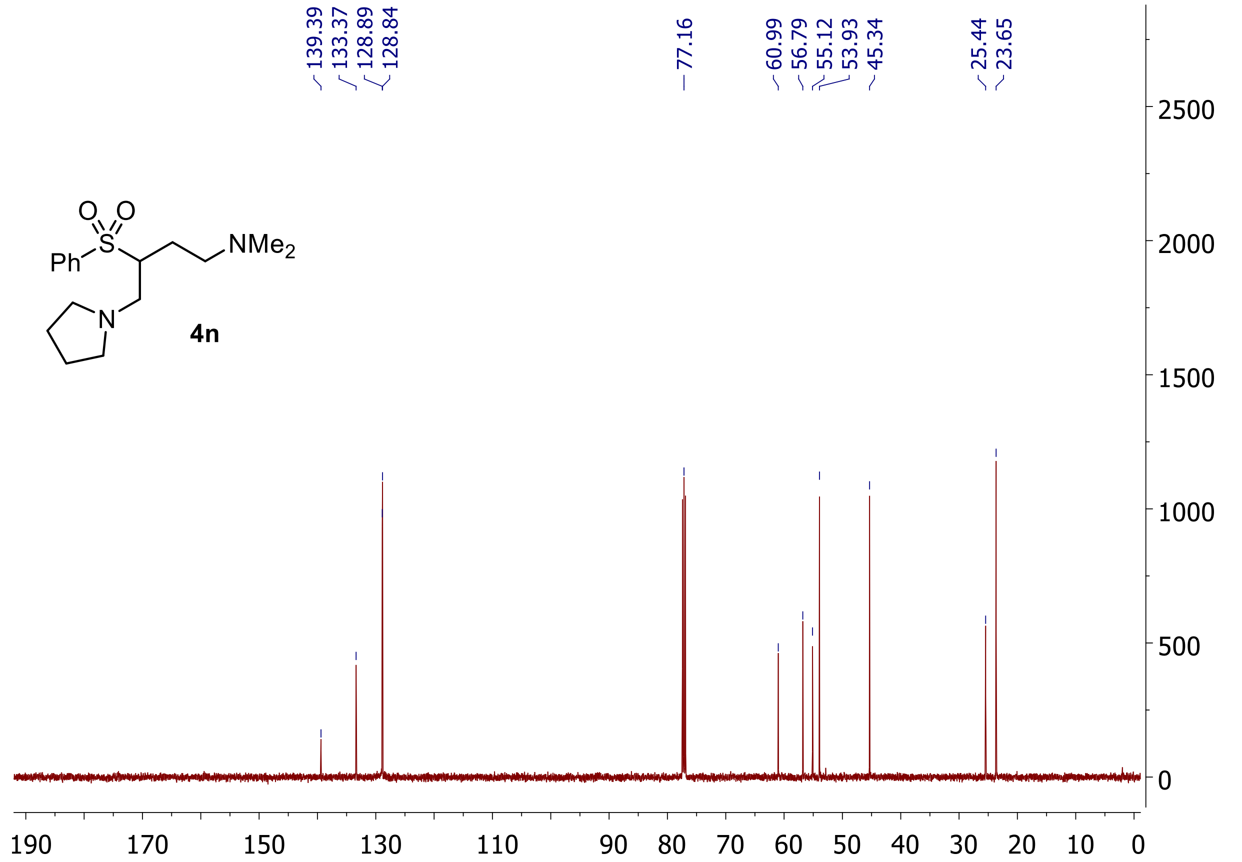
139.39
133.37
128.89
128.84
77.16
60.99
56.79
55.12
53.93
45.34
25.44
23.65
Ph
S
O
O
NMe2
N
4n
190
170
150
130
110
90
80
70
60
50
40
30
20
10
0

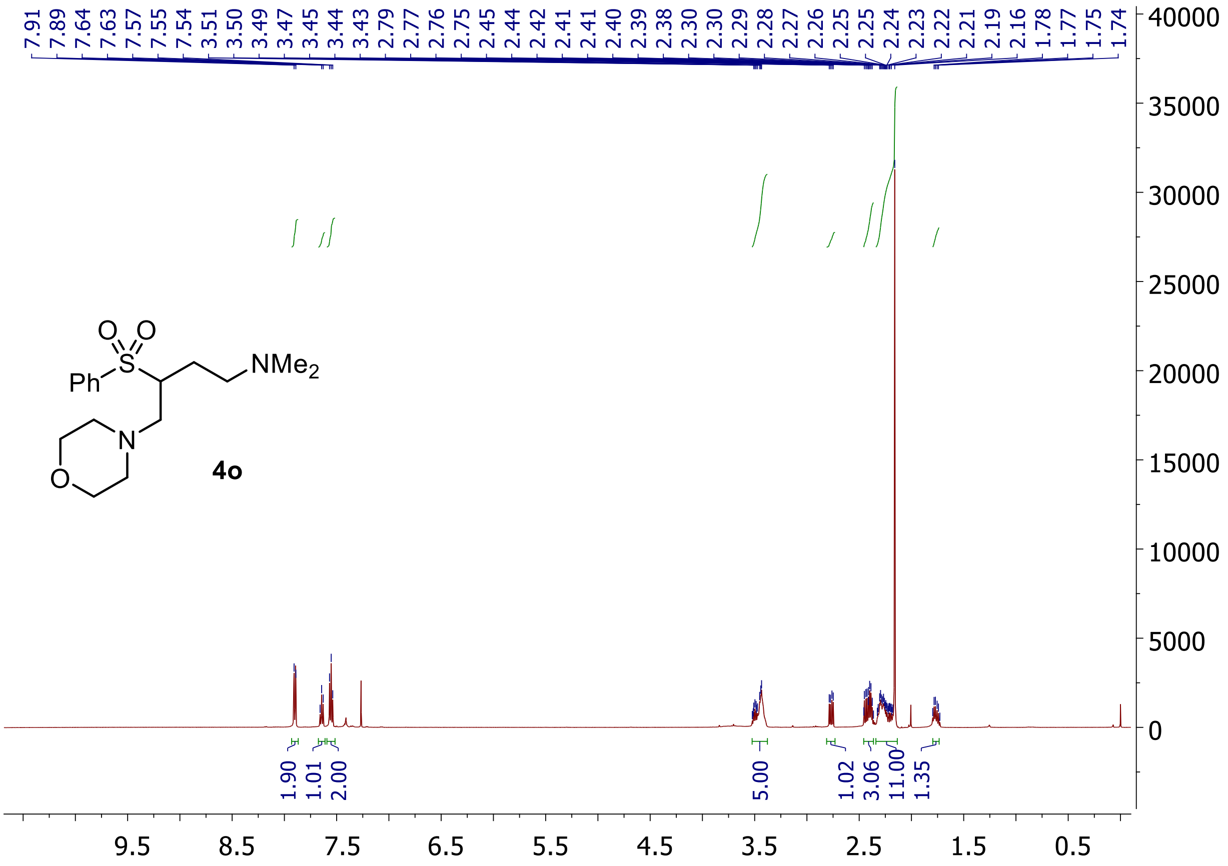
7.91 7.89 7.64 7.63 7.57 7.55 7.54 3.51 3.50 3.49 3.47 3.45 3.44 3.43 2.79 2.77 2.76 2.75 2.45 2.44 2.42 2.41 2.41 2.40 2.39 2.38 2.30 2.30 2.29 2.28 2.27 2.26 2.25 2.25 2.24 2.23 2.22 2.21 2.19 2.16 1.78 1.77 1.75 1.74
Ph
S
O O
NMe2
N
O
4o
1.90 1.01 2.00 5.00 1.02 3.06 11.00 1.35
9.5 8.5 7.5 6.5 5.5 4.5 3.5 2.5 1.5 0.5
40000 35000 30000 25000 20000 15000 10000 5000 0

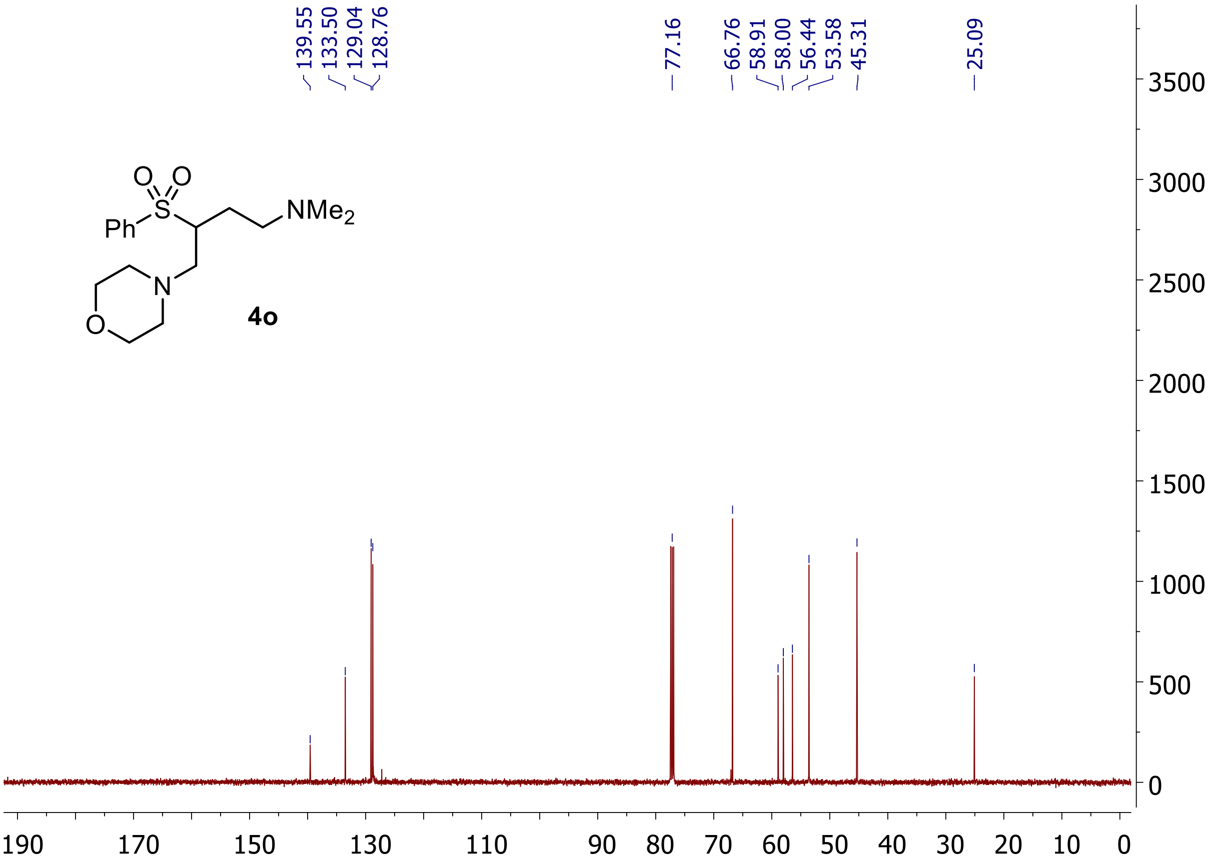
139.55 133.50 129.04 128.76 77.16 66.76 58.91 58.00 56.44 53.58 45.31 25.09
Ph
S
O O
NMe2
N
O
4o
190 170 150 130 110 90 80 70 60 50 40 30 20 10 0
3500 3000 2500 2000 1500 1000 500 0

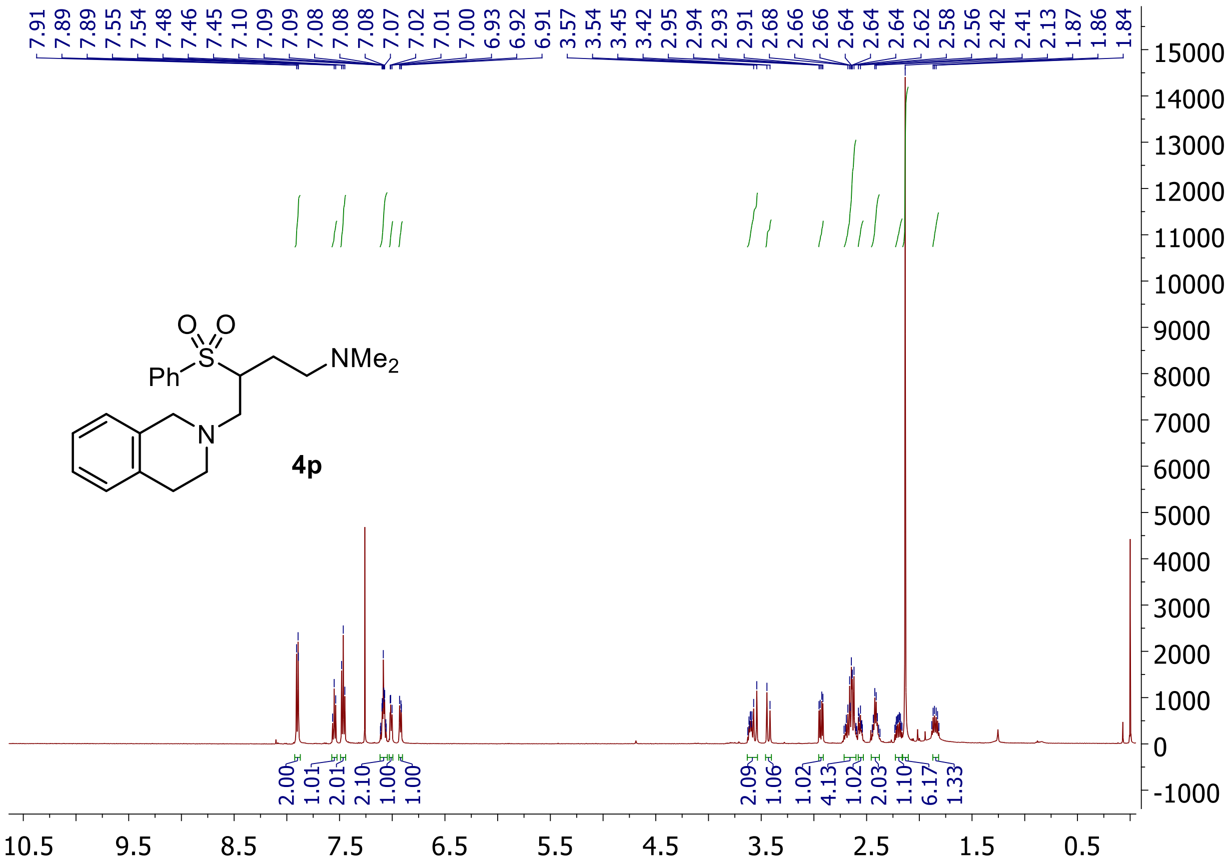
7.91 7.89 7.89 7.55 7.54 7.48 7.46 7.45 7.10 7.09 7.09 7.08 7.08 7.07 7.02 7.01 7.00 6.93 6.92 6.91 3.57 3.54 3.45 3.42 2.95 2.94 2.93 2.91 2.68 2.66 2.66 2.64 2.64 2.64 2.62 2.58 2.56 2.42 2.41 2.13 1.87 1.86 1.84
Ph
S
O O
NMe2
N
4p
2.00 1.01 2.01 2.10 1.00 1.00 2.09 1.06 1.02 4.13 1.02 2.03 1.10 6.17 1.33
10.5 9.5 8.5 7.5 6.5 5.5 4.5 3.5 2.5 1.5 0.5

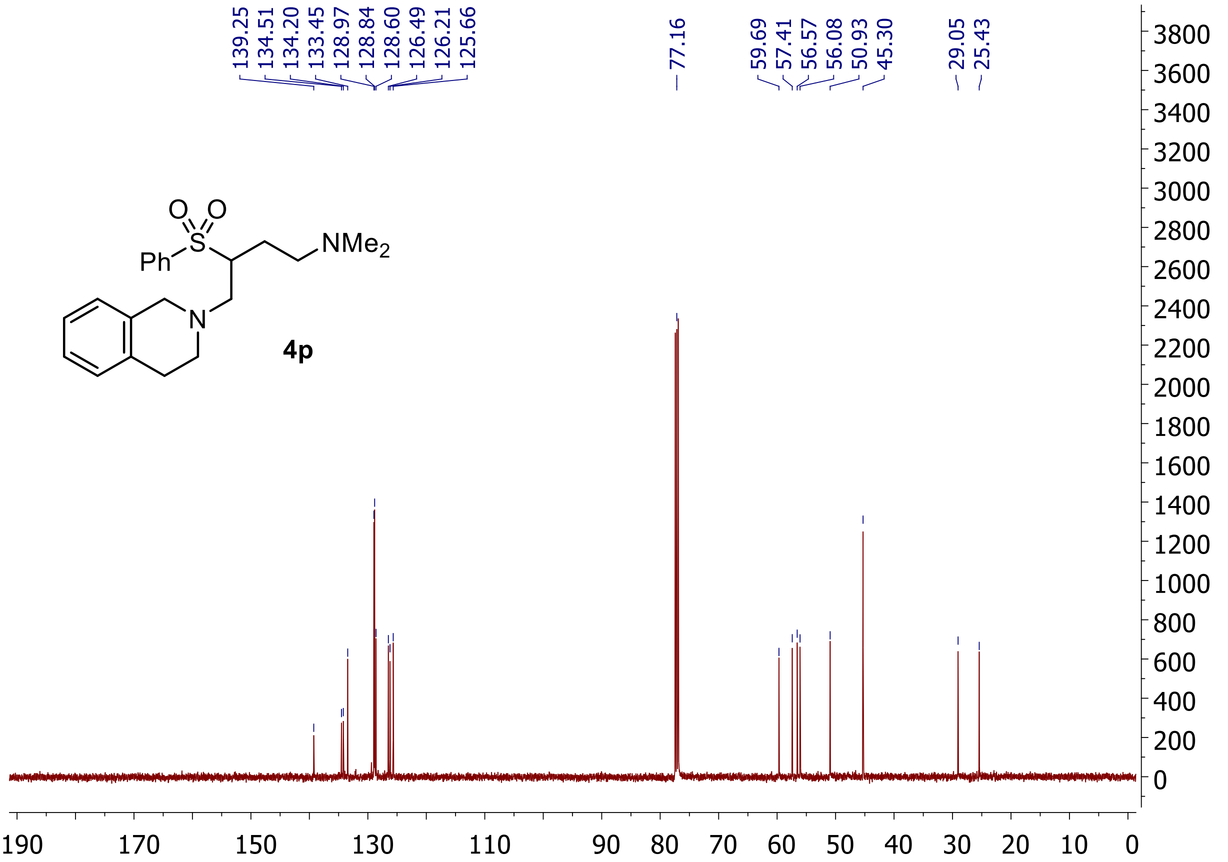
139.25 134.51 134.20 133.45 128.97 128.84 128.60 126.49 126.21 125.66
77.16
59.69 57.41 56.57 56.08 50.93 45.30
29.05 25.43
Ph
S
O O
NMe2
N
4p
190 170 150 130 110 90 80 70 60 50 40 30 20 10 0

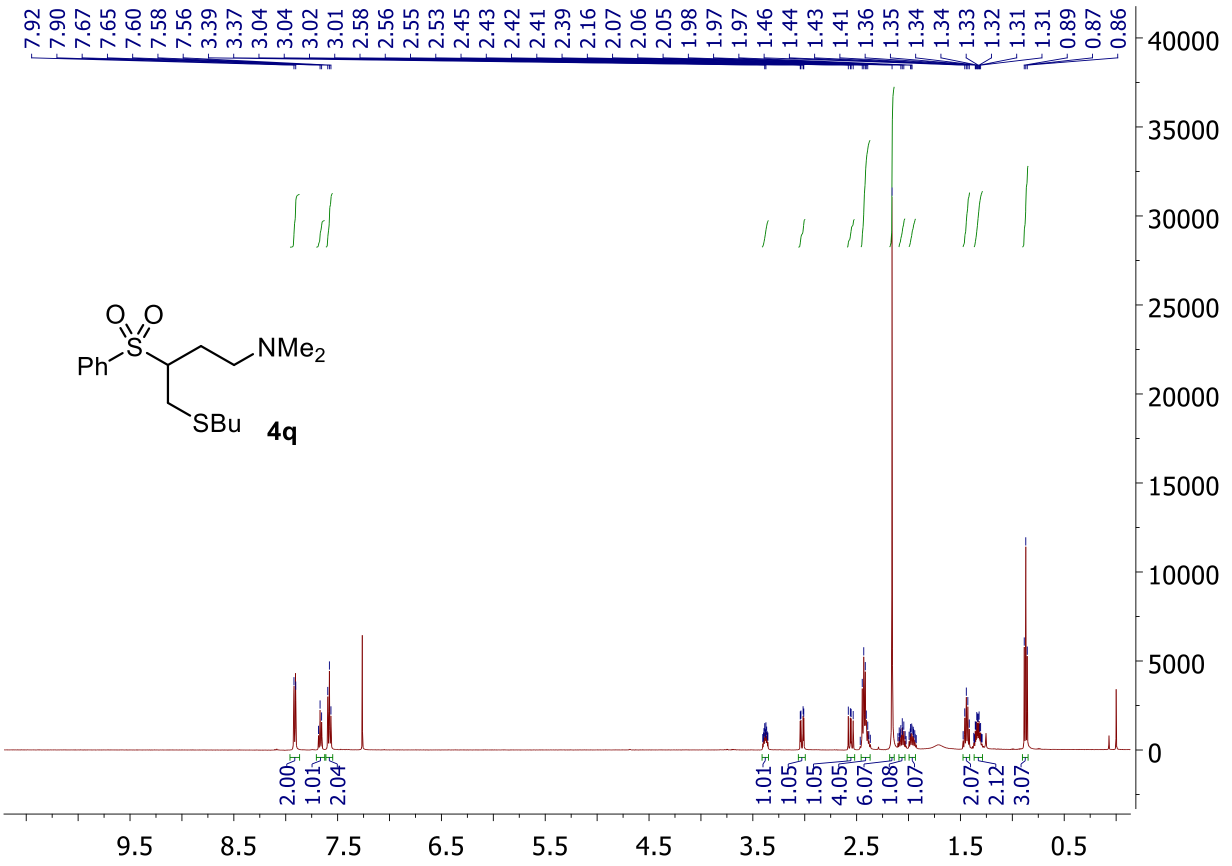
7.92 7.90 7.67 7.65 7.60 7.58 7.56 3.39 3.37 3.04 3.04 3.02 3.01 2.58 2.56 2.55 2.53 2.45 2.43 2.42 2.41 2.39 2.16 2.07 2.06 2.05 1.98 1.97 1.97 1.46 1.44 1.43 1.41 1.36 1.35 1.34 1.34 1.33 1.32 1.31 1.31 0.89 0.87 0.86
Ph
S
O O
NMe2
SBu
4q
2.00 1.01 2.04 1.01 1.05 1.05 4.05 6.07 1.08 1.07 2.07 2.12 3.07

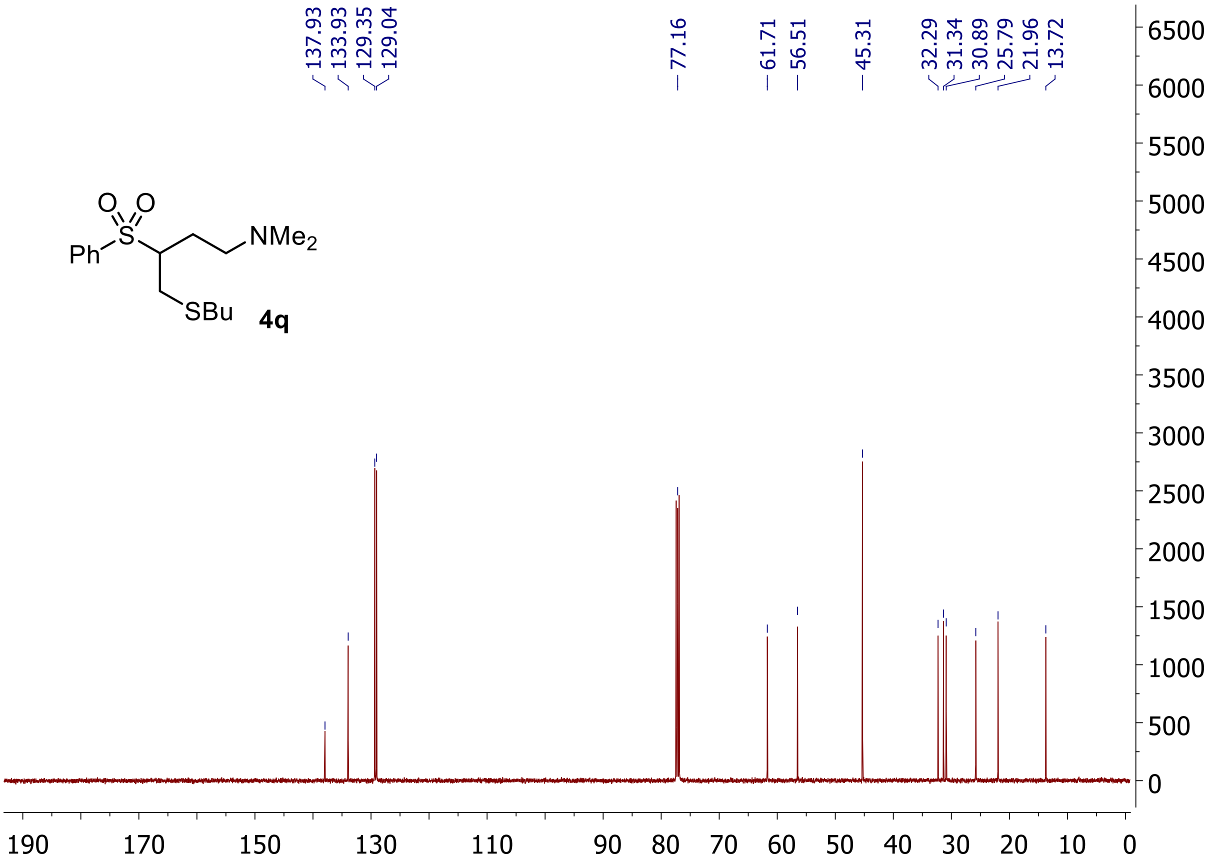
137.93 133.93 129.35 129.04
77.16
61.71 56.51
45.31
32.29 31.34 30.89 25.79 21.96 13.72
Ph
S
O O
NMe2
SBu
4q

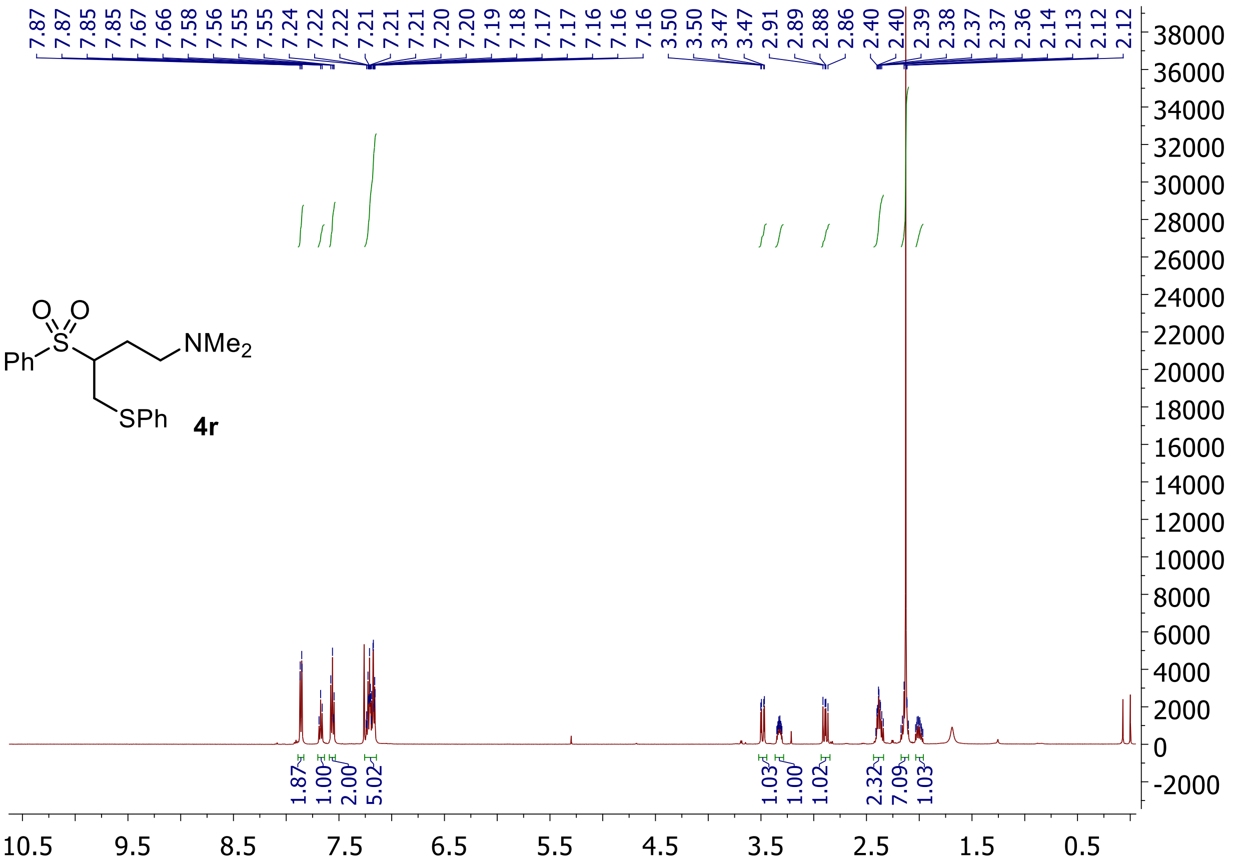
7.87 7.87 7.85 7.85 7.67 7.66 7.58 7.56 7.55 7.55 7.24 7.22 7.22 7.21 7.21 7.21 7.20 7.20 7.19 7.18 7.17 7.17 7.16 7.16 7.16
3.50 3.50 3.47 3.47 2.91 2.89 2.88 2.86 2.40 2.40 2.39 2.38 2.37 2.37 2.36 2.14 2.13 2.12 2.12
Ph
S
O O
NMe2
SPh
4r
1.87 1.00 2.00 5.02 1.03 1.00 1.02 2.32 7.09 1.03
10.5 9.5 8.5 7.5 6.5 5.5 4.5 3.5 2.5 1.5 0.5
38000 36000 34000 32000 30000 28000 26000 24000 22000 20000 18000 16000 14000 12000 10000 8000 6000 4000 2000 0 -2000

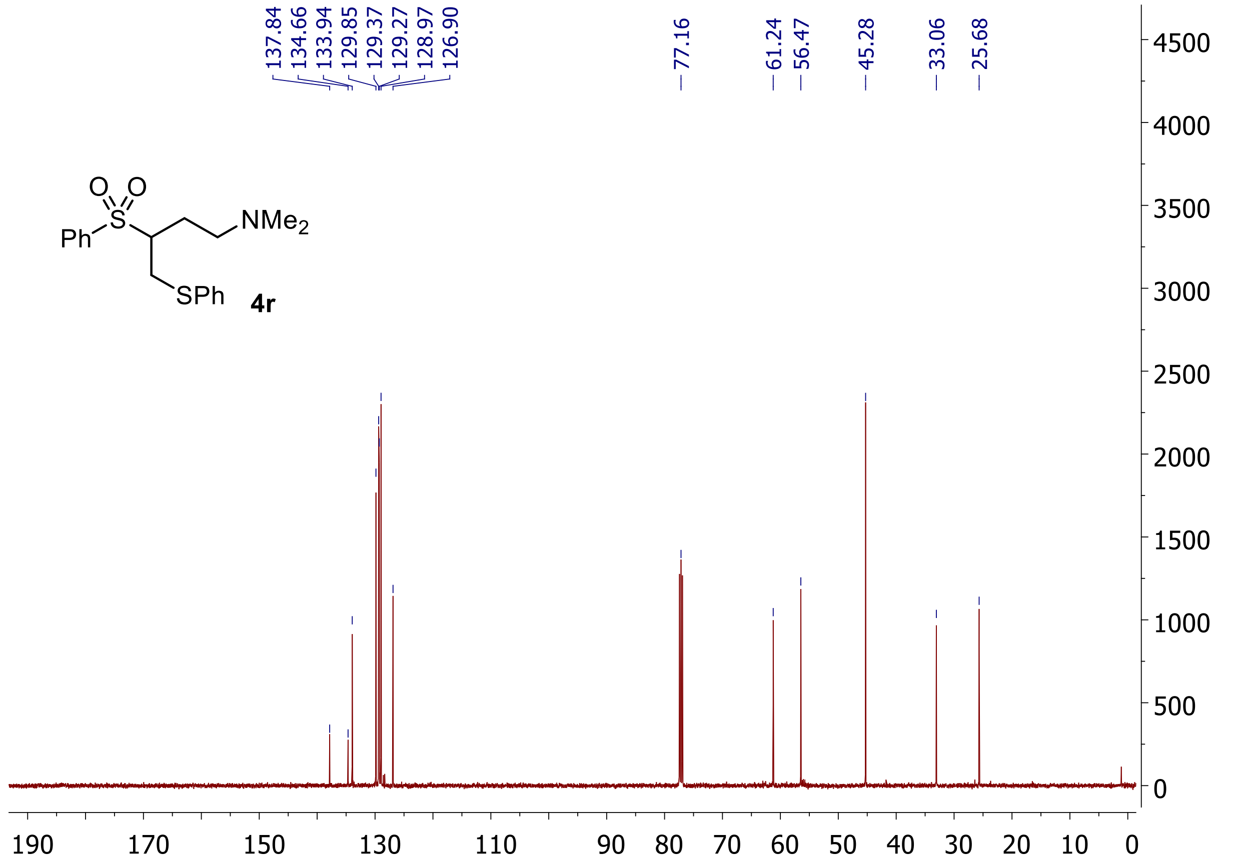
137.84 134.66 133.94 129.85 129.37 129.27 128.97 126.90
77.16
61.24
56.47
45.28
33.06
25.68
Ph
S
O O
NMe2
SPh
4r
190 170 150 130 110 90 80 70 60 50 40 30 20 10 0
4500 4000 3500 3000 2500 2000 1500 1000 500 0

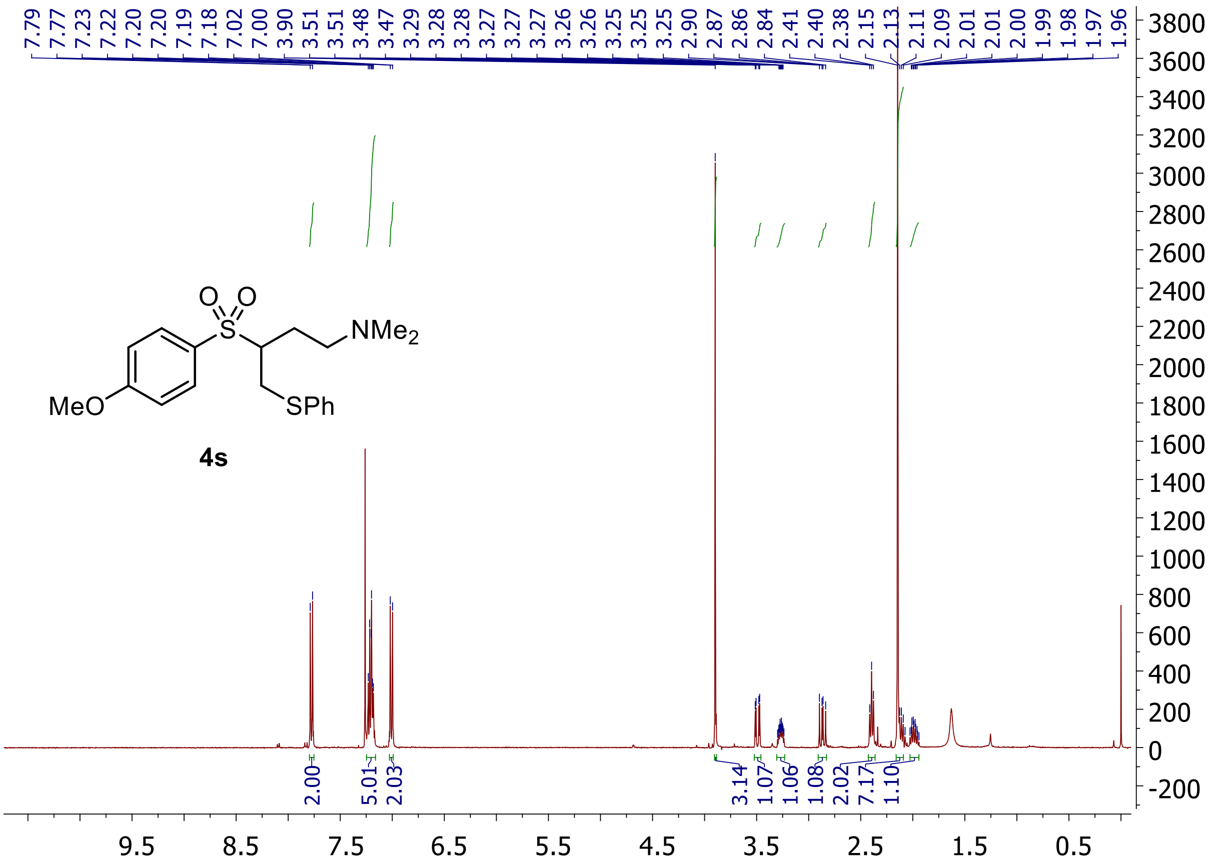
7.79 7.77 7.23 7.22 7.20 7.20 7.19 7.18 7.02 7.00 3.90 3.51 3.51 3.48 3.47 3.29 3.28 3.28 3.27 3.27 3.27 3.26 3.26 3.25 3.25 2.90 2.87 2.86 2.84 2.41 2.40 2.38 2.15 2.13 2.11 2.09 2.01 2.00 1.99 1.98 1.97 1.96
2.00 5.01 2.03 3.14 1.07 1.06 1.08 2.02 7.17 1.10
MeO
SPh
NMe_2
4s

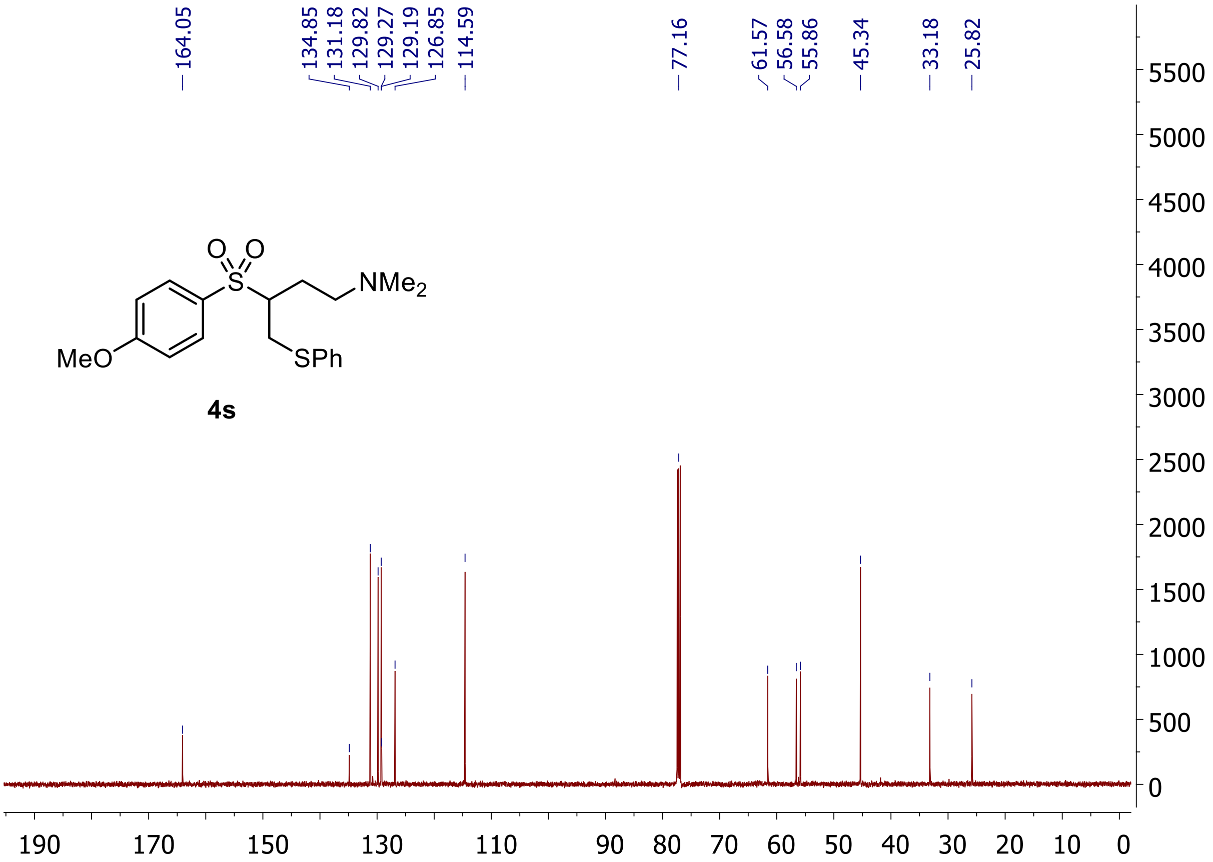
164.05 134.85 131.18 129.82 129.27 129.19 126.85 114.59 77.16 61.57 56.58 55.86 45.34 33.18 25.82
MeO
SPh
NMe_2
4s

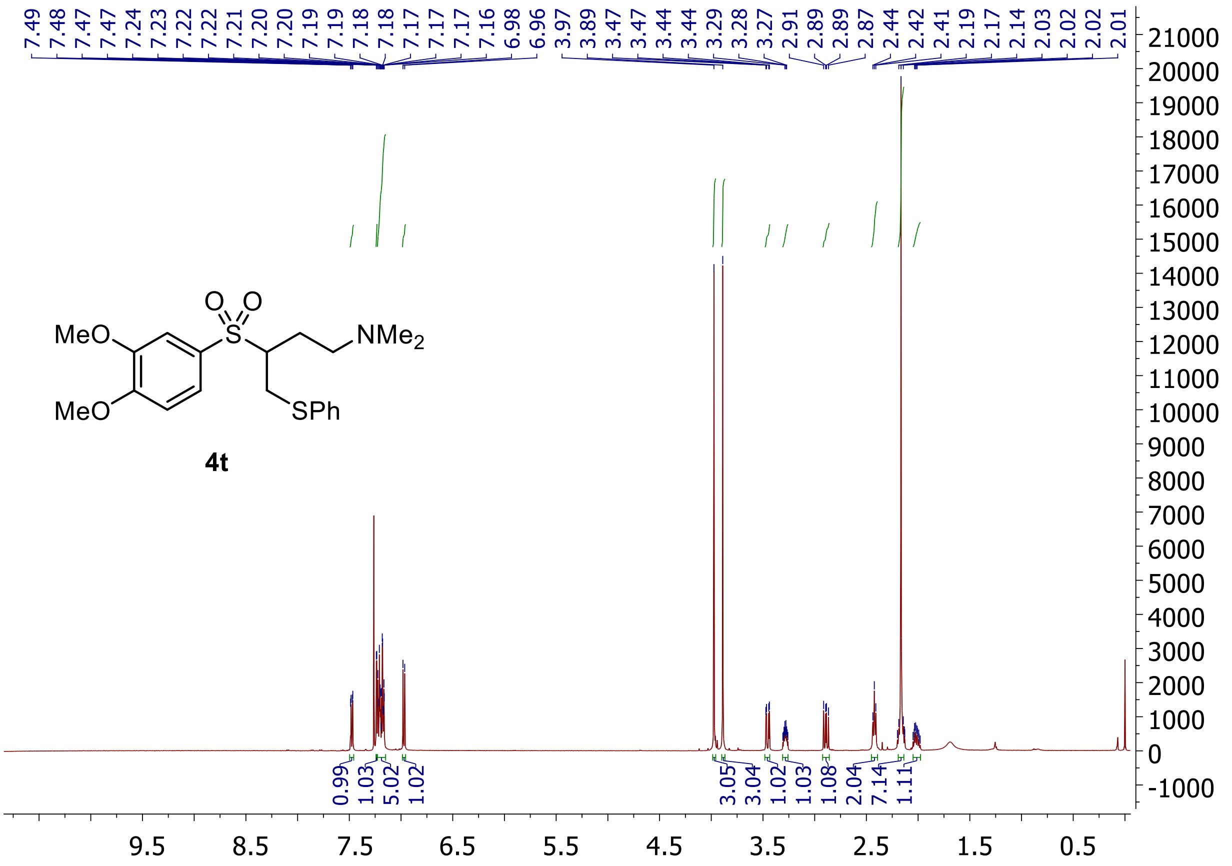
7.49 7.48 7.47 7.47 7.24 7.23 7.22 7.22 7.21 7.20 7.20 7.19 7.19 7.18 7.18 7.17 7.17 7.17 7.16 6.98 6.96 3.97 3.89 3.47 3.47 3.44 3.44 3.29 3.28 3.27 2.91 2.89 2.89 2.87 2.44 2.42 2.41 2.19 2.17 2.14 2.03 2.02 2.02 2.01
MeO
MeO
NMe2
SPh
4t
0.99 1.03 5.02 1.02 3.05 3.04 1.02 1.03 1.08 2.04 7.14 1.11
9.5 8.5 7.5 6.5 5.5 4.5 3.5 2.5 1.5 0.5
21000 20000 19000 18000 17000 16000 15000 14000 13000 12000 11000 10000 9000 8000 7000 6000 5000 4000 3000 2000 1000 0 -1000

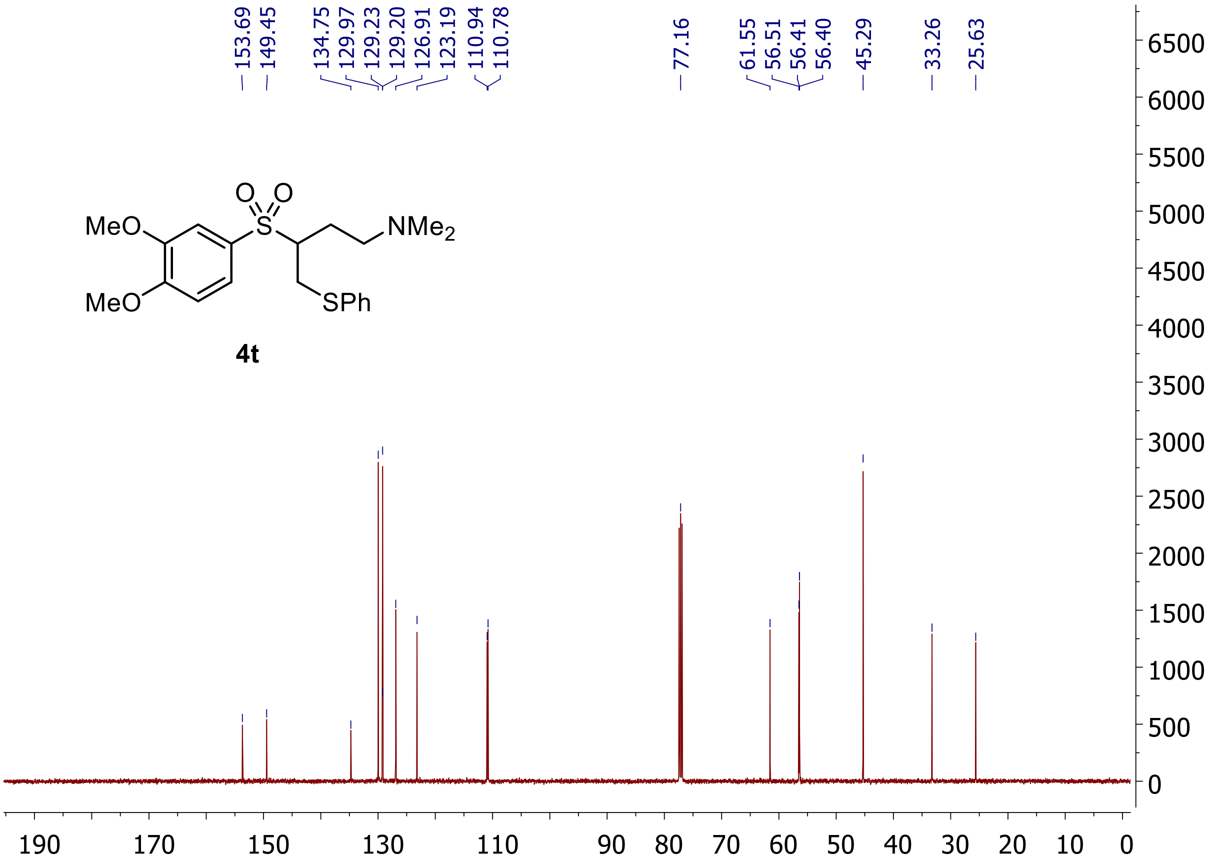
153.69 149.45 134.75 129.97 129.23 129.20 126.91 123.19 110.94 110.78 77.16 61.55 56.51 56.41 56.40 45.29 33.26 25.63
MeO
MeO
NMe2
SPh
4t
190 170 150 130 110 90 80 70 60 50 40 30 20 10 0
6500 6000 5500 5000 4500 4000 3500 3000 2500 2000 1500 1000 500 0

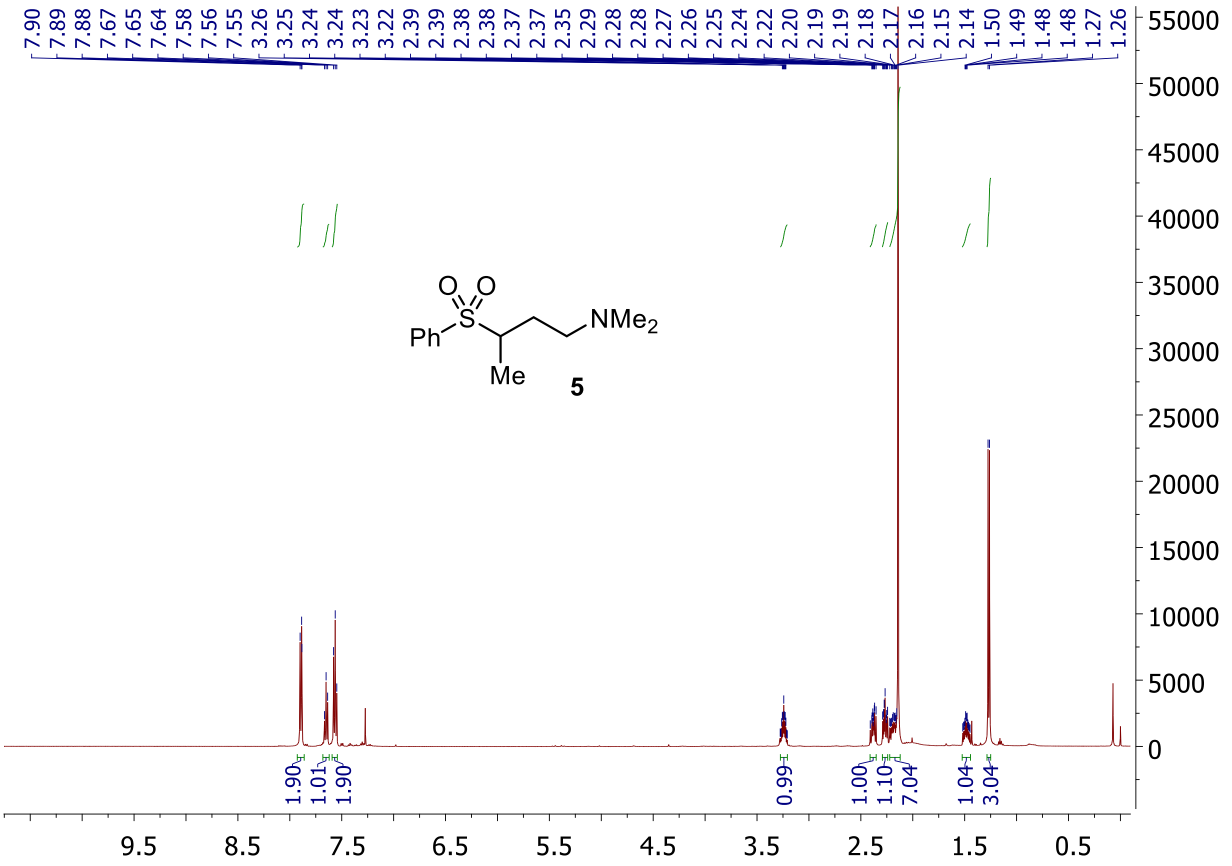

7.90
7.89
7.88
7.67
7.65
7.64
7.58
7.56
7.55
3.26
3.25
3.24
3.24
3.23
3.22
2.39
2.39
2.38
2.38
2.37
2.37
2.35
2.29
2.28
2.28
2.27
2.26
2.25
2.24
2.22
2.20
2.19
2.19
2.18
2.17
2.16
2.15
2.14
1.50
1.49
1.48
1.48
1.27
1.26
Ph
NMe2
Me
5
1.90
1.01
1.90
0.99
1.00
1.10
7.04
1.04
3.04
9.5
8.5
7.5
6.5
5.5
4.5
3.5
2.5
1.5
0.5
55000
50000
45000
40000
35000
30000
25000
20000
15000
10000
5000
0

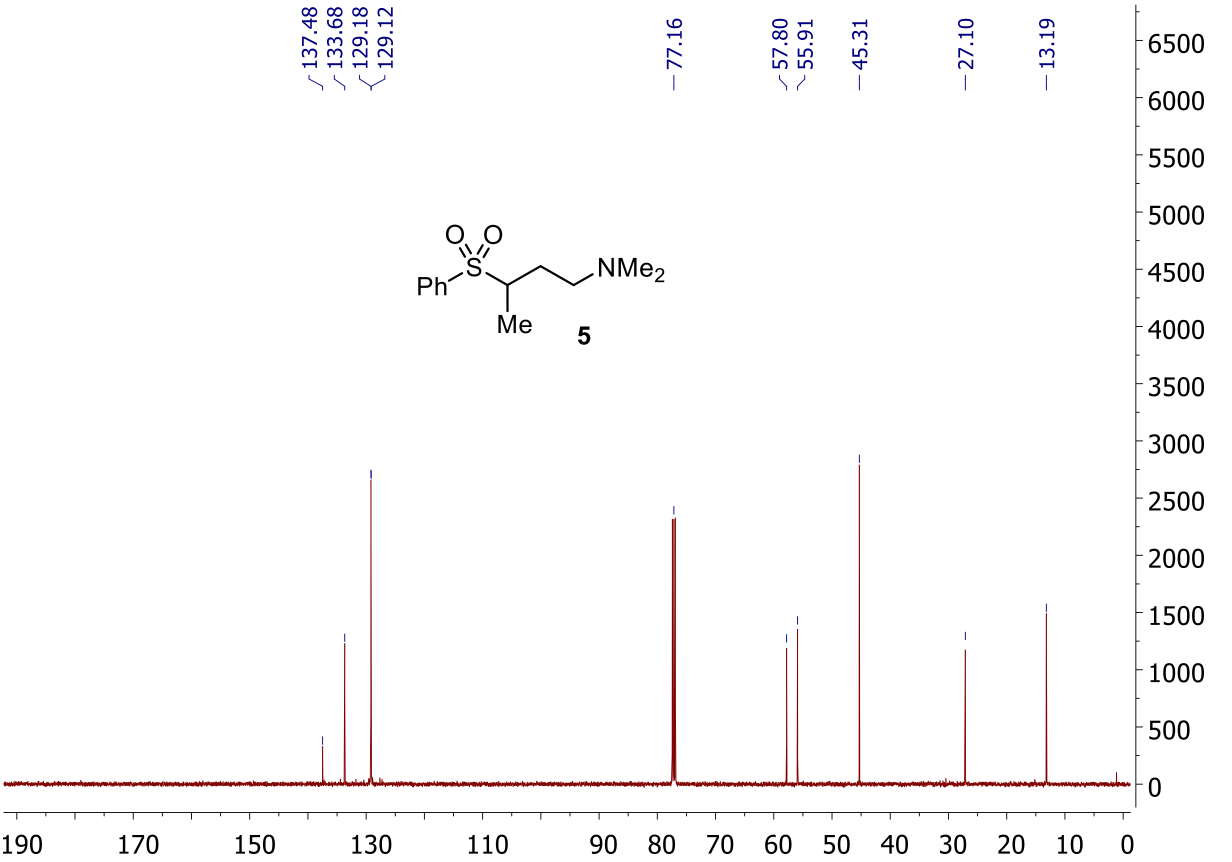

137.48
133.68
129.18
129.12
77.16
57.80
55.91
45.31
27.10
13.19
Ph
NMe2
Me
5
190
170
150
130
110
90
80
70
60
50
40
30
20
10
0
6500
6000
5500
5000
4500
4000
3500
3000
2500
2000
1500
1000
500
0
